# Targeting Histone Deacetylase 11 with a Highly Selective Inhibitor for the Treatment of MASLD

**DOI:** 10.1002/advs.202412903

**Published:** 2025-02-20

**Authors:** Feng Zhang, Kairui Yue, Simin Sun, Shengyuan Lu, Geng Jia, Yang Zha, Shuang Zhang, C. James Chou, Chenzhong Liao, Xiaoyang Li, Yajun Duan

**Affiliations:** ^1^ Department of Cardiology the First Affiliated Hospital of USTC Division of Life Sciences and Medicine University of Science and Technology of China Hefei 230001 China; ^2^ Key Laboratory of Metabolism and Regulation for Major Diseases of Anhui Higher Education Institutes Hefei University of Technology Hefei 230031 China; ^3^ Key Laboratory of Marine Drugs Chinese Ministry of Education School of Medicine and Pharmacy Ocean University of China 5 Yushan Road Qingdao 266003 China; ^4^ Department of Drug Discovery and Biomedical Sciences College of Pharmacy Medical University of South Carolina Charleston SC 29425 USA; ^5^ Marine Biomedical Research Institute of Qingdao Qingdao Shandong 266071 China

**Keywords:** AMP‐activated protein kinase, de novo lipogenesis, fatty acid oxidation, histone deacetylase 11, metabolic dysfunction‐associated steatotic liver disease

## Abstract

Metabolic dysfunction‐associated steatotic liver disease (MASLD) represents the most prevalent chronic liver disorder globally. Due to its intricate pathogenesis and the current lack of efficacious pharmacological interventions, there is a pressing need to discover novel therapeutic targets and agents for MASLD treatment. Herein, it is found that histone deacetylase 11 (HDAC11), a subtype of HDAC family, is markedly overexpressed in both in vitro and in vivo models of MASLD. Furthermore, the knockdown of HDAC11 is observed to mitigate lipid accumulation in hepatic cells. A highly selective HDAC11 inhibitor, **B6**, which exhibits favorable pharmacokinetic property and liver distribution, is further designed and synthesized. Integrating RNA‐seq data with in vivo and in vitro experiments, **B6** is found to inhibit de novo lipogenesis (DNL) and promote fatty acid oxidation, thus mitigating hepatic lipid accumulation and pathological symptoms in MASLD mice. Further omics analysis and experiments reveal that **B6** enhances the phosphorylation of AMPKα1 at Thr172 through the inhibition of HDAC11, consequently modulating DNL and fatty acid oxidation in the liver. In summary, this study identifies HDAC11 as a potential therapeutic target in MASLD and reports the discovery of a highly selective HDAC11 inhibitor with favorable drug‐like properties for the treatment of MASLD.

## Graphical abstract

A potent HDAC11‐specific inhibitor, B6, is identified using substrate‐mimic and bioisosteric replacement strategies. B6 demonstrates high oral bioavailability, extensive liver distribution, and a favorable safety profile. B6 effectively alleviates metabolic dysfunction‐associated steatotic liver disease by increasing the expression of LKB1 and enhancing the phosphorylation of AMPKα at Thr172, thereby reducing de novo lipogenesis and promoting fatty acid oxidation.

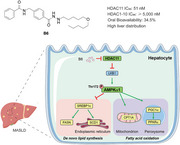

## 1 Introduction

Metabolic dysfunction‐associated steatotic liver disease (MASLD) is a chronic liver disease characterized by the accumulation of excess fat in the liver, independent of excessive alcohol consumption.^[^ ^1^ ^]^ The spectrum of MASLD ranges from simple metabolic dysfunction‐associated steatohepatitis (MASH).^[^ ^2^ ^]^ As the disease progresses, it may further develop into liver fibrosis, cirrhosis, and even hepatocellular carcinoma (HCC).^[^ ^3^ ^]^ The pathological mechanisms underlying the occurrence and progression of MASLD are exceedingly complex and remain incompletely elucidated so far and there are still limited approved pharmacological treatments specifically targeting MASLD.

The primary mechanisms contributing to hepatic lipid accumulation include excessive lipid intake, de novo lipogenesis (DNL), fatty acid oxidation, and lipid efflux.^[^ ^4^ ^]^ Excessive lipid intake enhances DNL, with acetyl‐CoA carboxylase (ACC), fatty acid synthase (FASN), and stearoyl‐CoA desaturase 1 (SCD1) serving as key enzymatic regulators in this process.^[^ ^5^ ^]^ The transcription factor responsible for regulating FASN and SCD1 to control DNL is sterol regulatory element binding protein 1c (SREBP1c).^[^ ^6^ ^]^ Studies have demonstrated that hepatocyte‐specific overexpression of SREBP1c results in the upregulation of these critical DNL enzymes, consequently leading to hepatic lipid accumulation.^[^ ^7^ ^]^ Fatty acid oxidation predominantly occurs in the mitochondria, with the entry of fatty acids into the mitochondria facilitated by carnitine palmitoyl transferase 1A (CPT1A), which is located in the outer mitochondrial membrane.^[^ ^8^ ^]^ One of the principal regulators of CPT1A is peroxisome proliferator‐activated receptor‐α (PPARα).^[^ ^9^ ^]^ Mice with hepatocyte‐specific knockout of PPARα exhibit significant steatosis, inflammation, and reduced level of CPT1A compared to wild‐type mice.^[^ ^10^ ^]^ In addition to fatty acid oxidation, the efflux of triglycerides constitutes another crucial pathway in the regulation of hepatic lipid metabolism. The primary components involved in this process are apolipoprotein B100 and microsomal triglyceride transfer protein.^[^ ^11^ ^]^

Histone deacetylases (HDACs) are enzymes that catalyze the removal of acetyl functional groups from lysine residues on both histone and nonhistone proteins.^[^ ^12^ ^]^ In mammals, HDACs are classified into four distinct classes based on their structural and functional characteristics: class I Rpd3‐like proteins (HDAC1‐3 and 8); class II Hda1‐like proteins (HDAC4‐7, 9, and 10); class III Sir2‐like proteins (SIRT1‐7); and class IV, which currently includes only HDAC11 so far.^[^ ^12^ ^]^

HDAC11 is the sole member of the recently identified class IV HDAC subfamily,^[^ ^12^ ^]^ and its physiological functions remain to be unveiled. Previous studies have indicated that HDAC11 possesses weak deacetylase activity but exhibits robust fatty‐acid deacylation activity. Specifically, it hydrolyzes aliphatic lysine residues with longer chain lengths, such as dodecanoyl lysine and myristoyl lysine (C12–C14).^[^ ^13^ ^]^ To date, only two non‐histone substrates of HDAC11 have been identified: serine hydroxymethyl transferase 2 (SHMT2) and gravin‐α/A kinase–anchoring protein 12.^[^ ^14^ ^]^ Recent studies have demonstrated that HDAC11 is implicated in obesity through its regulation of uncoupling protein 1 (UCP1) expression, which governs the thermogenic program of adipose tissue.^[^ ^15^ ^]^ As reported by Lei Sun et al., HDAC11 knockout mice exhibit better resistance to high‐fat diet induced obesity.^[^ ^16^ ^]^ HDAC11 deficiency not only enhances the expression and activity of UCP1 in brown adipose tissue, but also activates the adiponectin‐AdipoR‐AMPK pathway in the liver, suggesting HDAC11 may play a role in liver steatosis. These findings underscore HDAC11 as a pivotal regulator of energy homeostasis and suggest a potential association between HDAC11 and other metabolic disorders, such as MASLD. However, the precise mechanisms of how HDAC11 regulates MASLD remain unclear and have yet to be elucidated in the literature. In addition, the potential of HDAC11 as a drug target awaits further exploration. In recent years, the variety of reported HDAC11 inhibitors have been limited, such as the cyclic peptide Trapoxin A analogue TD034 and the hydroxamic acid compound PB94.^[^ ^17^ ^]^ These compounds showed promising HDAC11 inhibitory activity, but their isoform selectivity druggability still need to be optimized. Therefore, it is imperative to elucidate the role of HDAC11 in MASLD and to develop HDAC11 inhibitors with enhanced potency, selectivity and favorable drug‐like properties.

In this study, HDAC11 was identified as a potential therapeutic target for MASLD. Elevated expression of HDAC11 was observed in both in vitro and in vivo MASLD models, and knockdown of HDAC11 resulted in the suppression of intracellular lipid accumulation. We designed and synthesized a novel hydrazide‐based HDAC11 inhibitor, **B6**, which demonstrated high isoform selectivity, favorable pharmacokinetic properties, and significant liver distribution. Our findings demonstrate that the inhibition of HDAC11 by compound **B6** significantly mitigates high fat diet (HFD)‐induced MASLD. Specifically, HDAC11 inhibition facilitates the phosphorylation of AMP‐activated protein kinase α1 (AMPKα1) at Thr172, which consequently reduces DNL and enhances fatty acid oxidation. These results identify HDAC11 as a promising therapeutic target for MASLD, with the HDAC11 inhibitor **B6** emerging as a potential lead candidate due to its high selectivity and favorable pharmacological properties.

## 2 Results

### 2.1 Highly Expressed HDAC11 Was a Potential Target for MASLD

**Figure 1:**
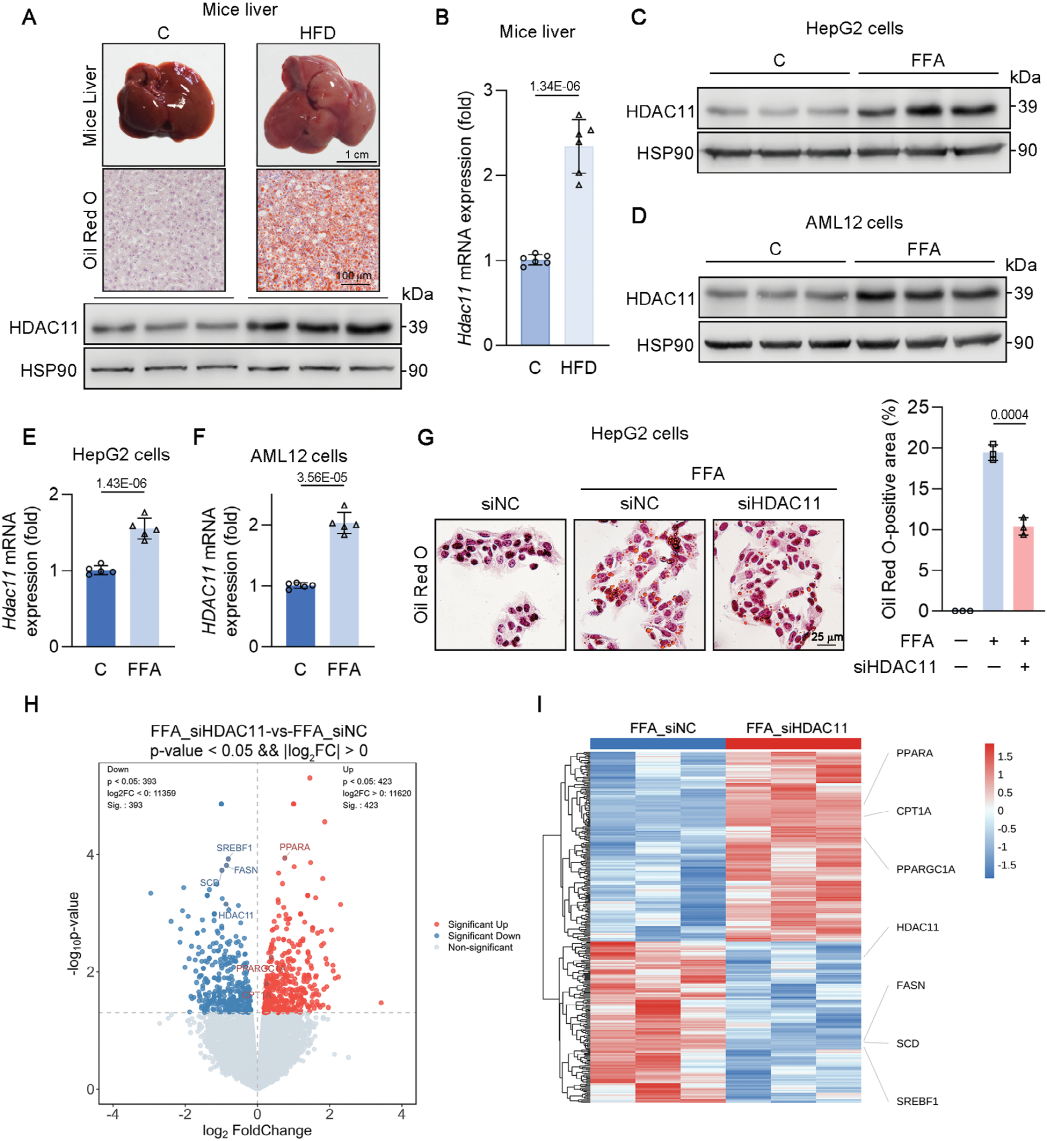
HDAC11 is involved in lipid accumulation in vivo and in vitro. A) Liver and Oil Red O staining images of mice, and hepatic HDAC11 protein expression (*n* = 6). B) mRNA expression of HDAC11 of mice liver (*n* = 6). C–F) Protein and mRNA expression of HDAC11 in FFA‐induced HepG2 and AML12 cells (*n* = 3 biological replicates). G) Oil Red O staining of HepG2 cells transfected with siHDAC11 and quantitative analysis. The experiment was conducted independently on three occasions. H) Volcano plot of differential gene expression between the FFA_siHDAC11 and FFA_siNC groups of HepG2 cells (*n* = 3). I) Heatmap analysis of bulk RNA‐seq data between the FFA_siHDAC11 and FFA_siNC groups of HepG2 cells (*n* = 3). Data are shown as mean ± SD. The *p*‐values were calculated by two‐tailed Student's *t*‐test.

Previous study has indicated HDAC11‐deficient mice exhibit enhanced thermogenic capacity and reduced obesity, further supporting the therapeutic potential of HDAC11 inhibition.^[^ ^16^ ^]^ In this study, we examined the role of HDAC11 in MASLD. A MASLD model was established by subjecting mice to a diet comprising 60% of calories from fat for a duration of 12 weeks, followed by the assessment of HDAC11 expression levels in hepatic tissues. As depicted in **Figure** **1**A,B, the high fat diet resulted in increased lipid accumulation and a significant upregulation of both protein and mRNA levels of HDAC11 in mice liver. To further investigate, we treated HepG2 and AML12 hepatocyte cell lines with free fatty acids (FFA) for 24 h to simulate MASLD in vitro. Consistent with the in vivo findings, FFA treatment led to an upregulation of HDAC11 protein and mRNA levels in both HepG2 and AML12 cells (Figure 1C–F). FFA induced substantial intracellular lipid accumulation, however, this effect was mitigated following the knockdown of HDAC11 in HepG2 cells (Figure 1G). To further investigate the involvement of HDAC11 in the pathogenesis of MASLD, RNA‐seq was performed on HepG2 cells with HDAC11 knockdown following FFA treatment. The analysis revealed significant alterations in the expression of genes associated with DNL (SREBF1, FASN, SCD) and fatty acid oxidation (PPARA, CPT1A, PPARGC1A), as shown in the volcano plot and heatmap (Figure 1H,I). These observations underscore the pivotal role of HDAC11 in lipid metabolism and suggest that targeted inhibition of HDAC11 could be a promising therapeutic strategy for addressing fatty liver disease.

### 2.2 **B6** Was Discovered as a Highly Potent HDAC11 Selective Inhibitor

**Figure 2:**
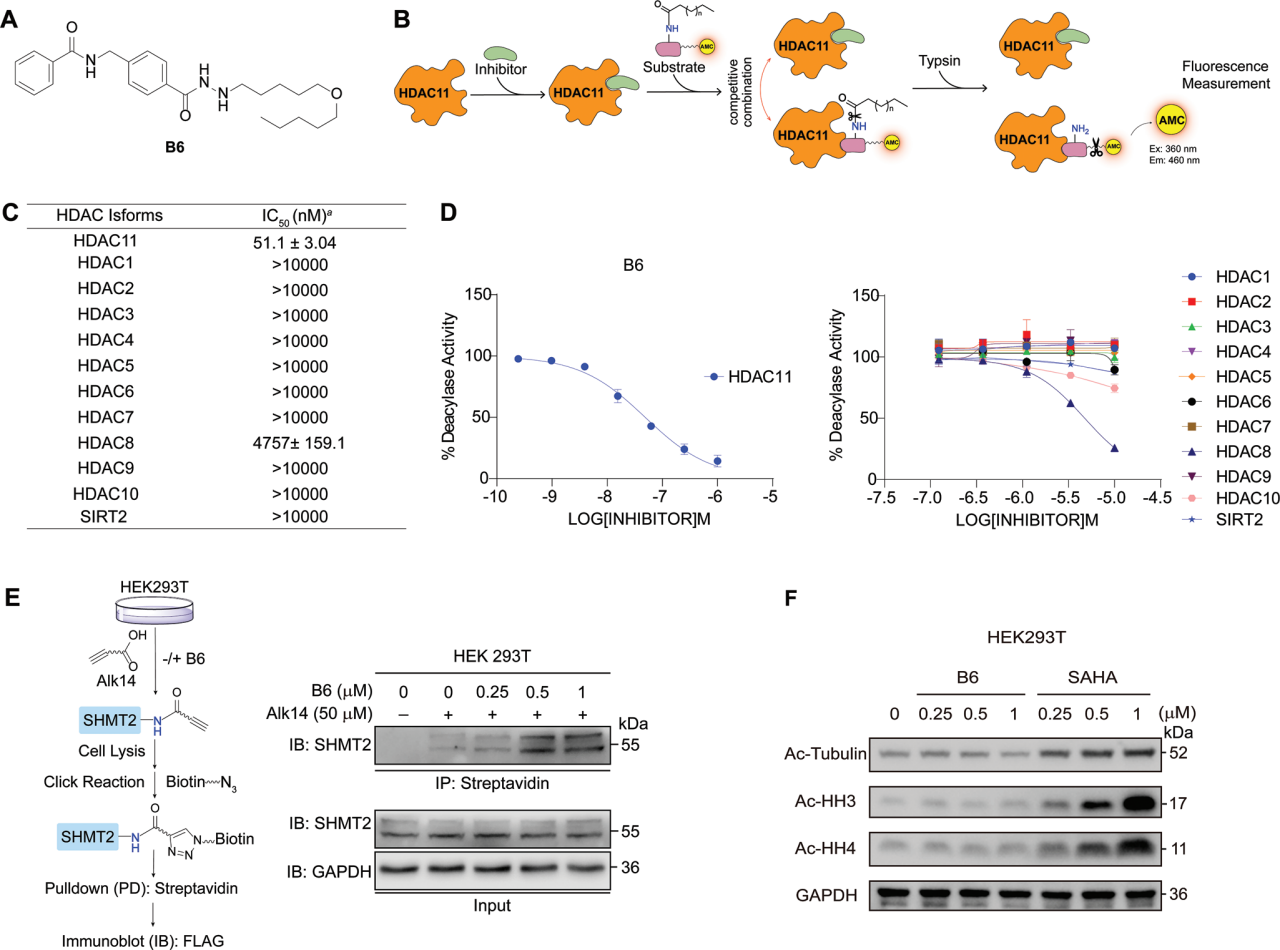
Discovery of HDAC11 selective inhibitor **B6**. A) Chemical structure of **B6**. B) Schematic depiction of the fluorescence assay for in vitro HDAC11 inhibition. C) *IC*_50_ values for the inhibitory activity of **B6** against HDAC1‐11 and SIRT2. Data are shown as mean ± SEM. D) *IC*_50_ curves of **B6** against HDAC1‐11 and SIRT2. The experiment was conducted independently on two occasions. Data are shown as mean ± SEM. E) Schematic depiction of the click chemistry of experiment to assess the myristoylation of SHMT2 influenced by **B6**. The myristoylation of SHMT2 was detected by immunoblotting in HEK293T cells treated with vehicle control (–) or 0.25, 0.5, and 1 × 10^−6^ m **B6** for 24 h (*n* = 3 biological replicates). F) The acetylation of histone H3, histone H4, and α‐tubulin in HEK293T cell after treatment of compounds **B6** and SAHA at the concentrations of 0.25, 0.5, and 1 × 10^−6^ m for 24 h (*n* = 3 biological replicates).

**Figure 3:**
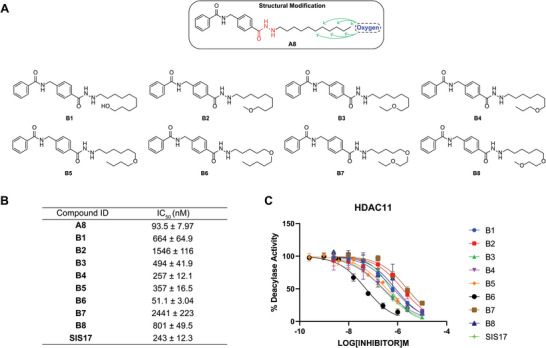
A) Compound modification strategy and chemical structure of **A8** and **B1**–**B8**. B) *IC*_50_ values for the inhibitory activity of **A8**, **B1**–**B8**, and SIS17 against HDAC11. The values were obtained from three independent experiments; data are shown as mean ± SEM. C) *IC*_50_ curves of **B1**–**B8** and SIS17 against HDAC11.

Given the association between HDAC11 and MASLD, we aim to design a potent and selective HDAC11 inhibitor. In addition, we developed a fluorescence‐based assay utilizing the HDAC11‐specific substrate ETDKmyr^[^ ^13b^ ^]^ to assess the inhibitory activity of potential HDAC11 inhibitors (**Figure** **2**B). In our prior research, we identified a class of efficient hydrazide‐based inhibitors, specifically **A1** and **A2**, which exhibit low nanomolar *IC*_50_ for HDAC1 and HDAC3 (Figure S1A, Supporting Information).^[^ ^18^ ^]^ Here, we found them demonstrating moderate inhibitory activity against HDAC11. Numerous studies have indicated that HDAC11 functions primarily as a defatty‐acylase enzyme rather than a deacetylase. The catalytic domain of HDAC11 possesses a long cavity that can accommodate long‐chain fatty acyl groups,^[^ ^13^ ^]^ presenting an opportunity to enhance both the isoform selectivity and inhibitory efficacy of molecules targeting HDAC11. Therefore, we selected compounds **A3**–**A7**, which have been previously documented and possess hydrazide tails comprising 6–10 carbon atoms, to evaluate their inhibitory activity against HDAC11. As anticipated, a progressive enhancement in the inhibitory activity of these compounds against HDAC11 was observed with increasing alkyl tail length, as evidenced by the decrease in *IC*_50_ values from 3312 × 10^−9^ to 416 × 10^−9^ m (**Table** **1**). However, the inhibitory activity of these compounds did not meet our research criteria. Consequently, we extended the carbon chain at the hydrazide tail, synthesizing compounds **A8** and **A9**, which feature 11 and 12 carbon chains, respectively. As anticipated, the HDAC11 inhibitory activity of the compounds improved, exhibiting *IC*_50_ values of 93.5 × 10^−9^ and 132 × 10^−9^ m, respectively (Table 1). The long hydrocarbon chains result in strong hydrophobicity for these two compounds, so in the subsequent compound modification, we attempt to introduce oxygen atoms into the alkyl chain of compound **A8** to enhance its hydrophilicity, thereby generating compounds **B1**–**B8** (**Figure** **3**). The presence of oxygen atoms in different positions along the long hydrocarbon chain results in varying inhibitory activities of the compounds against HDAC11. **B6** with oxygen at C5 position established highest HDAC11 inhibitory activity (*IC*_50_ of 51.1 × 10^−9^ m), which is slightly higher than its parent compound **A8** and significantly superior to the previously reported HDAC11 inhibitor SIS17.^[^ ^19^ ^]^ The introduction of two oxygen atoms will decrease the inhibitory activity of the compounds against HDAC11, as seen in compounds **B7** and **B8**. The incorporation of oxygen atom in compound **B6** improved the hydrophilicity of the parent compound **A8** (Figure S2, Supporting Information). **B6** exhibited an *IC*_50_ of 5 × 10^−6^ m for HDAC8 and showed no significant inhibitory activity against other HDAC subtypes at a concentration of 10 × 10^−6^ m, including SIRT2, which also features a long cavity in its active binding site (Figure 2C,D).^[^ ^20^ ^]^ This suggests that **B6** demonstrates isoform selectivity for HDAC11 over other HDAC isoforms. Treatment with **B6** resulted in a markedly increase in the myristoylation of SHMT2, a specific substrate of HDAC11, thereby confirming that **B6** directly targets HDAC11 (Figure 2E). Conversely, **B6** exhibited no effect on the acetylation levels of histones H3 and H4 (substrates of class I HDACs: HDAC1/2/3/8) or tubulin (a substrate of HDAC6) (Figure 2F). These results validate the isoform selectivity of **B6**. The predicted binding mode of **B6** with HDAC11 demonstrated a strong binding affinity (Figure S1B, Supporting Information). Specifically, the hydrazide moiety of **B6** bidentately chelated the zinc ion in HDAC11, while its long fatty chain effectively occupied the hydrophobic cavity situated at the base of the zinc ion within HDAC11.

**Table 1:** *IC*_50_ values for the inhibitory activity of **A1**–**A9** and SIS17 against HDAC1, 3, and 11.

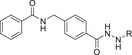				
Comp. ID	R	*IC*_50_ [nm]^a)^		
		HDAC11	HDAC1	HDAC3
**A1** ^[^ ^18^ ^]^	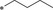	3247 ± 241	20.5 ± 1.40	18.1 ± 0.41
**A2** ^[^ ^18^ ^]^	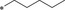	6741 ± 324	45.6 ± 2.36	24.6 ± 0.69
**A3** ^[^ ^18^ ^]^	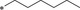	>10000	34.5 ± 3.15	6.79 ± 0.19
**A4** ^[^ ^18^ ^]^	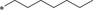	3312 ± 157	415 ± 153	6363 ± 324
**A5** ^[^ ^18^ ^]^	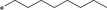	942 ± 94.1	2048 ± 27.5	501 ± 2.4
**A6** ^[^ ^18^ ^]^	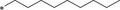	504 ± 45.5	>10000	2052 ± 39.5
**A7** ^[^ ^18^ ^]^	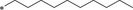	416 ± 41.1	>10000	>10000
**A8**	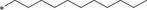	93.5 ± 7.97	>10000	>10000
**A9**	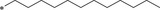	132 ± 4.29	>10000	>10000
**SIS17**		243 ± 12.3	>10000	>10000

^a)^ The values were obtained from two or three independent experiments; data are shown as mean ± SEM.

### 2.3 **B6** Exhibits Favorable Pharmacokinetic Properties and Is Highly Distributed in the Liver

**Figure 4:**
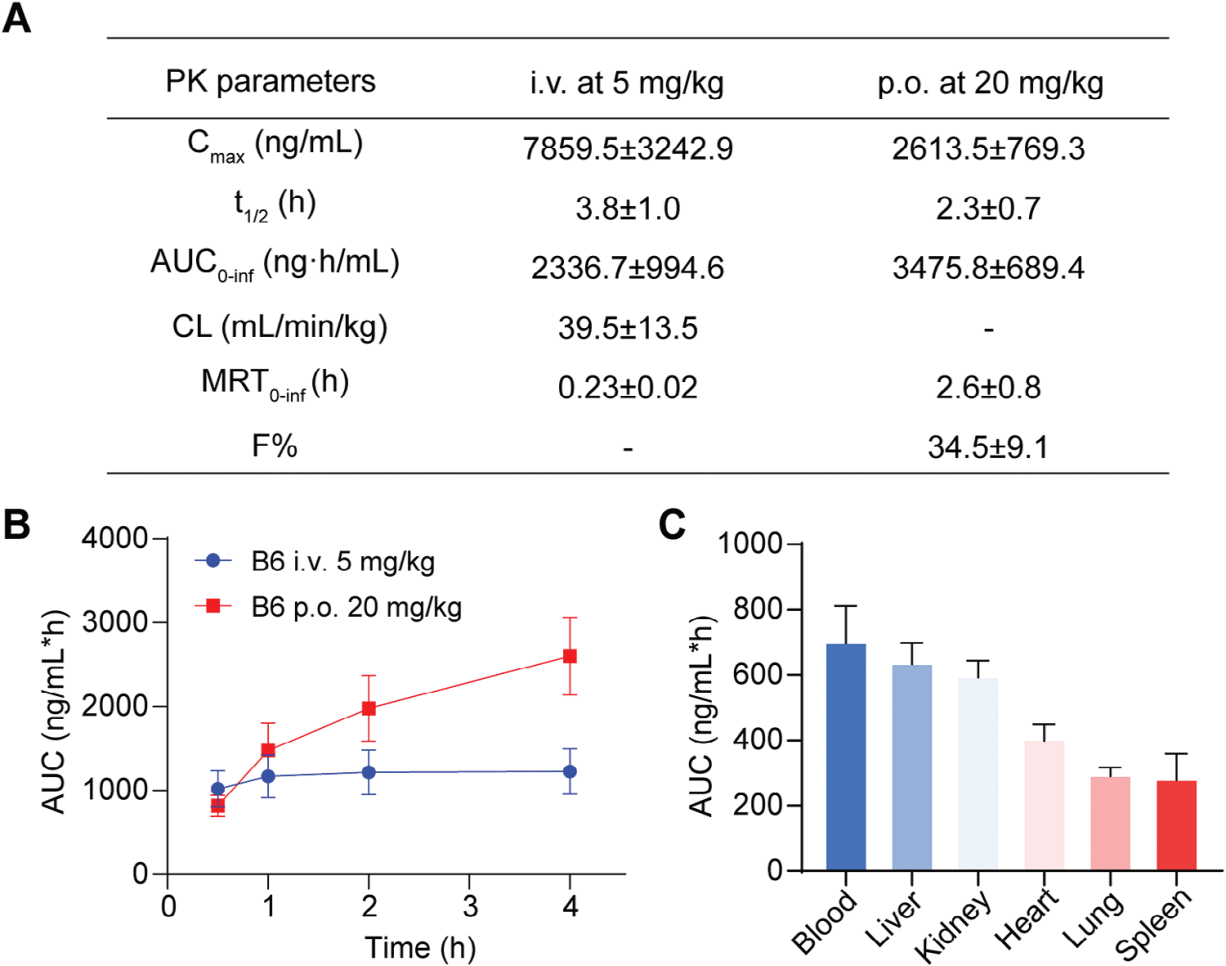
Pharmacokinetic and tissue distribution study of **B6**. A) Pharmacokinetic parameters of **B6** in ICR mice. **B6** was administrated via i.v. and p.o. (*n* = 3). B) The curves represent the AUC of plasma concentration versus time for **B6** following i.v. and p.o. at different time points (*n* = 3). C) The AUC values of concentration for **B6** were determined in blood, heart, liver, spleen, lung, and kidney tissues at 0.17, 0.5, and 1 h after administration (i.p. at 5 mg kg^−1^) (*n* = 3). Data are shown as mean ± SD.

The pharmacokinetic profile is a critical drug‐like attribute, influencing the route of administration in animal models. Therefore, we conducted a pharmacokinetic study of **B6** in IRC mice through both oral and intravenous (i.v.) administration (**Figure** **4**A). In the i.v. group (5 mg kg^−1^), the half‐life (*t*_1/2_), maximum plasma concentration (*C*_max_), and area under drug concentration–time curve (AUC_0‐inf_) values were 3. 8 h, 2336.7 ng mL^−1^, and 7859.5 h ng mL^−1^, respectively. In the oral administration group (20 mg kg^−1^), the *t*_1/2_ of **B6** was 2.3 h, with *C*_max_ and AUC_0‐inf_ values of 2613.5 ng mL^−1^ and 3475. 8 ng h mL^−1^, respectively, resulting in an oral bioavailability (F%) of 34.5%. To investigate the relationship between HDAC11 and MASLD, we assessed the tissue distribution of **B6** in the liver. The results indicated that **B6** is extensively distributed across the examined tissues, with the highest concentrations observed in the liver and kidneys (Figure 4C). This suggests that **B6** can be used for in vivo studies of liver diseases.

### 2.4 **B6** Alleviates FFA‐Induced Lipid Accumulation In Vitro

**Figure 5:**
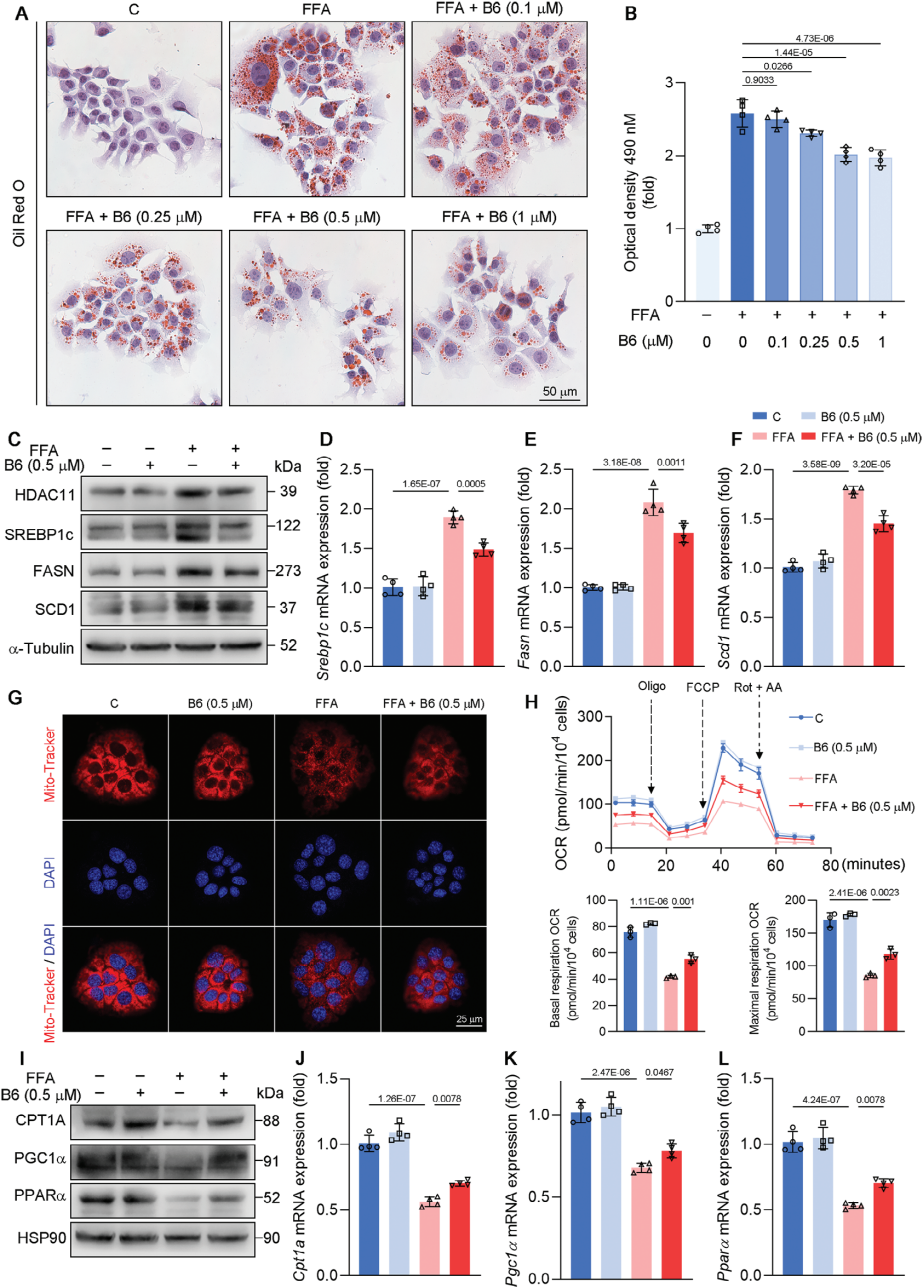
Effects of **B6** on de novo lipogenesis and fatty acid oxidation in AML12 cells. A,B) Oil Red O staining and optical density of AML12 cells treated with **B6** of different concentration for 24 h. The experiment was conducted independently on four occasions. C) Protein expression of HDAC11, SREBP1c, FASN, SCD1, and HSP90 of HepG2 cells treated with FFA and 0.5 × 10^−6^ m **B6** for 24 h (*n* = 3 biological replicates). D–F) Relative normalized mRNA expression of SREBP1c, FASN, and SCD1 of indicated cells (*n* = 4 biological replicates). mRNA level of β‐actin was used as normalized control. G) Mito‐Tracker Deep Red FM staining of indicated cells. The experiment was conducted independently on three occasions. H) Mitochondrial oxygen consumption rate in indicated cells (*n* = 3 biological replicates). I) Protein expression of CPT1A, PGC1α, PPARα, and HSP90 in indicated cells (*n* = 3 biological replicates). J–L) Relative normalized mRNA expression of CPT1A, PGC1α, and PPARα of indicated cells (*n* = 4 biological replicates). mRNA level of β‐actin was used as normalized control. Data are shown as mean ± SD. The *p*‐values were calculated by one‐way ANOVAs.

Given that **B6** was identified as a potent selective inhibitor of HDAC11, we assessed its efficacy in the in vitro models of MASLD established with both HepG2 (Figure S3, Supporting Information) and AML12 cells (**Figure** **5**). As illustrated in Figure 5A,B, treatment with various concentrations of **B6** significantly mitigated FFA‐induced lipid accumulation in AML12 cells, with maximal effect observed at a concentration of 0.5 × 10^−6^ m (Figure 5C). Comparable reduction in lipid accumulation was also evident in HepG2 cells (Figure S3A, Supporting Information). DNL is the metabolic pathway responsible for synthesizing saturated and monounsaturated fatty acids from acetyl‐CoA.^[^ ^21^ ^]^ An increase in DNL facilitates the accumulation of lipid droplets. SREBP1c serves as a pivotal transcription factor in DNL, regulating the downstream enzyme FASN.^[^ ^22^ ^]^ Saturated fatty acids synthesized by FASN are subsequently catalyzed by SCD1 to produce monounsaturated fatty acids, which further promote the formation of lipid droplets.^[^ ^23^ ^]^ As illustrate din Figure 5C–F, FFA upregulated the protein and mRNA expression of SREBP1c, FASN, and SCD1; however, this effect was reversed following treatment with **B6**. When the liver accumulates an excess of lipids, mitochondrial fatty acid oxidation becomes particularly crucial.^[^ ^24^ ^]^ We evaluated the mitochondrial activity of AML12 cells using Mito‐Tracker staining (Figure 5G). The results demonstrated that FFA significantly inhibited mitochondrial activity. However, this inhibition was ameliorated upon treatment with **B6**. Furthermore, we performed mitochondrial stress tests on AML12 cells (Figure 5H). The data revealed that both basal respiration and maximal respiration, as measured by the oxygen consumption rate (OCR), were suppressed by FFA, indicating impaired mitochondrial function. Notably, treatment with **B6** partially restored mitochondrial activity in AML12 cells. CPT1A functions as a pivotal regulatory enzyme in the mitochondrial long‐chain fatty acid oxidation pathway, initiating the process of mitochondrial fatty acid oxidation.^[^ ^25^ ^]^ PPAR gamma co‐activator 1α (PGC1α) is recognized as the principal transcriptional regulator of mitochondrial biogenesis, facilitating an augmentation in mitochondrial quantity, enhancing fatty acid oxidation, and contributing to energy production, thereby playing a crucial role in the maintenance of cellular energy homeostasis.^[^ ^26^ ^]^ In the liver, activation of PPARα activation stimulates fatty acid oxidation, culminating in elevated energy production.^[^ ^27^ ^]^ Figure 5I–L demonstrated that both the protein and mRNA expression of CPT1A, PGC1α, and PPARα were suppressed by FFA, but were partially restored upon treatment with **B6**. **B6** exhibited similar effects on lipid synthesis and fatty acid oxidation metabolism in HepG2 cells as observed in AML12 cells (Figure S3C–L, Supporting Information). These findings suggest that **B6** mitigates FFA‐induced lipid accumulation by inhibiting DNL and promoting fatty acid oxidation.

### 2.5 **B6** Regulates DNL and Fatty Acid Oxidation by Inhibiting HDAC11

**Figure 6:**
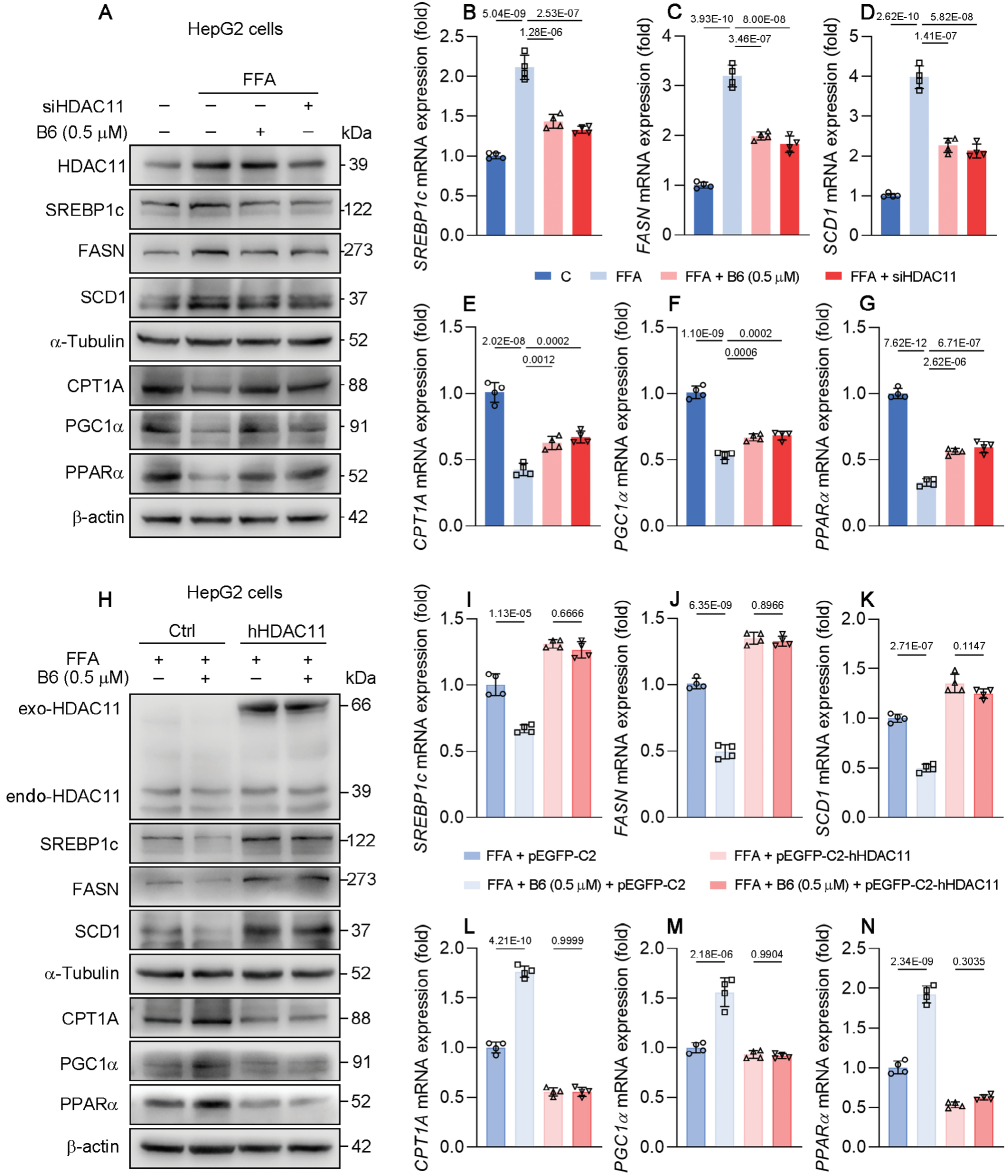
**B6** reduces de novo lipogenesis and promotes fatty acid oxidation through HDAC11. A) Protein expression of HDAC11, SREBP1c, FASN, SCD1, α‐Tubulin, CPT1A, PGC1α, PPARα, and HSP90 of HepG2 cells transfected with siHDAC11 and treated with 0.5 × 10^−6^ m **B6** for 24 h (*n* = 3 biological replicates). B–G) Relative normalized mRNA expression of SREBP1c, FASN, SCD1, CPT1A, PGC1α, and PPARα of indicated cells (*n* = 4 biological replicates). mRNA level of β‐actin was used as normalized control. H) Protein expression of exo‐HDAC11, endo‐HDAC11, SREBP1c, FASN, SCD1, α‐Tubulin, CPT1A, PGC1α, PPARα, and β‐actin of HepG2 cells transfected with pEGFP‐C2‐hHDAC11 and treated with 0.5 × 10^−6^ m **B6** for 24 h (*n* = 3 biological replicates). I–N) Relative normalized mRNA expression of SREBP1c, FASN, SCD1, CPT1A, PGC1α, and PPARα of indicated cells (*n* = 4 biological replicates). mRNA level of β‐actin was used as normalized control. Data are shown as mean ± SD. The *p*‐values were calculated by one‐way ANOVAs.

From the above results, we observed that **B6** can inhibit DNL and promote fatty acid oxidation. To further explore whether **B6** exerts these functions through the regulation of HDAC11, human cell line HepG2 was selected for follow‐up experiments. HepG2 cells induced with FFA were treated with **B6** or subjected to HDAC11 knockdown via siRNA. The results indicated that both **B6** treatment and HDAC11 knockdown produced analogous effects on the regulation of genes involved in DNL and fatty acid oxidation. Specifically, both interventions resulted in a reduction of protein and mRNA expression of SREBP1c, FASN, and SCD1 in FFA‐induced HepG2 cells. In addition, they also led to an upregulation of protein and mRNA expression of CPT1A, PGC1α, and PPARα (**Figure** **6**A–G). Furthermore, we transfected hHDAC11 into HepG2 cells. We observed that when the plasmid vector was transfected, **B6** could still effectively regulate the expression of genes related to DNL and fatty acid oxidation. However, upon transfection with hHDAC11, the regulatory effects of **B6** on the proteins and mRNA of SREBP1c, FASN, SCD1, CPT1A, PGC1a, and PPARα were diminished or abolished (Figure 6H–N). The above results indicate that **B6** regulates genes involved in DNL and fatty acid oxidation through the inhibition of HDAC11.

### 2.6 **B6** Alleviates Pathological Symptoms in MASLD Mice

**Figure 7:**
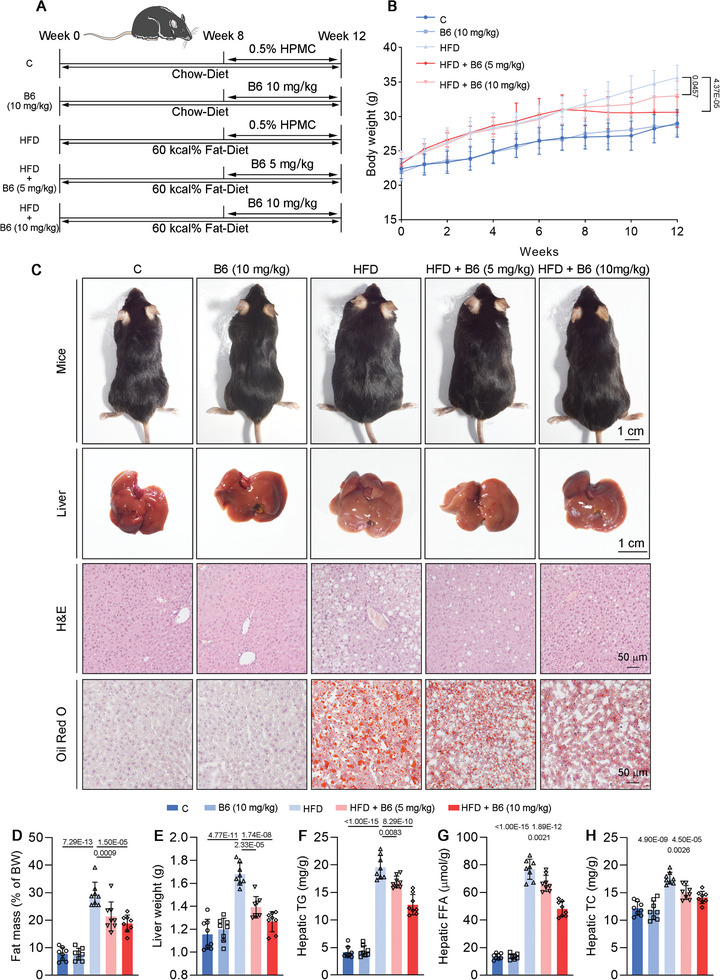
Effects of **B6** on mice fed with high fat diet. A) Schematic diagram of animal experiments. B) Weight change and fat mass of mice (*n* = 8). C) Images of mice and livers, and H&E and Oil red O staining of mice liver (*n* = 8). D,E) The fat mass and liver weight of mice (*n* = 8). F–H) Hepatic TG, TC, and FFA levels of mice (*n* = 8). Data are shown as mean ± SD. The *p*‐values were calculated by one‐way ANOVAs.

**Figure 8:**
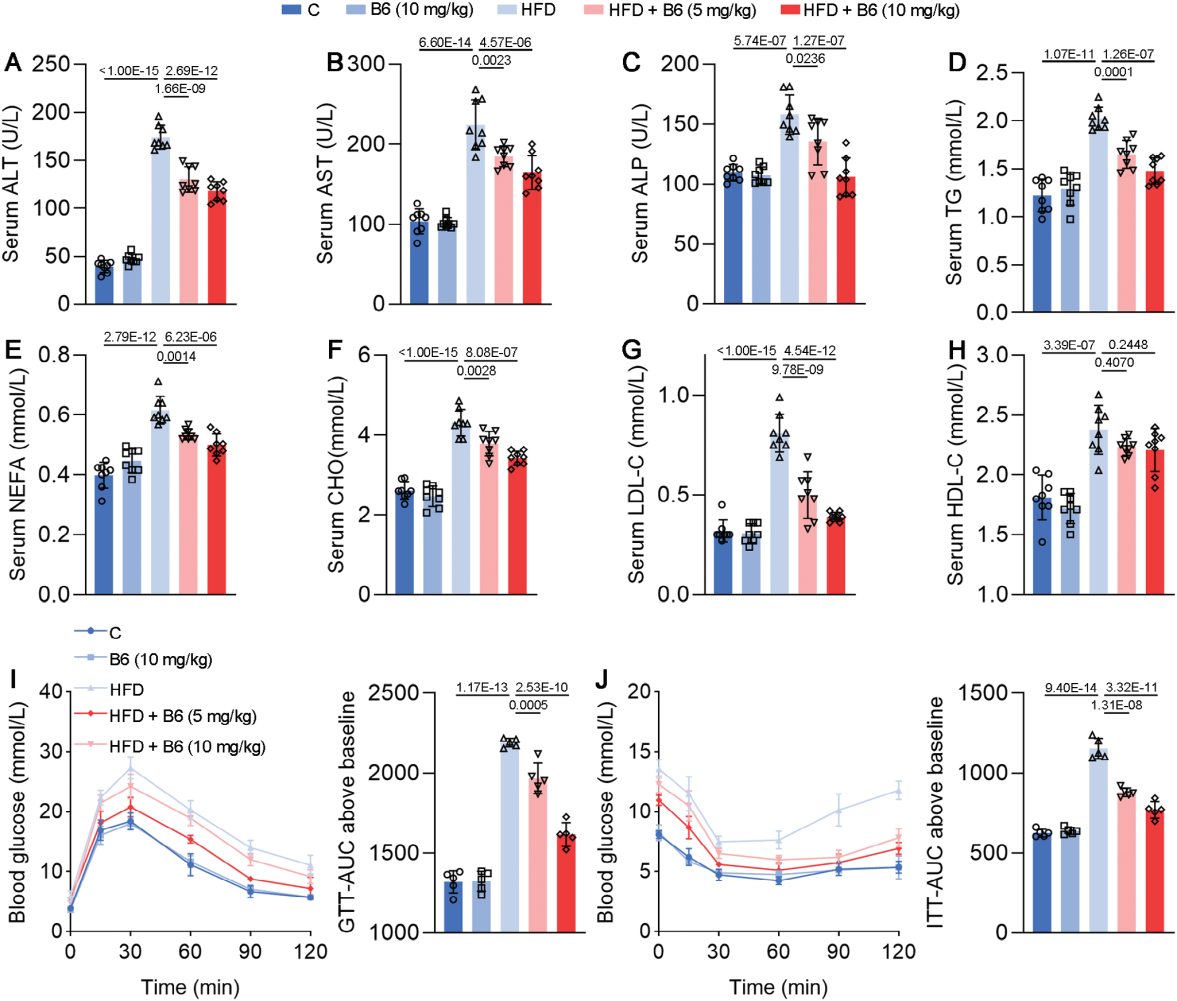
Effects of **B6** on serum indexes in mice fed with high fat diet. A–H) Serum ALT, AST, ALP, TG, NEFA, CHO, LDL‐C, and HDL‐C levels (*n* = 8). I,J) Glucose tolerance test and insulin tolerance test of mice (*n* = 5). Data are shown as mean ± SD. The *p*‐values were calculated by one‐way ANOVAs.

After demonstrating that **B6** exerts a significant effect on DNL and fatty acid oxidation processes in vitro, we established a MASLD model in mice to investigate the in vivo effects of **B6**. Given **B6**’s favorable oral absorption properties (Figure 4A), we opted for oral administration in the animal experiments. As shown in **Figure** **7**A, the mice were divided into five groups: the C group and **B6** (10) group were fed a chow diet, while the H group, H**B6** (5) group, and H**B6** (10) group were fed a 60% 60 kcal% fat diet. The mice were subjected to a 12‐week feeding regimen, with the final 4 weeks involving drug administration to simulate a therapeutic dosing schedule. The C group and H group were orally gavaged with 0.5% HPMC, while the **B6** (10) group, H**B6** (5) group, and H**B6** (10) group were orally gavaged with 10, 5, and 10 mg kg^−1^ of **B6** (0.5% HPMC served as the vehicle), respectively. As illustrated in Figure 7B, there were no significant differences in body weight between the C group and the **B6** (10) group, nor were there differences in food intake (Figure S4, Supporting Information). During the initial 8‐week period, mice subjected to a high fat diet exhibited a higher weight gain compared to those in the chow‐diet group. However, subsequent treatment with **B6** resulted in a reduction in body weight, exhibiting a dose‐dependent response. Specifically, mice in the H group displayed a significantly larger body size compared to the C group. Administrations of 5 or 10 mg kg^−1^ **B6** led to a reduction in body size among the H group (Figure 7C). Similarly, mice in the H group exhibited larger and heavier livers and increased body fat mass. Nevertheless, these parameters were effectively controlled following **B6** treatment, with higher doses yielding greater efficacy (Figure 7C–E). In Figure 7C, H&E staining along with Oil Red O staining of liver sections indicated severe lipid accumulation in the liver of the H group. In accordance, hepatic levels of triglycerides (TG), FFA, and total cholesterol (TC) further corroborated the pronounced lipid accumulation in the liver of the H group (Figure 7F–H). Treatment with **B6** resulted in a dose‐dependent reduction in hepatic lipid content. In addition, serum levels of alanine aminotransferase (ALT), aspartate aminotransferase (AST), and alkaline phosphatase (ALP)—key biomarkers for assessing liver damage—were measured (**Figure** **8**A–C).^[^ ^28^ ^]^ The results indicated that **B6** could alleviate liver damage induced by high fat diet. Moreover, **B6** demonstrated the capacity to reduce the elevated levels of blood lipids induced by high fat diet, including non‐esterified fatty acid (NEFA), cholesterol (CHO), and low‐density lipoprotein cholesterol (LDL‐C), while exhibiting no significant effect on high‐density lipoprotein cholesterol (HDL‐C) (Figure 8D–H). Concurrently, Figure 8I,J illustrates that **B6** also improved glucose tolerance and insulin resistance in HFD mice. These results indicate that **B6** confers resistance to obesity induced by high fat diet. In summary, **B6** exhibits beneficial effects on the pathological symptoms of MASLD in mice, particularly in the high‐dose group.

### 2.7 **B6** Reduces DNL and Promotes Fatty Acid Oxidation in MASLD Mice Liver

**Figure 9:**
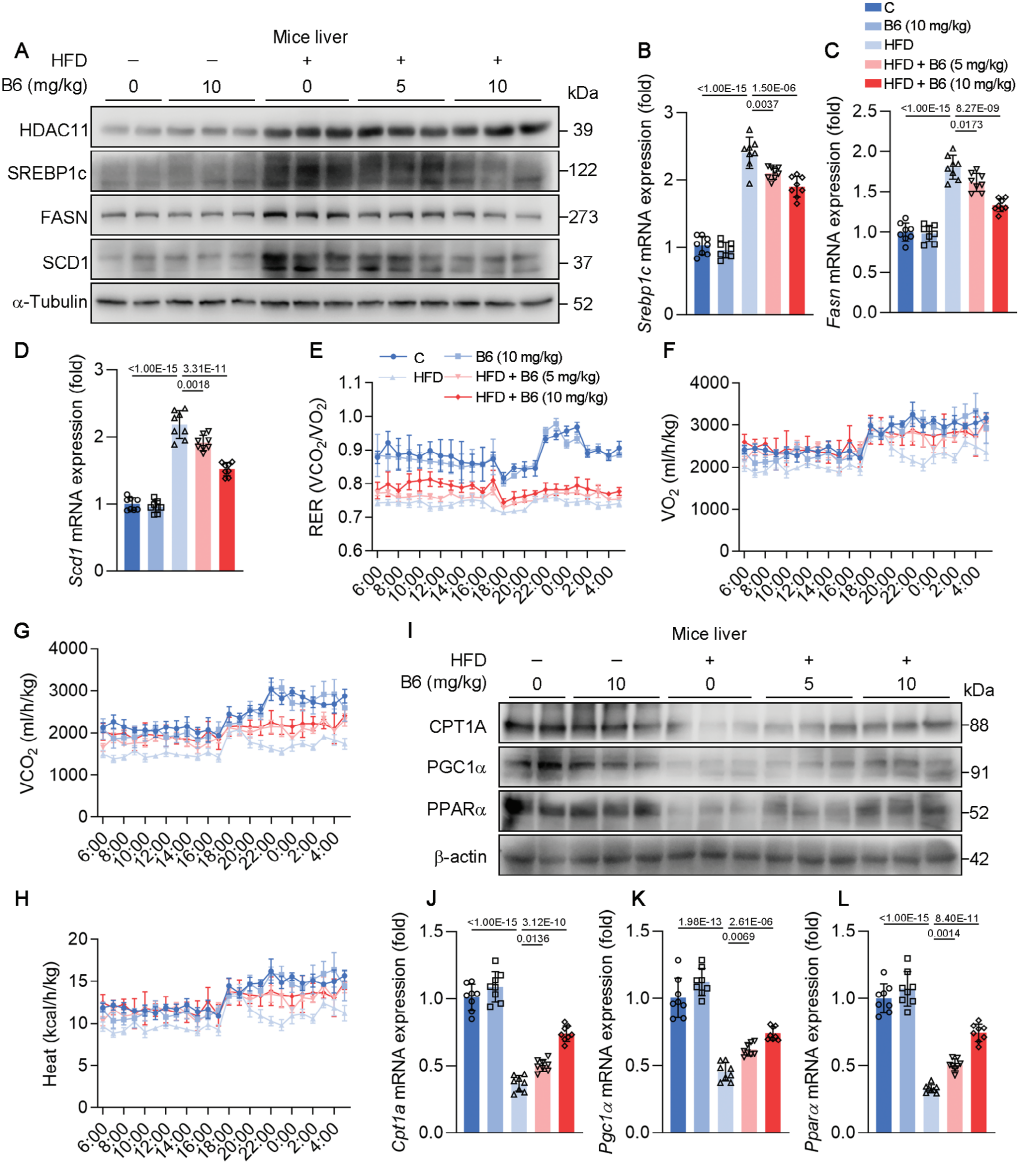
**B6** reduces de novo lipogenesis, increases energy consumption, and promotes fatty acid oxidation in HFD mice. A) Protein expression of HDAC11, SREBP1c, FASN, SCD1, and HSP90 of mice liver (*n* = 8). B–D) Relative normalized mRNA expression of Srebp1c, Fasn, and Scd1 in mice liver (*n* = 8). mRNA level of β‐actin was used as normalized control. E–H) Respiratory exchange ratio, O_2_ consumption, CO_2_ production, and heat production levels of mice (*n* = 5). I) Protein expression of CPT1A, PGC1α, PPARα, and HSP90 of mice liver (*n* = 8). J–L) Relative normalized mRNA expression of *Pgc1α*, *Cpt1a*, and *Pparα* in mice liver (*n* = 8). mRNA level of β‐actin was used as normalized control. Data are shown as mean ± SD. The *p*‐values were calculated by one‐way ANOVAs.

The preliminary findings from animal experiments indicate that **B6** can reduce lipid accumulation in the liver. To elucidate the mechanisms underlying the actions of **B6**, we examined results from in vitro studies and investigated the processes of DNL and fatty acid oxidation in the liver. Consistent with in vitro findings, we observed significant upregulation of the protein and mRNA expression of SREBP1c, FASN, and SCD1 in response to high fat diet. However, these indexes exhibited a dose‐dependent downregulation upon treatment with **B6** (**Figure** **9**A–D). This indicates that **B6** may ameliorate hepatic DNL. The augmentation of fatty acid oxidation plays a significant role in lipid metabolism, consequently diminishing lipid deposition within the body.^[^ ^29^ ^]^ To evaluate the impact of **B6** on metabolic parameters, mice were subjected to analysis in metabolic cages. The respiratory exchange ratio (RER) value serves as an indicator of the predominant fuel source utilized by the mice. Specifically, an RER of 0.70 signifies that fat is the primary fuel source, an RER of 0.85 denotes a combination of fat and carbohydrates, and an RER of 1.00 or higher indicates that carbohydrates are the predominant fuel source.^[^ ^30^ ^]^ The RER value of H group was significantly lower than that of C and B6 (10) groups, indicating a greater reliance on fat‐based energy sources in H group (Figure 9E). However, after **B6** treatment, there was a slight increase in RER value, indicating a shift toward a higher reliance on carbohydrate‐based energy sources (Figure 9E). This shift may be attributed to the significantly higher body fat content in the HFD mice compared to the **B6**‐treated mice, which results in fat becoming the predominant energy source. In addition, compared to H group, there was an observed increase in O_2_ consumption (VO_2_), CO_2_ production (VCO_2_), and heat after **B6** treatment, indicating that **B6** may enhance metabolism activity (Figure 9F–H). Subsequently, we examined the expression of genes associated with fatty acid oxidation. Both protein and mRNA levels of CPT1A, PGC1α, and PPARα were restored by **B6** compared to H group (Figure 9I–L). These findings indicate that **B6** can not only reduce DNL, but also improves metabolic parameters and promotes fatty acid oxidation in the liver of MASLD mice.

### 2.8 **B6** Targets HDAC11 to Reduce DNL, and Promote Fatty Acid Oxidation by Modulating the Phosphorylation of AMPKα at Thr172

**Figure 10:**
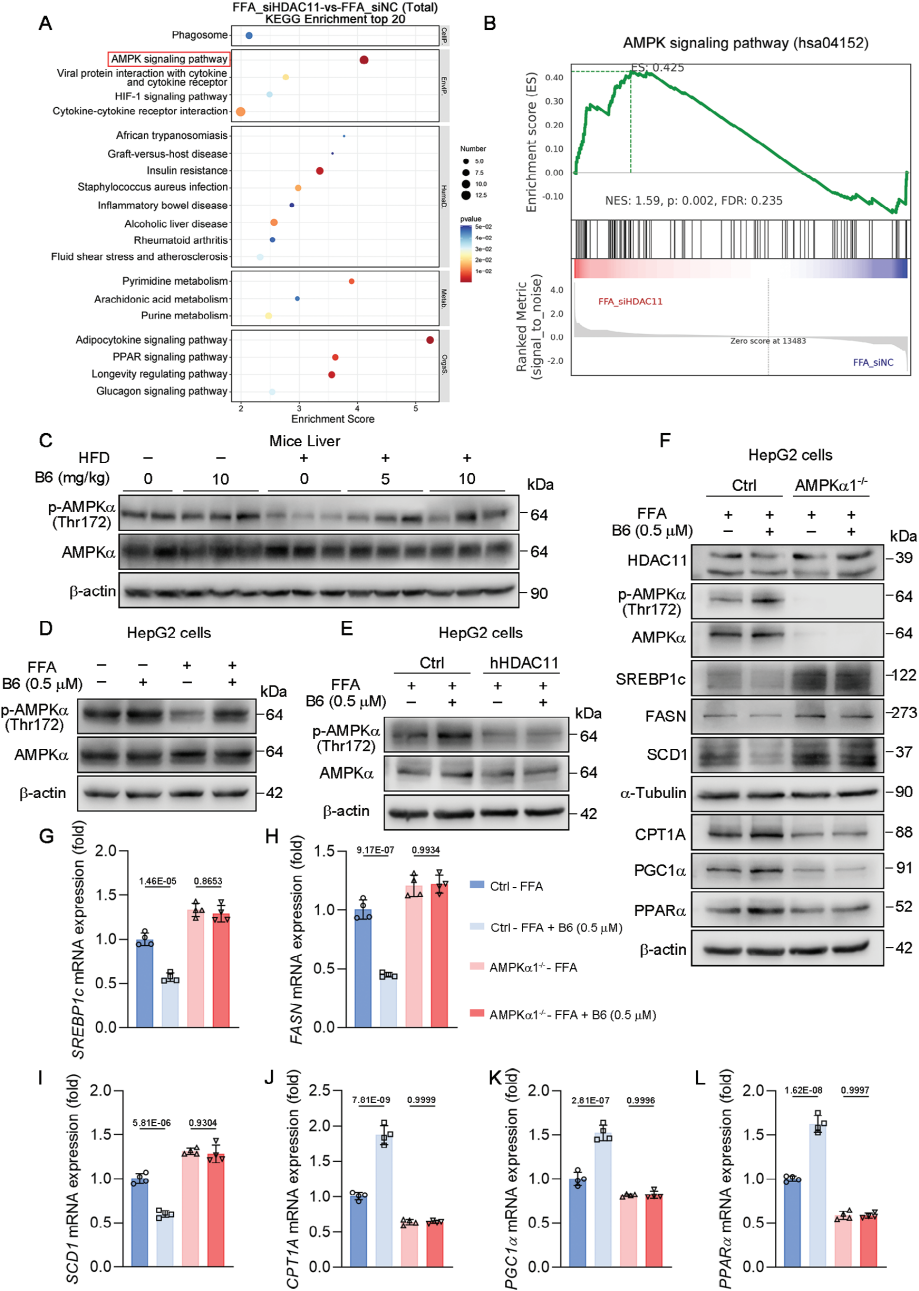
**B6** reduces de novo lipogenesis and promotes fatty acid oxidation by regulating phosphorylation of AMPKα1 through inhibiting HDAC11. A) Top 20 of total differential gene KEGG enrichment pathway between the FFA_siHDAC11 and FFA_siNC groups of HepG2 cells (*n* = 3). B) GSEA enrichment analysis graphs of AMPKα signaling pathway between the FFA_siHDAC11 and FFA_siNC groups of HepG2 cells (*n* = 3). C) Protein expression of *p*‐AMPKα (Thr172), AMPKα, and HSP90 of mice liver (*n* = 8). D) Protein expression of p‐AMPKα (Thr172), AMPKα, and HSP90 of HepG2 cells treated with FFA and 0.5 × 10^−6^ m **B6** for 24 h (*n* = 3 biological replicates). E) Protein expression of p‐AMPKα (Thr172), AMPKα, and α‐Tubulin of HepG2 cells transfected with pEGFP‐C2‐hHDAC11 and treated with 0.5 × 10^−6^ m **B6** for 24 h (*n* = 3 biological replicates). F) Protein expression of HDAC11, p‐AMPKα (Thr172), AMPKα, SREBP1c, FASN, SCD1, CPT1A, PGC1α, PPARα, and HSP90 of AMPKα1^−/−^ HepG2 cells treated with FFA and 0.5 × 10^−6^ m **B6** for 24 h (*n* = 3 biological replicates). G–L) Relative normalized mRNA expression of SREBP1c, FASN, SCD1, CPT1A, PGC1α, and PPARα of indicated cells (*n* = 4 biological replicates). mRNA level of β‐actin was used as normalized control. Data are shown as mean ± SD. The *p*‐values were calculated by one‐way ANOVAs.

**Figure 11:**
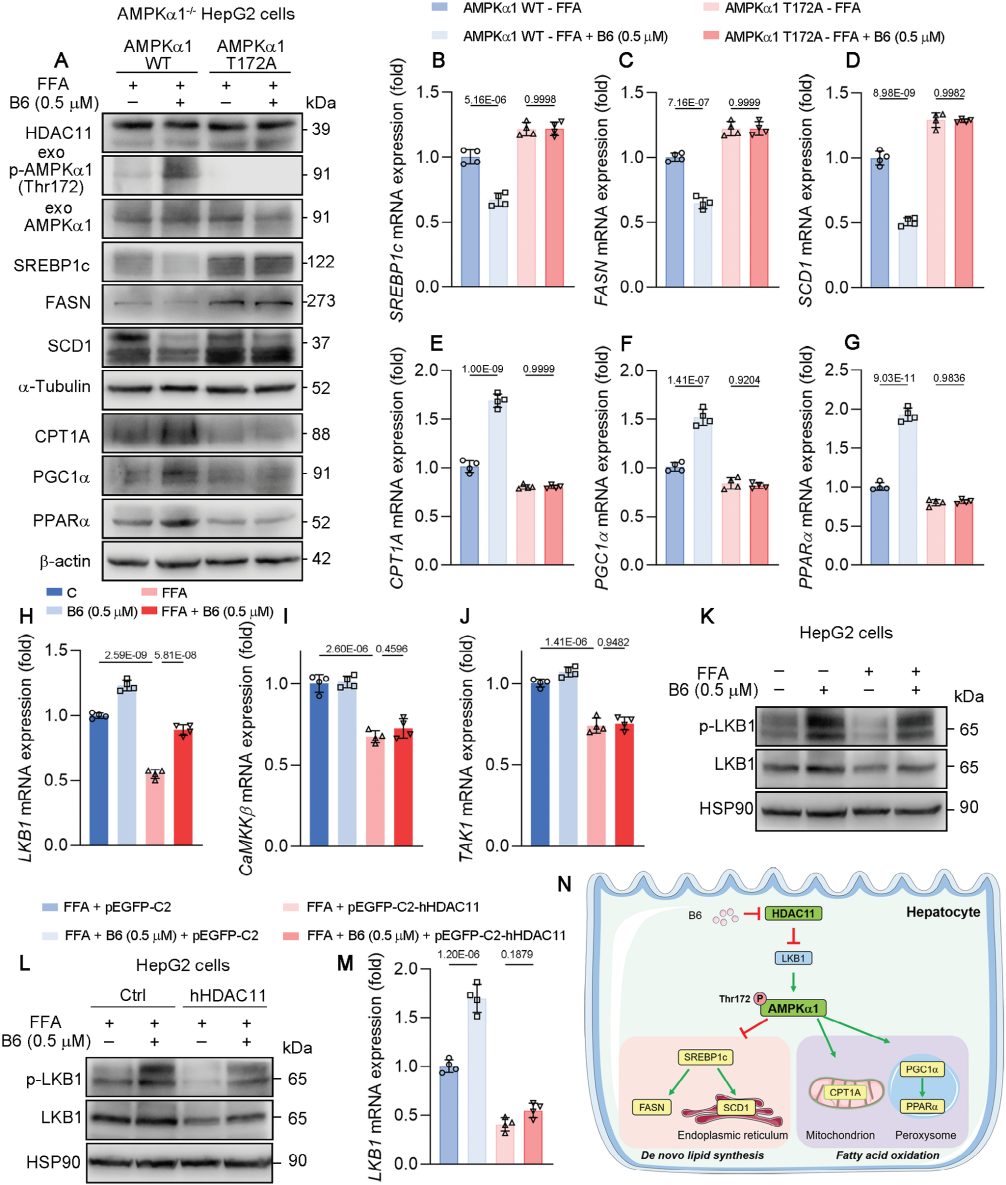
**B6** reduces de novo lipogenesis and promote fatty acid oxidation by modulating phosphorylation of AMPKα1 Thr172 through inhibiting HDAC11. A) Protein expression of HDAC11, exo‐p‐AMPKα (Thr172), exo‐AMPKα, SREBP1c, FASN, SCD1, α‐Tubulin, CPT1A, PGC1α, PPARα, and β‐actin of HepG2 cells transfected with AMPKα1‐WT and AMPKα1‐T172A, and treated with 0.5 × 10^−6^ m **B6** for 24 h (*n* = 3 biological replicates). B–J) Relative normalized mRNA expression of SREBP1c, FASN, SCD1, CPT1A, PGC1α, PPARα, LKB1, CaMKKβ, and TAK1 of indicated cells. mRNA level of β‐actin was used as normalized control (*n* = 4 biological replicates). K) Protein expression of p‐LKB1, LKB1, and HSP90 of HepG2 cells treated with FFA and 0.5 × 10^−6^ m **B6** for 24 h (*n* = 3 biological replicates). L) Protein expression of p‐LKB1, LKB1, and HSP90 of HepG2 cells transfected with pEGFP‐C2‐hHDAC11 and treated with 0.5 × 10^−6^ m **B6** for 24 h (*n* = 3 biological replicates). M) Relative normalized mRNA expression of LKB1 of indicated cells (*n* = 4 biological replicates). mRNA level of β‐actin was used as normalized control. N) Scheme of **B6** in alleviating NAFLD. **B6** inhibits HDAC11 and enhances phosphorylation of AMPKα1 Thr172, thereby alleviating de novo lipogenesis and promoting fatty acid oxidation in progression of NAFLD. Data are shown as mean ± SD. The *p*‐values were calculated by one‐way ANOVAs.

Previous studies have demonstrated that AMPK activation plays a critical role in MASLD by inhibiting hepatic DNL and enhancing hepatic fatty acid oxidation.^[^ ^31^ ^]^ In the RNA‐seq analysis of HepG2 cells with HDAC11 knockdown following FFA treatment, Kyoto Encyclopedia of Genes and Genomes (KEGG) enrichment analysis revealed significant enrichment of differentially expressed genes in the AMPK signaling pathway (**Figure** **10**A). Similarly, gene set enrichment analysis (GSEA) confirmed the significant enrichment of the AMPK signaling pathway (Figure 10B). Proteomic data further supported this finding, showing consistent enrichment in the AMPK signaling pathway (Figure S5, Supporting Information). Based on these results, we focused on validating the regulatory role of HDAC11 in the AMPK signaling pathway. One of the key mechanisms of AMPK activation is the phosphorylation of Thr172 on the α‐subunit.^[^ ^32^ ^]^ Recent studies have suggested that the loss of HDAC11 activates AMPK signaling pathway by promoting histone acetylation in the liver kinase B1 (LKB1) promoter region in HCC cell lines, thereby suppressing tumor stemness and the progression of HCC. In addition, another report demonstrated that the elimination of HDAC11 enhances mitochondrial fatty acid oxidation by activating the AMPK pathway and reducing intracellular carnitine levels in skeletal muscle, consequently improving muscle strength and fatigue resistance.^[^ ^33^ ^]^ Therefore, it is hypothesized that the inhibition of HDAC11 may exert its anti‐MASLD effects through the activating AMPK in the liver. We examined the impact of **B6** on the activation of AMPK in liver tissue and FFA‐treated HepG2 cells. As illustrated in Figure 10C,D, high fat diet suppresses the phosphorylation of AMPKα at Thr172 in both mouse liver and FFA‐treated HepG2 cells. In contrast, administration with **B6** restores the phosphorylation level of AMPKα at Thr172. To determine whether the activation of AMPKα by **B6** is mediated through HDAC11, we overexpressed HDAC11 in HepG2 cells (Figure 10E). The findings suggest that the overexpression of hHDAC11 inhibit the phosphorylation of AMPKα. Additionally, the effect of **B6** in activating the phosphorylation of AMPKα was diminished in cells overexpressing hHDAC11. These results indicate that the activation of AMPKα Thr172 phosphorylation by **B6** is mediated through HDAC11. Previous study has reported that complexes containing AMPKα1 and AMPKα2 account for approximately half of the total AMPK activity in the liver of rodents, whereas complexes with AMPKα1 play a predominant role in human liver cells.^[^ ^34^ ^]^ Therefore, we inferred that **B6** primarily regulates the phosphorylation of AMPKα1 through HDAC11. For this purpose, AMPKα1 knockout HepG2 cells were constructed for further study. In AMPKα1 knockout HepG2 cells, the downregulation of SREBP1c, FASN, and SCD1 by **B6**, as well as the upregulation of CPT1A, PGC1α, and PPARα, was abolished compared to the control group (Figure 10F–L). This result indicates that AMPKα1 is integral to the regulation of DNL and fatty acid oxidation by **B6** in HepG2 cells. Following this, AMPKα1‐WT or AMPKα1‐T172A was transfected into AMPKα1 knockout HepG2 cells for further investigation. Transfection with AMPKα1‐WT resulted in the activation of AMPKα1 Thr172 phosphorylation by **B6**, thereby restoring the regulatory effects of **B6** on DNL and fatty acid oxidation (**Figure** **11**A–G). Conversely, **B6** exhibited no regulatory effects on DNL and fatty acid oxidation in AMPKα1 knockout HepG2 cells transfected with AMPKα1 T172A. LKB1, Ca^2+^/calmodulin‐dependent protein kinase β (CaMKKβ) and transforming growth factor‐β (TGF‐β)‐activated kinase 1 (TAK1) have been identified as direct upstream kinases responsible for phosphorylate AMPK.^[^ ^35^ ^]^ In addition, HDAC11 has been reported to decrease histone acetylation at the promoter region of LKB1, thereby impeding its transcription and expression in HCC.^[^ ^33a^ ^]^ Therefore, we investigated the mRNA levels of LKB1, CaMKKβ, and TAK1 in FFA‐induced HepG2 cells treated with **B6** (Figure 11H–J). In cells treated with **B6**, there was an observed increase in LKB1 mRNA levels, which could be attributed to the inhibition of HDAC11 by **B6**, thereby diminishing its transcriptional repression of LKB1. However, the mRNA levels of CaMKKβ and TAK1 did not exhibit significant changes. We also examined the protein expression of LKB1 and p‐LKB1 and found that **B6** was able to increase LKB1 expression, accompanied by an elevation in its phosphorylation (Figure 11K). The therapeutic effect of B6 was abrogated following overexpression of HDAC11 (Figure 11L,M). In summary, **B6** exerts its effects through the inhibition of HDAC11, leading to the modulation of AMPKα1 Thr172 phosphorylation. This modulation is likely achieved by alleviating the transcriptional repression of LKB1 by HDAC11 (Figure 11N). Consequently, this molecular cascade results in a reduction of DNL and an enhancement of fatty acid oxidation.

### 2.9 **B6** Displays Favorable Safety Profile

Compound **B6** significantly mitigated the pathological symptoms in MASLD mice at dosages of 5 and 10 mg kg^−1^. To evaluate the in vivo safety profile and potential toxicity of compound **B6**, a subacute toxicity study was undertaken. In this study, ICR mice were administered an oral dose of 50 mg kg^−1^ daily for a duration of 30 d, a concentration tenfold higher than the effective dose. Throughout the experimental period, the overall health and behavior of the mice were meticulously monitored. Notably, there were no fatalities observed in any of the experimental groups, and all mice remained in good health, exhibiting no signs of toxicity. A comprehensive data analysis revealed that **B6** treatment did not induce any significant abnormal changes in the body weight of male and female mice when compared to the control group (Figure S6A, Supporting Information). In addition, histopathological evaluations of critical organs, such as the liver, kidneys, heart, spleen, and lungs, demonstrated no significant differences compared to the control group (Figure S6B–K, Supporting Information). Based on these findings, it can be reasonably concluded that compound **B6** exhibits favorable a safety profile, with a therapeutic window of at least tenfold.

## 3 Discussion

MASLD represents one of the most prevalent etiologies of liver disease, with its global incidence rising annually.^[^ ^36^ ^]^ As MASLD advances, it can evolve into MASH, potentially resulting in liver fibrosis, cirrhosis, and end‐stage liver disease.^[^ ^37^ ^]^ The pathogenesis of MASLD is notably complex, and a complete elucidation remains elusive. Current prevailing theories propose that the development of MASLD is influenced by a multifactorial interplay, including lipid intake, DNL, fatty acid oxidation, and lipid efflux.^[^ ^38^ ^]^ In the initial stage of MASLD, the condition is reversible, with dietary regulation and appropriate physical activity being the most effective interventions.^[^ ^39^ ^]^ However, maintaining a healthy lifestyle can be challenging, underscoring the urgent need for the development of therapeutic drugs for MASLD. At present, there remains a paucity of effective pharmacological treatments available for this condition.^[^ ^1a^ ^]^ In this study, we observed an upregulation of HDAC11 expression in the liver of HFD mice. Previous studies on HDAC11 in the context of metabolism has predominantly concentrated on obesity and thermogenesis within adipose tissue,^[^ ^16^ ^]^ with scant investigation into the association between HDAC11 and MASLD. To address this gap, we have developed a novel HDAC11‐specific inhibitor designated **B6**. This compound functions by inhibiting HDAC11, thereby activating AMPKα, which in turn reduces DNL and promotes fatty acid oxidation in liver.

HDACs are a class of enzymes responsible for catalyzing the deacetylation of lysine residues on both histone and nonhistone proteins.^[^ ^40^ ^]^ In humans, there are 18 recognized HDACs categorized into four classes based on distinctions in their structure, subcellular localization, and cofactor requirements. HDAC11 is the sole member of class IV HDAC. It is primarily active within the nucleus and shares structural similarities with class I and II HDACs.^[^ ^12^ ^]^ HDAC11 demonstrates both deacetylase and defatty‐acylase activities, with its defatty‐acylase activity being over 10 000 times more efficient than its deacetylase activity.^[^ ^14a^ ^]^ HDAC11 has been implicated in a variety of diseases, including cancer, inflammatory and immune disorders, cardiovascular diseases, and neurodegenerative conditions.^[^ ^33^, ^41^ ^]^ In recent years, there has been a growing interest among researchers in elucidating the metabolic functions of HDAC11. Studies have shown that HDAC11 deficiency stimulates lipolysis in mice, leading to thermogenesis, stabilization of blood glucose, and enhanced insulin sensitivity.^[^ ^15a^ ^]^ Furthermore, the absence of HDAC11 upregulates the expression of UCP1, thereby augmenting fat metabolism.^[^ ^16^ ^]^ Concurrently, HDAC11 deficiency activates AMPK, promoting fatty acid oxidation in skeletal muscles.^[^ ^33b^ ^]^ However, despite previous studies suggesting that HDAC11 deficiency may activate AMPKα, no one has explored a deeper relationship between HDAC11 and AMPKα. Consistent with these findings, our transcriptomic and proteomic analyses indicate significant enrichment of the AMPK signaling pathway in FFA‐treated HepG2 cells with HDAC11 knockdown. And the inhibition of HDAC11 by **B6** activates AMPKα both in vivo and in vitro. Furthermore, we have also demonstrated the druggability of HDAC11 as a therapeutic target for MASLD. According to previous studies, HDAC11 impedes acetylation of the LKB1 promoter region, which in turn inhibits LKB1 transcription.^[^ ^33a^ ^]^ The expression of LKB1 was indeed elevated after **B6** treatment, so we hypothesized that **B6** promotes the phosphorylation of AMPKα by inhibiting HDAC11 to promote LKB1 expression. Meanwhile, AMPKα plays a pivotal role in both DNL and fatty acid oxidation processes.^[^ ^42^ ^]^ Hence, our study has demonstrated that **B6** modulates the HDAC11/AMPKα axis to reduce DNL and enhance fatty acid oxidation, thereby partially ameliorating MASLD. Furthermore, we validated our findings by overexpressing HDAC11 or knocking down AMPKα in HepG2 cells (Figures 9 and 10).

Given the significant roles of HDAC11 in various biological processes, several HDAC11 inhibitors have been developed. Notable among these are Elevenostat (JB3‐22), SIS17, and FT895 (a hydroxamate‐based small molecule compound), which are specific inhibitors of HDAC11.^[^ ^43^ ^]^ Other HDAC11 inhibitors, including TD034 and PB94, exhibit strong inhibitory activity but require improvement in selectivity and druggability.^[^ ^17^ ^]^ Among the compounds studied, SIS17 is a hydrazide‐based HDAC11 inhibitor that potentially offers superior metabolic properties compared to the hydroximic inhibitors Elevenostat and FT895.^[^ ^19^, ^44^ ^]^ In contrast, **B6** demonstrates significantly higher HDAC11 inhibitory activity, with an *IC*_50_ value of 51.1 × 10^−9^ m. Given **B6**’s favorable pharmacokinetic profile and its preferential accumulation in the liver, we propose that **B6** will serve as an invaluable tool for future research on HDAC11 in the context of liver diseases.

Our study identifies HDAC11 as a promising therapeutic target for the treatment of MASLD and introduces **B6** as a novel, highly selective HDAC11 inhibitor. The findings indicate that **B6** effectively mitigates HFD‐induced MASLD through the activation of the HDAC11/AMPK axis. By inhibiting HDAC11, **B6** reduces DNL in the liver and enhances fatty acid oxidation, thereby ameliorating the pathological processes associated with MASLD. As demonstrated by the KEGG enrichment analysis (Figure 10A; Figure S5A, Supporting Information), although the AMPK signaling pathway is significantly enriched following intervention on HDAC11, several other relevant pathways, such as the Adipocytokine signaling pathway and the Insulin signaling pathway, also merit further investigation, as they play crucial roles in MASLD. In conclusion, this study provides a critical insight into the development of novel therapeutic approaches for MASLD and elucidate the molecular mechanisms underlying HDAC11‐mediated regulation of energy metabolism. It lays a promising groundwork for the future development of more precise and effective strategies for the treatment of MASLD.

## 4 Experimental Section

### Reagents and Antibodies

Alkynyl myristic acid (Alk14) (82909‐47‐5) and Biotin‐PEG4‐Amide‐C6‐Azide (1006592‐62‐6) were obtained from Leyan (Shanghai, China). Click‐iT Protein Reaction Buffer Kit (C10276) was provided by ThermoFisher (Carlsbad, USA). Streptavidin magnetic beads (HY‐K0208) was purchased from MedChemExpress (NJ, USA). Anti‐HDAC11 (A6140), anti‐ChREBP (A7630), anti‐PGC1α (Α12348), and anti‐PPARα (Α18252) were purchased from Abclonal (Wuhan, China). Anti‐HSP90 (13171‐1‐AP), anti‐SCD1 (28678‐1‐AP), anti‐CPT1A (15184‐1‐AP), anti‐α‐Tubulin (HRP‐66031), anti‐β‐actin (HRP‐60008), anti‐GAPDH (HRP‐60004), anti‐rabbit HRP (SA00001‐2), and anti‐mouse HRP(SA00001‐1) were purchased from Proteintech Group (Chicago, USA). Anti‐SHMT2 (DF6347), anti‐phospho‐AMPKα (Thr172) (AF3423), anti‐AMPKα (AF6423), and anti‐SREBP1c (AF6283) were purchased from Affinity Biosciences, Inc. (Cincinnati, USA). Anti‐FASN (sc‐48357), anti‐Ac‐HH3 (sc‐518011), anti‐Ac‐HH4 (sc‐377520), and anti‐Ac‐Tubulin (sc‐23950) were purchased from Santa Cruz Biotechnology, Inc. (Santa Cruz, USA).

### Cell Culture and Treatments

HepG2 cells, AML12 cells, and HEK293T cells were purchased from the American Type Culture Collection (ATCC, Manassas, USA). HepG2 cells and HEK293T cells were cultured in DMEM medium (KeyGEN, Nanjing, China) supplemented with 10% fetal bovine serum (FBS) and 100 U mL^−1^ of penicillin‐streptomycin. AML12 cells were cultured in a complete medium (CM‐0602, Procell Life Science & Technology Co., Ltd., China). All cells were placed at 37 °C in 95% humidity and 5% CO_2_ atmosphere.

For the lipid accumulation experiment, HepG2 cells were incubated for 24 h in DMEM containing 0.5 × 10^−3^ m of a mixture of free fatty acids (FFA; oleic acid: palmitic acid  =  2:1) and 1% FFA‐free bovine serum albumin to induce intracellular lipid accumulation, together with **B6** treatment.

For click chemistry experiment, HEK293T cells were first incubated with **B6** for 24 h, followed by treatment with Alk14 for 4 h prior to harvest.

### HDAC11 Inhibition Fluorescence Assay

The HDAC11 enzyme was a full‐length human recombinant HDAC enzyme sourced from BPS Bioscience. In detail, a total of 20 µL of recombinant HDAC11 enzyme solution (working concentration = 1 × 10^−9^ m) was combined with varying concentrations of the test compound (20 µL) in a 96‐well black plate. The mixture was then incubated at a temperature of 30 °C for a duration of 1 h. Subsequently, 10 µL of the specific fluorogenic substrate (ETDKacyl‐Kmyr) was added. Following 2 h incubation at 30 °C, the catalytic reaction was ceased by adding 10 µL of a developer solution consisting of 2 mg mL^−1^ trypsin and 5 × 10^−6^ m trichostatin A (TSA). After a standing period of 6 h, the fluorescence intensity was assessed using a microplate reader at excitation and emission wavelengths of 360 and 460 nm, respectively. The inhibition ratios were calculated based on the fluorescence intensity readings of the test wells compared to the control wells. GraphPad Prism 8.0 software was employed to generate *IC*_50_ curves and determine the corresponding *IC*_50_ values utilizing the “log(inhibitor) versus normalized response‐variable slope” function.

*In Vitro HDACs Inhibition Fluorescence Assay*: For the inhibitory activity against HDAC1‐10, enzymes were also procured from BPS Bioscience. The in vitro HDAC1‐9 inhibition assays were conducted following the established method described previously.^[^ ^45^ ^]^ The working concentrations of HDAC1‐10 were 4 × 10^−9^ m. The fluorogenic substrate of HDAC1, 2, 3, and 6 was Boc‐Lys (acetyl)‐AMC, and the substrate of HDAC4, 5, 7, 8, 9, and 10 was Boc‐Lys (trifluoroacetyl) AMC. The developer solution consists of 30 mg mL^−1^ trypsin and 5 × 10^−6^ m TSA. A standing period of 30 min was sufficient before assessing the fluorescence intensity.

For the inhibitory activity against SIRT2, substrate is the AcIQF.^[^ ^46^ ^]^ Dilution series compound was incubated with the SIRT2 enzyme (0.2 × 10^−3^ m) for 30 min at 25 °C. Then AcIQF (5 × 10^−3^ m) and NAD^+^ (200 × 10^−3^ m) were added to start the reaction at 25 °C. 2 h later, 50 mL stop solution (containing ≈2 U mL^−1^ trypsin and 4 × 10^−3^ m nicotinamide) was added to stop the reactions. Then the mixture was incubated for 30 min, and the fluorescence intensity was assessed.

### Click Chemistry Assay

After the treatment mentioned in Section 4.2, HepG2 cells were lysed in 1% SDS lysis buffer (50 × 10^−3^ m Tris‐HCl pH 8.0, 1% (w/v) SDS) with protease‐phosphatase inhibitor cocktail (ThermoFisher).^[^ ^47^ ^]^ Click chemistry reactions were performed using commercially available reagents as per manufacturer instructions. 100 µg labeled protein was incubated with 50 µL of streptavidin magnetic beads overnight at 4 °C with end‐over‐end rotation. Following incubation, the beads were collected and rinsed three times with washing buffer. After the final wash, the supernatant was discarded, sample buffer was added to the beads and boiled at 95 °C for 5 min. The samples were further analyzed via western blotting using antibody specific for detection of endogenous SHMT2.

### Oil Red O Staining of Intracellular Fat

Cells were fixed with 4% paraformaldehyde, followed by sequential staining with Oil Red O and hematoxylin.

Mito‐Tracker Deep Red FM staining: Cells were stained with 200 × 10^−9^ m Mito‐Tracker Deep Red FM staining solution provided by Beyotime Biotechnology (Shanghai, China) for 30 min.

### Fatty Acid Oxidation Measurement (Seahorse)

OCR of HepG2 cells was analyzed by XFp Extracellular Flux Analyzer (Seahorse Bioscience). Cells were washed with XF assay medium and placed into a 37 °C incubator without CO_2_ for 45 min to 1 h. For the mitochondrial stress test, the cells were treated with the ATP synthase inhibitor oligomycin (1.0 × 10^−6^ m), the chemical uncoupler FCCP (1.0 × 10^−6^ m), and the electron transport inhibitor antimycin A (0.5 × 10^−6^ m).

### Inhibition of HDAC11 Expression by siRNA

The siRNA for HDAC11 (sense, 5′‐CACACGAGGCGCUAUCUUATT‐3′; antisense, 5′‐UAAGAUAGCGCCUCGUGUGTT‐3′) was synthesized by Tsingke Biotech (Nanjing, China). HepG2 cells plated in six‐well plates were transfected with siRNA using Lipofectamine RNAiMAX Transfection Reagent (Thermo Fisher Scientific, Shanghai, China).

### Transcriptomic Analysis

HepG2 cells were transfected with siHDAC11 and incubated with FFA for 24 h. Subsequently, the cells were collected, washed with PBS, centrifuged to collect the cell pellet, and then rapidly frozen in liquid nitrogen. Total RNA was extracted using the TRIzol reagent according to the manufacturer's protocol. RNA purity and quantification were evaluated using the NanoDrop 2000 spectrophotometer (Thermo Scientific, USA). RNA integrity was assessed using the Agilent 2100 Bioanalyzer (Agilent Technologies, Santa Clara, CA). The transcriptomic library was constructed using the VAHTS Universal V6 RNA‐seq Library Prep Kit according to the manufacturer's instructions. RNA‐seq was performed by OE Biotech Co., Ltd. (Shanghai, China). To analyze the differential expression of genes, the limma package (version 3.26.8) was utilized in the R Bioconductor project. Genes showing significant differential expression were identified based on criteria of |fold change| > 1 and a *p*‐value < 0.05. The Kyoto Encyclopedia of Genes and Genomes was used for pathway analysis. To conduct gene set enrichment analysis, the ClusterProfiler package for clustering analysis, the MSigDB package to acquire reference gene sets, and the MSigDB Collections database for gene set analysis were utilized.

### Proteomic Analysis

HepG2 cells were transfected with siHDAC11 and incubated with FFA for 24 h. Subsequently, the cells were collected, washed with PBS, centrifuged to collect the cell pellet, and then rapidly frozen in liquid nitrogen. Then, a proteomic analysis was conducted by the Majorbio Proteomic Service (Shanghai, China). The proteins were extracted and the concentration was determined using the BCA method. Then SDS‐PAGE was carried out. The proteins were treated with Triethylammonium bicarbonate buffer and digested with trypsin overnight. Peptides were desalted and quantified using a Peptide Quantification Kit (Thermo Fisher Scientific, USA). Peptides were desalted and quantified, followed by analysis using mass spectrometry for identification. The raw data were analyzed with Spectronaut software. To analyze the differential expression of genes, the limma package (version 3.26.8) was utilized in the R Bioconductor project. Genes showing significant differential expression were identified based on criteria of |fold change| > 1 and a *p*‐value < 0.05. The Kyoto Encyclopedia of Genes and Genomes was used for pathway analysis. To conduct gene set enrichment analysis, the ClusterProfiler package for clustering analysis, the MSigDB package to acquire reference gene sets, and the MSigDB Collections database for gene set analysis were utilized.

### Preparation of HDAC11, AMPKα1, and AMPKα1 T172A Plasmid Vector

Human HDAC11 cDNA (NM_024827.4) and human AMPKα1 cDNA (NM_001355028.2) were cloned into pEGFP‐C2 vector by Tsingke Biotech (Nanjing, China). The hHDAC11 expression vector was named as pEGFP‐C2‐hHDAC11, and the AMPKα1 expression vector was named as AMPKα1‐WT. The dominant negative mutant of AMPKα1 was created by mutating the Thr172 residue into Ala, named AMPKα1‐T172A.

Cells were seeded into six‐well plates. When cell‐density reached 70%–80%, cells were switched to fresh medium, and transfected with plasmid using liposomal transfection reagent from Yeasen Biotechnology (Shanghai, China).

### Generation of a CRISPR‐Cas9‐Mediated AMPKα1 knockout HepG2 Cell Line

An AMPKα1 genome knockout HepG2 cell line was generated using the clustered regulatory interspaced short palindromic repeat (CRISPR)‐associated 9 (Cas9) technology as reference.^[^ ^48^ ^]^ Guide RNA was designed to target exon 2 of AMPKα1 by an online CRISPR Design Tool (http://tools.genome‐engineering.org). The sequences of guide oligos are: top, 5′‐CACCGTTGGCAAACATGAATTGAC‐3′; and bottom, 5′‐AAACGTCAATTCATGTTTGCCAAC‐3′. The underlined letters represent the restriction site of BbsI. After annealed, the oligo duplex was ligated into pSpCas9 (BB)‐2A‐Puro vector (Addgene plasmid ID: 48139, pre‐digested with BbsI) to generate Cas9‐AMPKα1. HepG2 cells were then transfected with plasmid DNA for Cas9‐AMPKα1 and pSpCas9 vector, respectively. The transfected cells were plated in 96‐well plates with the limiting dilution and cultured in the medium containing puromycin (3 µg mL^−1^) for screening AMPKα1 knockout. After 3 weeks of the first round selection, the formed mono clones were subjected to the second round selection. The mutation was confirmed by both PCR and DNA sequencing, and lack of AMPKα protein expression was determined by Western blot. The cells lacking of AMPKα1 expression and the corresponding control cells were defined as CRISPR‐AMPKα1 cells and CRISPR‐Ctrl cells, respectively.

### Experimental Animals and Design

Pharmacokinetics study was performed by Medicilon Preclinical Research (Shanghai) LLC. Male ICR mice, which were purchased from Sino‐British SIPPR Lab Animal Ltd., Shanghai (25–30 g, 14 weeks of age), were fasted overnight before administration and resumed food 6 h after initial plasma sampling. Compound was dissolved in solution containing 10% DMAC, 40% PEG‐400, and 50% water and was delivered to three animals by intravenous bolus injection (i.v.) and oral gavage (p.o.) at a dose of 5 and 20 mg kg^−1^ with a volume of 5 and 10 mL kg^−1^, respectively. Blood samples were collected at the following time points: 5, 15, 30 min and 1, 2, 4, 6, 8, and 24 h post dose administration. The blood samples were taken via submandibular vein, 30 µL/time point.

Sample were placed in tubes containing K2‐EDTA and stored on ice until centrifuged. The blood samples were centrifuged at 6800 *g* for 6 min at 2–8 °C within 1 h after collected and stored frozen at ≈−80 °C. A 20 µL plasma sample was treated with 400 µL of methanol containing 10 ng mL^−1^ IS to precipitate the protein. The mix was then vortexed for a minute and centrifuged at 18 000 *g* for 7 min. The resulting supernatant (400 µL) was transferred to a 96‐well plate, and an injection of 1 µL of this supernatant was used for the LC‐MS/MS analysis. The animal protocols used in this study were approved by the Preclinical R&D Unit of Medicilon Institutional Animal Care and Use Committee (IACUC) (26030‐21002) and operated in accordance with the Guide for the Care and Use of Laboratory Animals published by the National Institutes of Health (NIH).

For the distribution experiment, compound was dissolved in solution containing 10% DMAC, 40% PEG‐400, and 50% water and was delivered to three male ICR mice by intraperitoneal injection (i.p.) at a dose of 5 mg kg^−1^. Then three rats were sacrificed for each 0.17, 0.5, and 1 h time period. Subsequently, the heart, liver, spleen, lung, and kidney were immediately collected. Blood samples were obtained via cardiac puncture and processed by centrifugation to retrieve the plasma fraction. Tissues were flushed with normal saline, dried, weighed, and then homogenized in 5 mL methanol. Tissue samples were centrifuged at 4000 *g*, and supernatants were stored in freezer at −80 °C before analyzing. 20 µL of the reconstitution solution were analyzed via LC‐MS system. The 8‐week‐old male C57BL/6J mice were provided by GemPharmatech (Nanjing, China).

For pharmacology experiments, mice were divided into five groups (*n*  =  8 per group): control group (C), **B6** 10 mg kg^−1^ group [B6 (10)], high fat diet group (H), HFD + 5 mg kg^−1^ **B6** group [HB6 (5)], and HFD + 10 mg kg^−1^ **B6** group [HB6 (10). The 60 kcal% fat diet (MD12033) used for establishing the MASLD model was purchased from Medicience (Yangzhou, China). The C and B6 (10) groups were fed a chow diet for 12 weeks, followed by gavage administration of vehicle and 10 mg kg^−1^ **B6** for the subsequent four weeks, respectively. The H, HB6 (5) and HB6 (10) groups were fed a 60 kcal% fat diet for 12 weeks, followed by gavage administration of vehicle, 5 mg kg^−1^ **B6** and 10 mg kg^−1^ **B6** for the subsequent four weeks, respectively.

For evaluation of subacute toxicity, ICR mice were divided into two groups (*n* = 8 per group (4M, 4F)). The **B6** was prepared in vehicle (10% DMSO and 90% 0.5% HPMC aqueous solution) to administer once daily, by oral gavage for 30 d in the treatment groups. To the control groups, vehicle was administered orally. The animals were monitored for clinical and behavioral symptoms such as body weight, diarrhea, immobility, and mortality during the first day and thereafter for 30 d.

The animal protocols for distribution, pharmacology, and subacute toxicity experiments in this study were approved by the Ethics Committee of Hefei University of Technology (HFUT20230427010) and operated in accordance with the Guide for the Care and Use of Laboratory Animals published by the NIH.

### TG, FFA, and TC Analysis

TG, FFA, and TC kits were purchased from Solarbio (Beijing, China). The liver tissues from mice were homogenized and processed according to the instructions.

### Histopathological Analysis

For hematoxylin and eosin (H&E) staining, liver tissues were fixed in 4% paraformaldehyde, dehydrated, and then embedded in paraffin. Sections were cut into 5‐µm‐thick slices, pasted on glass slides.

For Oil Red O staining, liver tissues were fixed in 4% paraformaldehyde, dehydrated, and then embedded in OCT. Sections were cut into 5‐µm‐thick slices, pasted on glass slides.

### Biochemical Analysis

ALT, AST, ALP, TG, NEFA, CHO, LDL‐C, and HDL‐C were assessed using a Hitachi 7020 automatic analyzer (Hitachi, Tokyo, Japan).

### Glucose Tolerance Test (GTT) and Insulin Tolerance Test (ITT)

After 12 weeks on the feeding regimen, mice were fasted in paper bedding for 16 or 3 h for glucose or insulin tolerance tests, respectively. Glucose (1 g kg^−1^ body weight) and insulin (0.5 U kg^−1^ body weight) were administered separately through oral gavage and intraperitoneal injection. Blood glucose level was measured using glucose meter prior to injection and several times after injection as indicated.

### Metabolic Measurements in Mice

Food intake, locomotor VO_2_, VCO_2_, and heat production were measured by the TSE system, and RER were calculated using the manufacturer's system software.

### Western Blot

Western blot was carried out as described.^[^ ^49^ ^]^ In brief, samples were lysed with a lysis buffer. The same quantity of total proteins (40–60 µg) from each sample was separated on SDS‐PAGE followed by transfer onto a nitrocellulose filter membrane and incubation with the indicated antibodies. After incubation with secondary antibodies, the protein bands were visualized using a chemiluminescence imaging system. All samples in the same group were processed simultaneously.

### Quantitative Real‐Time PCR (qRT‐PCR)

Cells and liver tissues were extracted for total RNA. A reverse transcription kit was used to synthesize cDNA. qRT‐PCR was performed with the primers listed in Table S1 (Supporting Information) using the SYBR green PCR master mix. The coding sequence of the target gene was found in NCBI, and Primer3 (v.0.4.0) was used to design amplification primers. The results were normalized by β‐actin in the corresponding sample.

### Chemical Synthesis

The primary synthetic data are available in the Supporting Information.

### Molecular Docking

The structure of human HDAC11 was obtained from AlphaFold Protein Structure Database (https://alphafold.com). The docking site was defined using the findbox plugin in PyMOL. Substrates were docked into the catalytic domain of HDAC11 by LeDock (http://lephar.com), which performs an exhaustive search of the orientation, position, and conformation of a ligand at the binding site.

### Statistical Analysis

The data analysis was conducted by a technician who was blind to the experimental design. All data confirmed a normal distribution. Biological experimental replicates in each group are shown in the figure legends. Experiments were repeated at least three times. Statistical analysis was performed using GraphPad Prism 9.0 software. Two‐tailed Student's *t*‐tests were used for comparisons between two groups, while one‐way ANOVA with a Tukey post‐hoc test was used for comparisons among more than two groups. A significant difference was considered if *p* < 0.05.

## Conflict of Interest

The authors declare no conflict of interest.

## Supporting information



Supporting Information

## Data Availability

The data that support the findings of this study are available from the corresponding author upon reasonable request.
