# Supplementary material for: Targeting Histone Deacetylase 11 with a Highly Selective Inhibitor for the Treatment of MASLD
Source: Adv Sci (Weinh). 2025 Feb 20;12(15):2412903. doi: 10.1002/advs.202412903 (PMC12005767; doi:10.1002/advs.202412903)
Supplement: Supplementary file 1 — Supporting Information [file ADVS-12-2412903-s001.docx]

Supporting Information

**Targeting Histone Deacetylase 11 with a Highly Selective Inhibitor for the Treatment of MASLD**

*Feng Zhang, Kairui Yue, Simin Sun, Shengyuan Lu, Geng Jia, Yang Zha, Shuang Zhang, C. James Chou, Chenzhong Liao, Xiaoyang Li*, Yajun Duan**

Inventory

Supplementary Figures S1-S5

Supplementary Table 1

Supplementary Methods

NMR and HPLC spectrum of target compounds

Supplementary References

**
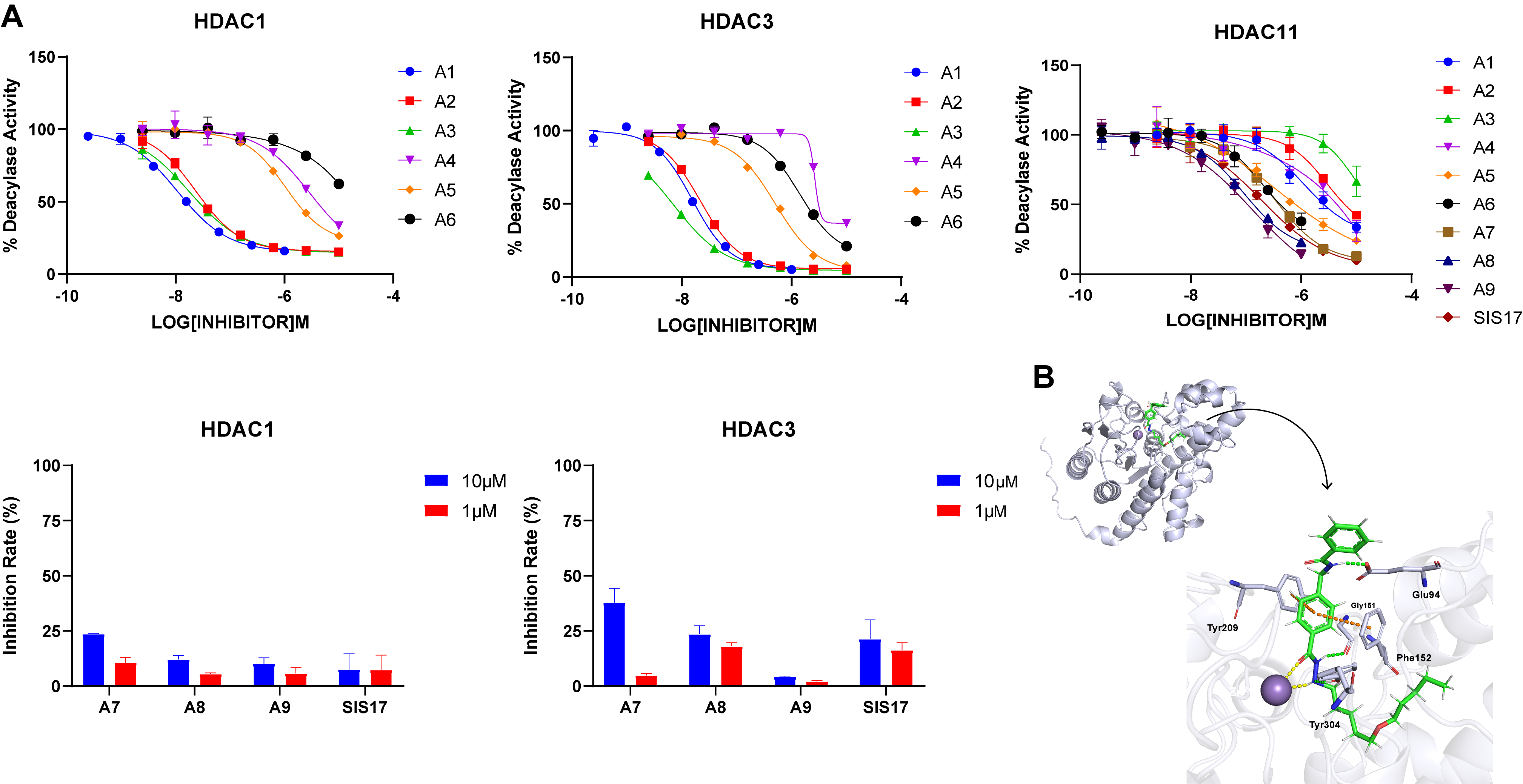
**

**Figure S1**. (A) IC_50_ curves of compound **A1**-**A6** against HDAC1, 3, and 11. The inhibition rate of compound **A7**-**A9** and SIS17 against HDAC1 and 3, the values were obtained from two independent experiments. (B) Docking pose of B6 (green) in the catalytic site of HDAC11. The available structures of HDAC11 were obtained from the AlphaFold Protein Structure Database (https://alphafold.com). Data are shown as mean ± SEM.


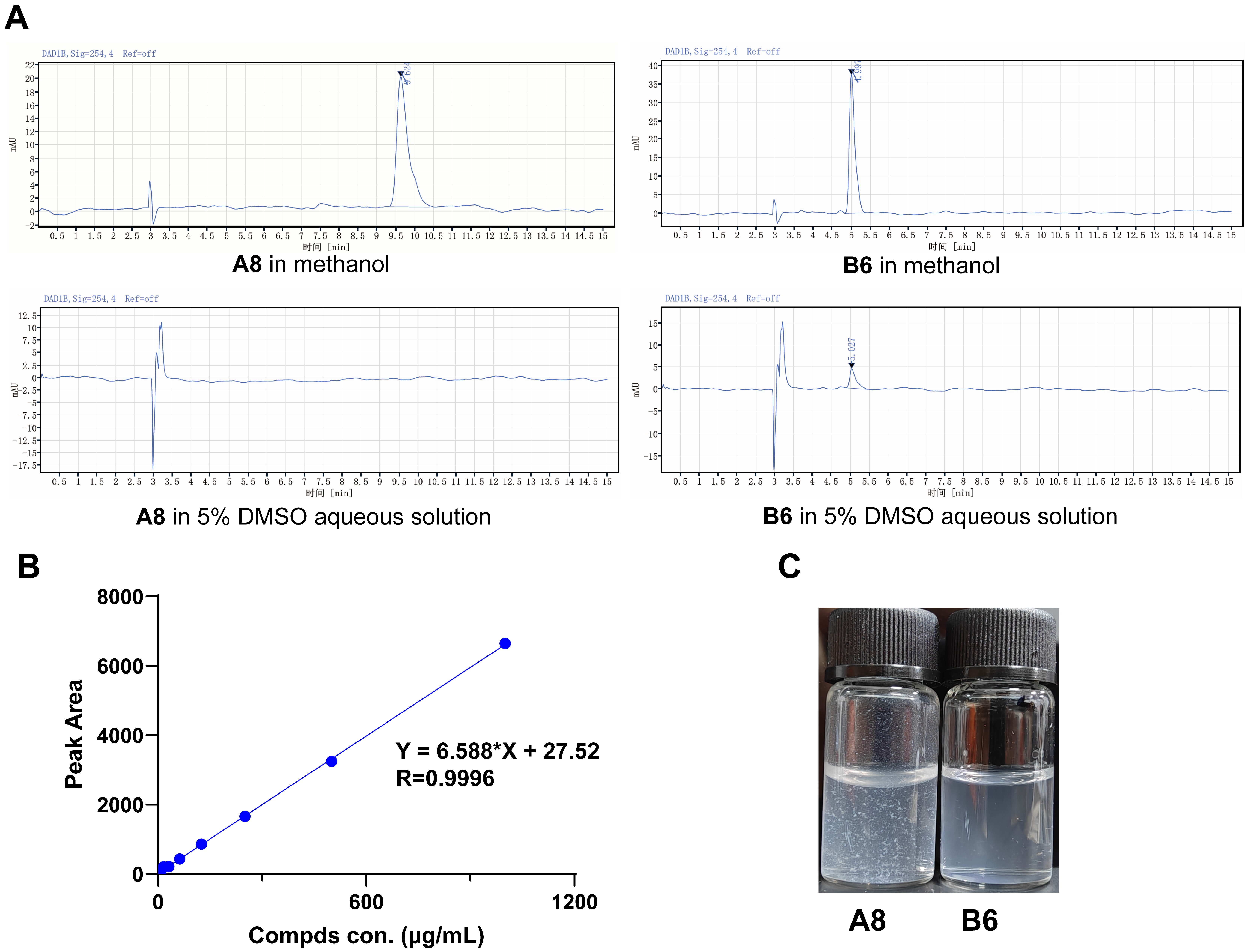


**Figure S2**. (A) Liquid chromatograms of compounds **A8** and **B6** in methanol and 5% DMSO aqueous solution, respectively. The chromatograms were determined with an Agilent 1260 HPLC, using an Agilent 5 TC-C18 column (5 μm, 250mm× 4.6 mm). The eluent system was made up of solvent A (water) and solvent B (methanol). Elution conditions: phase B was 85% The peaks were detected at 254 nm at a flow rate was 1.0 mL/min. (B) The peak areas of serially diluted compound **B6** were determined under the above-mentioned liquid chromatographic conditions, and a linear regression equation was fitted. (C) The dissolution status/solubility condition of compounds **A8** and **B6** in a 5% DMSO aqueous solution.


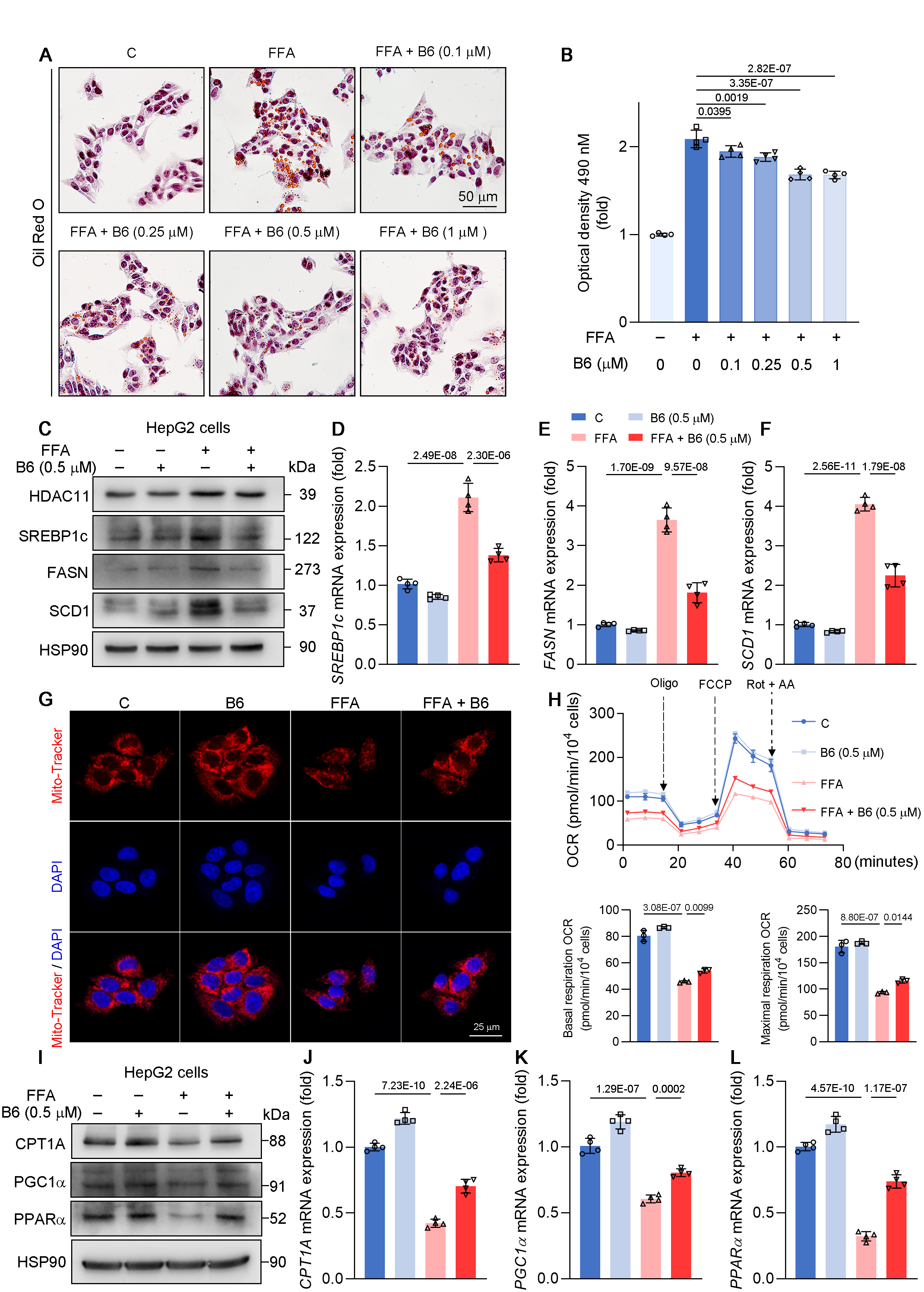


**Figure S3**. Effects of **B6** on de novo lipogenesis and fatty acid oxidation in HepG2 cells. (A) and (B) Oil Red O staining and optical density of HepG2 cells treated with **B6** of different concentration for 24h (n = 4). (C) Protein expression of HDAC11, SREBP1c, FASN, SCD1 and HSP90 of HepG2 cells treated with FFA and 0.5 μM **B6** for 24h (n = 3). (D-F) Relative normalized mRNA expression of SREBP1c, FASN, and SCD1 of indicated cells (n = 4). mRNA level of β-actin was used as normalized control. (G) Mito-Tracker Deep Red FM staining of indicated cells (n = 3). (H) Mitochondrial oxygen consumption rate in indicated cells (n = 3). (I) Protein expression of CPT1A, PGC1α, PPARα, and HSP90 in indicated cells (n = 3). (J-L) Relative normalized mRNA expression of CPT1A, PGC1α, and PPARα of indicated cells (n = 4). mRNA level of β-actin was used as normalized control. All experiments were repeated at least three times. Data are shown as mean ± SD. The *p* values were calculated by one-way ANOVAs.


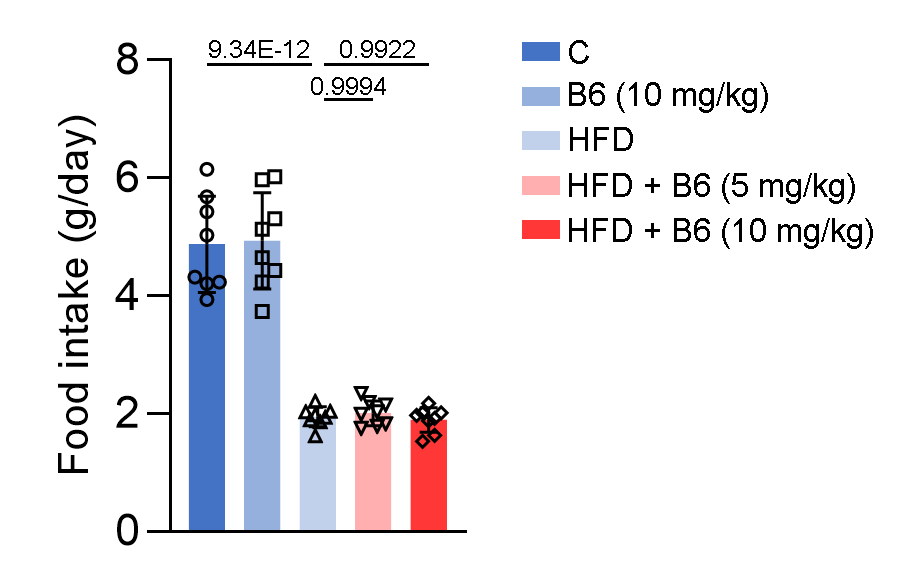


**Figure S4**. **B6** has no effect on food intake of mice (n = 8). Data are shown as mean ± SD. The *p* values were calculated by one-way ANOVAs.


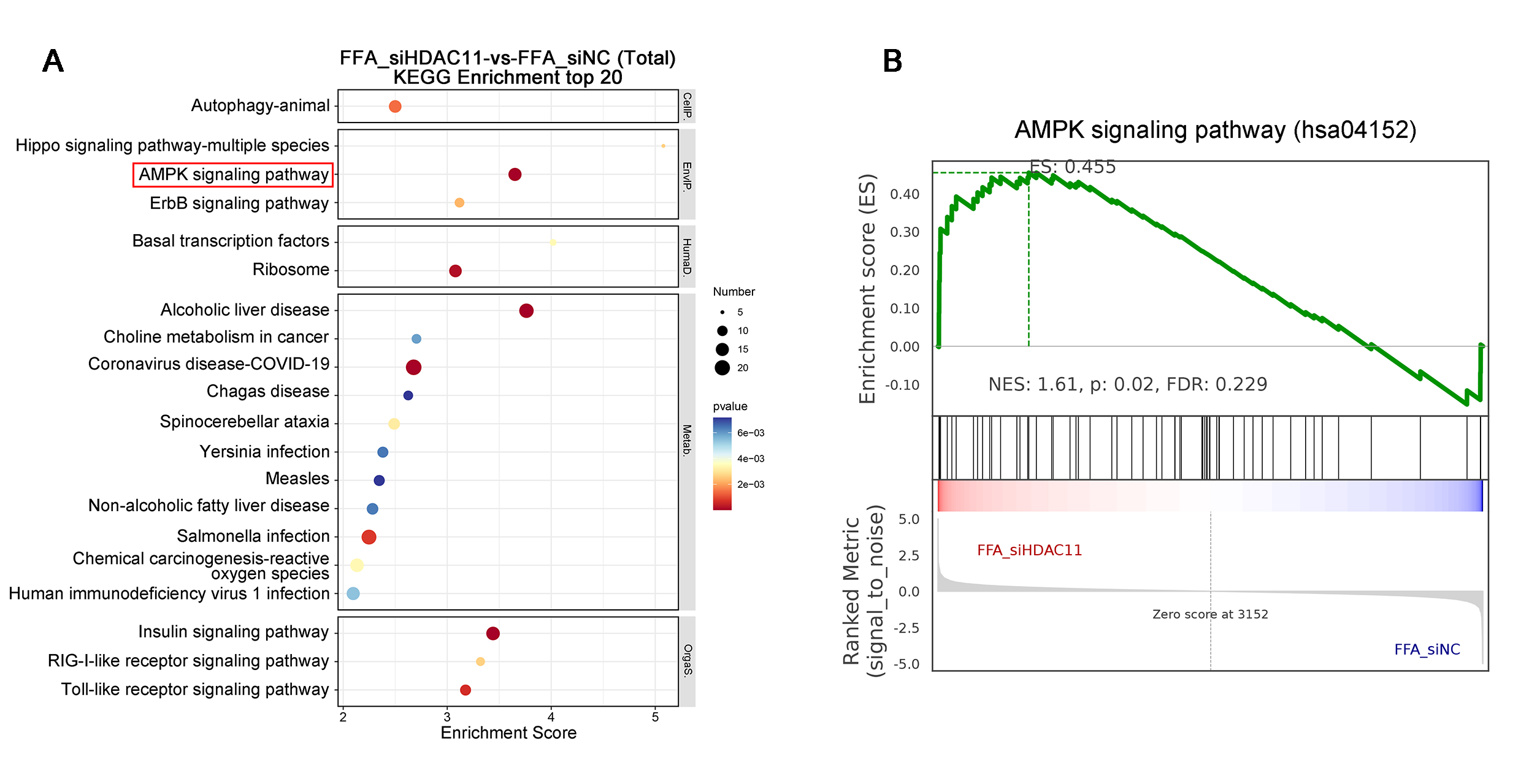


**Figure S5**. Proteomic data. (A) Top 20 of total differential gene KEGG enrichment pathway between the FFA_siHDAC11 and FFA_siNC groups of HepG2 cells (n = 3). (B) GSEA enrichment analysis graphs of AMPKα signaling pathway between the FFA_siHDAC11 and FFA_siNC groups of HepG2 cells (n = 3).


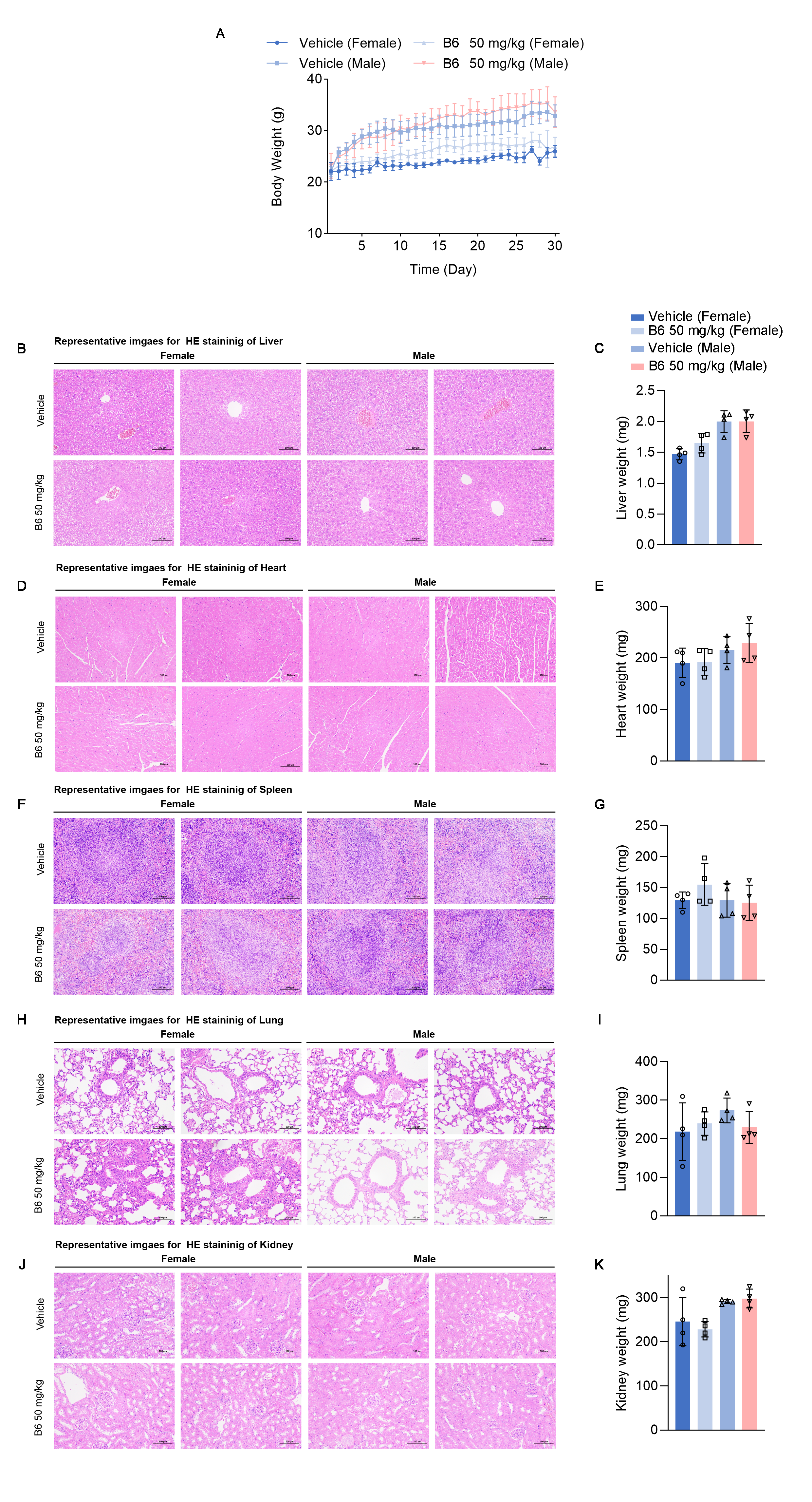
 **Figure S6**. (A) Body weight of male and female mice with continuous B6 dosing for 30 days (p.o. at 50 mg/kg) (n = 4). H&E staining and weight of (B and C) liver, (D and E) heart, (F and G) spleen, (H and I) lung, and (J and K) kidney of mice with continuous B6 dosing for 30 days (p.o. at 50 mg/kg) (n = 4). Data are shown as mean ± SD. The *p* values were calculated by one-way ANOVAs.

**Table S1.** Sequences of qRT-PCR primers.

| Gene | Forward | Backward |
| --- | --- | --- |
| h-FASN | CTGGCTCAGCACCTCTATCC | CAGGTTGTCCCTGTGATCCT |
| m-FASN | TCTGTGCCCGTCGTCTATAC | GGAGGTATGCTCGCTTCTCT |
| h-SREBP1c | GGGGACAAGGAATTCTCGGA | TCACCACAGCTGTCAGAGAG |
| m-SREBP1c | GGATTGCACTTTCGAAGACATG | AGCATAGGGTGGGTCAAATAGG |
| h-SCD1 | TGCCCACCACAAGTTTTCAG | CATCAGCAAGCCAGGTTTGT |
| m-SCD1 | CCCAGCTGTCAAAGAGAAGG | CAAGAAAGTGGCAACGAACA |
| h-CPT1A | CAAGGACATGGGCAAGTTTT | ATGCTTCTCAGACGCCAACT |
| m-CPT1A | CAAACCCACCAGGCTACAGT | TCCTTGTAATGTGCGAGCTG |
| h-PGC1α | CTGCTAGCAAGTTTGCCTCA | GCTTTCTGGGTGGACTCAAG |
| m-PGC1α | CCCTGCCATTGTTAAGACC | TGCTGCTGTTCCTGTTTTC |
| h-PPARα | AGAGTGGGCTTTCCGTGTC | GCCGCCTTCAGGTACAGTAG |
| m-PPARα | AGTTCGGGAACAAGACGTTG | CAGTGGGGAGAGAGGACAGA |
| h-LKB1 | CTCTTACGGCAAGGTGAAGG | TTTTGTGCCGTAACCTCCTC |
| h-CaMKKβ | TCAAACCTTCCAACCTCCTG | AGAAGATCTTGCGGGTCTCA |
| h-TAK1 | GGTGGAAGAGGTTGTTGGAA | AGAGCCCCCTTCAGCATATT |
| h-β-actin-F | AGAAAATCTGGCACCACACC | AGAGGCGTACAGGGATAGCA |
| m-β-actin | AGCCATGTACGTAGCCATCC | TCTCAGCTGTGGTGGTGAAG |

**Supplementary Methods**:

1. Synthesis procedure of **A1**-**A9** and **B1**-**B8**.

Synthetic routes of all of the compounds are outlined in Schemes 1 and 2.

The synthetic routes to key intermediate **4a**-**4h** were shown in Scheme 1. Firstly, different brominated fatty acids **1a**-**1e** react with corresponding fatty alcohols in the presence of KOH to get intermediates **2a**-**2h**. Then **2a**-**2h** are condensed with *N*,*O*-dimethylhydroxylamine hydrochloride in the presence of EDCI to obtain Weinreb amide **3a**-**3h**. Weinreb amides are reduced to aldehydes **4a**-**4h** by lithium aluminum hydride.

**Scheme 1**. Synthesis of compound **4a**-**4h**. *^a^*

*^a^*Reagents and conditions. (a) KOH, fatty alcohol, 90 ^o^C, 12 h; (b) N,O-dimethylhydroxylamine hydrochloride, EDCI, HOBt, TEA, DCM, 0 ^o^C-rt, 12 h; (c) LiAlH_4_, THF, -20 ^o^C, 2 h

The synthetic routes to compound **A1**-**A9** and **B1**-**B8** were shown in Scheme 2. Benzoic acid (**5**) and methyl 4-(aminomethyl)benzoate hydrochloride undergo condensation in the presence of TBTU to form compound **6**. Subsequently, compound **6** react with hydrazine hydrate to obtain hydrazide compound **7**. Finally, **7** react with corresponding aldehyde to get the Schiff base, then treated with NaBH_3_CN to afford compound **A1**-**A9** and **B1**-**B8**.

**Scheme 2**. Synthesis of compound **A1**-**A9** and **B1**-**B8**.*^a^*

Reagents and conditions: (a) methyl 4-(aminomethyl)benzoate hydrochloride, TBTU, TEA, DCM, 0 ^o^C-rt, 8 h; (b) N_2_H_4_·H_2_O, MeOH, reflux, 12 h; (c) fatty aldehyde or **4a-4h**, NaBH_3_CN, conc. HCl, MeOH, rt, 12 h;

Unless otherwise noted, all raw materials reagents, and solvents were used without further purification. ^1^H NMR spectra and ^13^C NMR spectra were obtained from JOE 400YH 400 MHz and Agilent Pro Plus 500 MHz instruments with TMS as an internal standard, δ in parts per million (ppm). Using the automated CombiFlash Rf system (Combiflash NextGen 100, Teledyne ISCO) and silica gel of 200-300 mesh to carry out flash chromatography. High-resolution mass spectrometry were gathered by Marine Biomedical Research Institute of Qindao, China. Using the HPLC (Agilent 1260 Infinity, Agilent) to determine all the tested compounds with purity of >95%, and the column was an Agilent 5 TC-C18 column (5 μm, 250 mm × 4.6 mm). The UV detection was performed at 254 nm and a flow rate was 1.0 mL/min. The temperature of the column was 25 °C, and quantity of injection was 5 μL. Mobile phase A: water; Mobile phase B: methanol. Gradient conditions: 0-10 min, phase B increased from 40% to 80%; 10-20 min, phase B increased from 80% to 90%; 20-30 min, phase B increased from 90% to 100% and maintained for 5 min; 35-40 min, phase B decreased from 100% to 40% and remained for 5 min.

**9-Methoxynonanoic acid (2b)**. KOH (700 mg, 12.6 mmol) was dissolved in dry MeOH (20 mL), then the solution was stirred at 90 ^o^C for 1 h. 9-bromononanoic acid (1 g, 4.2 mmol) was added to the solution in portions during a period of 15 min while maintaining the temperature at 90 ^o^C. The reaction was allowed to stir overnight. Solvent was evaporated under vacuum after the reaction completed. The residue was acidified with 1 N HCl to pH 4, then extred with EA (20 mL× 3), dried over MgSO_4_, and evaporated under vacuum to get the compound **2b**, a white solid (654 mg, 83%). The crude material was used directly for the next step without further purification.

**8-Ethoxyoctanoic acid (2c)**. Following the procedure of compound **2b**, compound **2c** was synthetized from 8-bromooctanoic acid (**1b**) and ethanol in 87% yield as a white solid.

**7-Propoxyheptanoic acid (2d)**. Following the procedure of compound **2b**, compound **2d** was synthetized from 7-bromoheptanoic acid (**1c**) and *n*-propanol in 78% yield as a light yellow solid.

**6-Butoxyhexanoic acid (2e)**. Following the procedure of compound **2b**, compound **2e** was synthetized from 6-bromohexanoic acid (**1d**) and 1-butanol in 64% yield as a light-yellow oil.

**5-(Pentyloxy)pentanoic acid (2f)**. Following the procedure of compound **2b**, compound **2f** was synthetized from 5-bromopentanoic acid (**1e**) and 1-pentanol in 67% yield as a brown oil.

**6-(2-Methoxyethoxy)hexanoic acid (2g)**. Following the procedure of compound **2b**, compound **2g** was synthetized from 6-bromohexanoic acid (**1d**) and 2-methoxyethanol in 54% yield as a brown oil.

**5-(2-Ethoxyethoxy)pentanoic acid (2h)**. Following the procedure of compound **2b**, compound **2h** was synthetized from 5-bromopentanoic acid (**1e**) and 2-ethoxyethanol in 51% yield as a brown oil.

**10-Hydroxy-*N*-methoxy-*N*-methyldecanamide (3a)**. To a solution of **2a** (500 mg, 2.66 mmol) in dry DCM (15 mL) were added 1-ethyl-3-(3-dimethylaminopropyl)carbodiimide hydrochloride (EDCI, 612 mg, 3.2 mmol), HOBt (432 mg, 3.2 mmol) and TEA (807 mg, 8 mmol) at 0 ^o^C. 30 min later, *N*,*O*-dimethylhydroxylamine hydrochloride (312 mg, 3.2 mmol) was added to the mixture. The reaction was stirred at room temperature overnight. The mixture was washed with water (3 × 30 mL), and brine (2 × 30 mL), and dried over Na_2_SO_4_. Volatiles were removed under vacuum to get the crude residue which was purified column chromatography to afford **3a**, a colorless oil (342 mg, 56%). ^1^H NMR (400 MHz, DMSO-*d*_6_) δ 4.30 (t, *J* = 5.2 Hz, 1H), 3.60 (s, 3H), 3.36-3.32 (m, 2H), 3.03 (s, 3H), 2.30 (t, *J* = 7.4 Hz, 2H), 1.51-1.30 (m, 4H), 1.24-1.16 (m, 10H).

***N*,9-Dimethoxy-*N*-methylnonanamide** **(3b)**. Following the procedure of compound **3a**, compound **3b** was synthetized from compound **2b** in 48% yield as a colorless oil.

^1^H NMR (400 MHz, Chloroform-*d*) δ 3.66 (s, 3H), 3.34 (t, *J* = 6.6 Hz, 2H), 3.31 (s, 3H), 3.16 (s, 3H), 2.39 (t, *J* = 7.6 Hz, 2H), 1.66-1.49 (m, 6H), 1.31-1.28 (m, 6H).

**8-Ethoxy-*N*-methoxy-*N*-methyloctanamide (3c)**. Following the procedure of compound **3a**, compound **3c** was synthetized from compound **2c** in 51% yield as a colorless oil. ^1^H NMR (400 MHz, Chloroform-*d*) δ 3.66 (s, 3H), 3.44 (q, *J* = 7.0 Hz, 2H), 3.38 (t, *J* = 6.7 Hz, 2H), 3.16 (s, 3H), 2.39 (t, *J* = 7.6 Hz, 2H), 1.65-1.51 (m, 4H), 1.35-1.29 (m, 6H), 1.17 (t, *J* = 7.0 Hz, 3H).

***N*-Methoxy-*N*-methyl-7-propoxyheptanamide (3d)**. Following the procedure of compound **3a**, compound **3d** was synthetized from compound **2d** in 42% yield as a colorless oil. ^1^H NMR (400 MHz, Chloroform-*d*) δ 3.66 (s, 3H), 3.38 (t, *J* = 6.7 Hz, 2H), 3.34 (t, *J* = 6.8 Hz, 2H), 3.16 (s, 3H), 2.39 (t, *J* = 7.6 Hz, 2H), 1.66-1.50 (m, 6H), 1.39-1.30 (m, 4H), 0.89 (t, *J* = 7.4 Hz, 3H).

**6-Butoxy-*N*-methoxy-*N*-methylhexanamide (3e)**. Following the procedure of compound **3a**, compound **3e** was synthetized from compound **2e** in 45% yield as a light yellow oil. ^1^H NMR (400 MHz, Chloroform-*d*) δ 3.65 (s, 3H), 3.38 (td, *J* = 6.6, 2.6 Hz, 4H), 3.15 (s, 3H), 2.40 (t, *J* = 7.6 Hz, 2H), 1.66-1.48 (m, 6H), 1.42-1.30 (m, 4H), 0.89 (t, *J* = 7.4 Hz, 3H).

***N*-Methoxy-*N*-methyl-5-(pentyloxy)pentanamide (3f)**. Following the procedure of compound **3a**, compound **3f** was synthetized from compound **2f** in 45% yield as a light-yellow oil. ^1^H NMR (400 MHz, Chloroform-*d*) δ 3.66 (s, 3H), 3.39 (dt, *J* = 13.8, 6.6 Hz, 4H), 3.16 (s, 3H), 2.43 (t, *J* = 7.4 Hz, 2H), 1.68-1.52 (m, 6H), 1.33-1.27 (m, 4H), 0.89-0.85 (m, 3H).

***N*-Methoxy-6-(2-methoxyethoxy)-*N*-methylhexanamide (3g)**. Following the procedure of compound **3a**, compound **3g** was synthetized from compound **2g** in 41% yield as a light-yellow oil. ^1^H NMR (400 MHz, Chloroform-*d*) δ 3.66 (s, 3H), 3.59-3.48 (m, 4H), 3.45 (t, *J* = 6.7 Hz, 2H), 3.37 (d, *J* = 0.7 Hz, 3H), 3.15 (s, 3H), 2.40 (t, *J* = 7.6 Hz, 2H), 1.67-1.56 (m, 4H), 1.38 (t, *J* = 7.8 Hz, 2H).

**5-(2-Ethoxyethoxy)-*N*-methoxy-*N*-methylpentanamide (3h)**. Following the procedure of compound **3a**, compound **3h** was synthetized from compound **2h** in 35% yield as a yellow oil. ^1^H NMR (400 MHz, Chloroform-*d*) δ 3.66 (s, 3H), 3.56 (s, 4H), 3.55-3.45 (m, 4H), 3.16 (s, 3H), 2.43 (t, *J* = 7.1 Hz, 2H), 1.68-1.64 (m, 4H), 1.20 (t, *J* = 7.0 Hz, 3H).

**10-Hydroxydecanal (4a)**. Lithium aluminum hydride (76 mg, 2 mmol) was added to a solution of compound **3a** (231 mg, 1 mmol) in dry THF. The reaction was stirred under a N_2_ atmosphere at -20 ºC for 2 h. The reaction was quenched with 1 N HCl at -20 ^o^C, then extracted by DCM (30 mL×3). The organic phase dried over Na_2_SO_4_. Volatiles were removed under vacuum to get the crude residue **4a**, a colorless oil (123 mg, 71%). The crude material was used directly for the next step without further purification.

**9-Methoxynonanal (4b)**. Following the procedure of compound **4a**, compound **4b** was synthetized from compound **3b** in 42% yield as a colorless oil.

**8-Ethoxyoctanal (4c)**. Following the procedure of compound **4a**, compound **4c** was synthetized from compound **3c** in 67% yield as a colorless oil.

**7-Propoxyheptanal (4d)**. Following the procedure of compound **4a**, compound **4d** was synthetized from compound **3d** in 72% yield as a colorless oil.

**6-Butoxyhexanal (4e)**. Following the procedure of compound **4a**, compound **4e** was synthetized from compound **3e** in 65% yield as a light-yellow oil.

**5-(Pentyloxy)pentanal (4f)**. Following the procedure of compound **4a**, compound **4f** was synthetized from compound **3f** in 68% yield as a brown oil.

**6-(2-Methoxyethoxy)hexanal (4g)**. Following the procedure of compound **4a**, compound **4g** was synthetized from compound **3g** in 74% yield as a brown oil.

**5-(2-Ethoxyethoxy)pentanal (4h)**. Following the procedure of compound **4a**, compound **4h** was synthetized from compound **3h** in 64% yield as a brown oil.

***N*-(4-(hydrazinecarbonyl)benzyl)benzamide (7)**. Hydrazine hydrate (2.5 g, 49.5 mmol) was added to a solution of compound 6 (888 mg, 3.3 mmol) in MeOH (20 mL). The reaction was refluxed for 24 h, then volatiles were removed under vacuum. The crude product was recrystallized by MeOH to obtain compound **7**, as a white solid (665 mg, 83%).

The synthesis of compounds **A1**-**A7** was carried out according to the methods described in the literature^[1]^.

***N*-(4-(2-ndecylhydrazine-1-carbonyl)benzyl)benzamide (A8)**. 1 mmol of MgSO_4_ was added to a solution of compound **7** (53.8 mg, 0.2 mmol) in 20 mL of MeOH. The reaction was stirred at rt for 30 min. Undecanal (35.7 mg, 0.2 mmol) was added to the mixture. 2 h later, NaBH_3_CN (37.2 mg, 0.6 mmol) and 2 drops of concentrated HCl/ MeOH (1:1, *v/v*) solution was added. The mixture was allowed to stir overnight, volatiles were removed under vacuum, and the crude product was purified by via flash chromatography to afford the compound **A8**, a white solid powder (54.1 mg, 64%). ^1^H NMR (500 MHz, DMSO-*d*_6_) δ 9.96 (s, 1H), 9.08 (t, *J* = 6.0 Hz, 1H), 7.91-7.86 (m, 2H), 7.79-7.73 (m, 2H), 7.57-7.50 (m, 1H), 7.50-7.44 (m, 2H), 7.37 (d, *J* = 8.3 Hz, 2H), 5.08 (s, 1H), 4.51 (d, *J* = 6.0 Hz, 2H), 2.75 (t, *J* = 7.1 Hz, 2H), 1.48-1.38 (m, 2H), 1.34-1.15 (m, 16H), 0.87-0.81 (m, 3H). ^13^C NMR (126 MHz, DMSO-*d*_6_) δ 166.76, 165.58, 143.48, 134.66, 132.17, 131.78, 128.81, 127.69, 127.53, 127.43, 51.65, 42.85, 31.74, 29.46, 29.41, 29.15, 28.04, 27.10, 22.54, 14.40. HRMS (ESI) for C_26_H_36_N_3_O_2_ [M-H]^-^ calcd 422.28130, found 422.28137.

***N*-(4-(2-dodecylhydrazine-1-carbonyl)benzyl)benzamide (A9)***.* Following the procedure of compound **A8**, compound **A9** was synthetized from compound **7** and dodecyl aldehyde in 51% yield as a white solid. ^1^H NMR (400 MHz, DMSO-*d*_6_) δ 9.93 (s, 1H), 9.06 (t, *J* = 6.0 Hz, 1H), 7.89-7.82 (m, 2H), 7.74 (d, *J* = 8.3 Hz, 2H), 7.55-7.48 (m, 1H), 7.46-7.40 (m, 2H), 7.34 (d, *J* = 8.1 Hz, 2H), 4.48 (d, *J* = 6.0 Hz, 2H), 2.72 (t, *J* = 7.1 Hz, 2H), 1.47-1.35 (m, 2H), 1.20 (s, 18H), 0.83-0.78 (m, 3H). ^13^C NMR (100 MHz, DMSO-*d*_6_) δ 166.8, 165.6, 143.5, 134.7, 132.3, 131.8, 128.9, 127.8, 127.6, 127.5, 51.7, 42.9, 31.8, 29.6, 29.5, 29.4, 29.2, 28.1, 27.2, 22.6, 14.5. HRMS (ESI) for C_27_H_38_N_3_O_2_ [M − H]^−^ calcd 436.29695, found 436.29691.

***N*-(4-(2-(10-hydroxydecyl)hydrazine-1-carbonyl)benzyl)benzamide (B1)**. Following the procedure of compound **A8**, compound **B1** was synthetized from compound **7** and **4a** in 45% yield as a white solid. ^1^H NMR (400 MHz, DMSO-*d*_6_) δ 9.95 (s, 1H), 9.09 (t, *J* = 6.0 Hz, 1H), 7.86 (d, *J* = 7.2 Hz, 2H), 7.74 (d, *J* = 8.0 Hz, 2H), 7.51 (t, *J* = 7.2 Hz, 1H), 7.44 (t, *J* = 7.4 Hz, 2H), 7.34 (d, *J* = 8.0 Hz, 2H), 5.06 (s, 1H), 4.48 (d, *J* = 6.0 Hz, 2H), 4.30 (t, *J* = 5.2 Hz, 1H), 2.72 (t, *J* = 7.1 Hz, 2H), 1.46-1.32 (m, 4H), 1.30 -1.19 (m, 12H). ^13^C NMR (101 MHz, DMSO-*d*_6_) δ 166.80, 165.66, 143.58, 134.71, 132.24, 131.88, 128.91, 127.79, 127.62, 127.51, 61.24, 51.73, 42.90, 33.08, 29.62, 29.54, 29.51, 28.15, 27.21, 26.04. HRMS (ESI) for C_25_H_34_N_3_O_3_ [M-H]^-^ calcd 424.26057, found 424.26059.

***N*-(4-(2-(9-methoxynonyl)hydrazine-1-carbonyl)benzyl)benzamide (B2)**. Following the procedure of compound **A8**, compound **B2** was synthetized from compound **7** and **4b** in 57% yield as a white solid. ^1^H NMR (400 MHz, DMSO-*d*_6_) δ 9.94 (s, 1H), 9.07 (t, *J* = 6.0 Hz, 1H), 7.88-7.82 (m, 2H), 7.74 (d, *J* = 8.3 Hz, 2H), 7.55-7.48 (m, 1H), 7.44 (dd, *J* = 8.2, 6.5 Hz, 2H), 7.36-7.30 (m, 2H), 5.04 (s, 1H), 4.48 (d, *J* = 6.0 Hz, 2H), 3.24 (t, *J* = 6.5 Hz, 2H), 2.72 (t, *J* = 7.1 Hz, 2H), 1.46-1.36 (m, 4H), 1.28-1.18 (m, 10H). ^13^C NMR (101 MHz, DMSO-*d*_6_) δ 166.79, 165.65, 143.57, 134.72, 132.26, 131.87, 128.90, 127.79, 127.62, 127.50, 72.42, 58.31, 51.72, 42.90, 29.56, 29.53, 29.47, 29.37, 28.14, 27.18, 26.20. HRMS (ESI) for C_25_H_34_N_3_O_3_ [M-H]^-^ calcd 424.26057, found 424.26059.

***N*-(4-(2-(8-ethoxyoctyl)hydrazine-1-carbonyl)benzyl)benzamide (B3)**. Following the procedure of compound **A8**, compound **B3** was synthetized from compound **7** and **4c** in 55% yield as a white solid. ^1^H NMR (400 MHz, DMSO-*d*_6_) δ 9.94 (s, 1H), 9.07 (t, *J* = 5.9 Hz, 1H), 7.89-7.82 (m, 2H), 7.77-7.69 (m, 2H), 7.51 (t, *J* = 7.2 Hz, 1H), 7.47-7.41 (m, 2H), 7.34 (d, *J* = 8.1 Hz, 2H), 5.03 (s, 1H), 4.48 (d, *J* = 5.9 Hz, 2H), 3.34 (d, *J* = 6.9 Hz, 2H), 3.27 (t, *J* = 6.6 Hz, 2H), 2.72 (t, *J* = 7.1 Hz, 2H), 1.45-1.37 (m, 4H), 1.26 -1.19 (m, 8H), 1.04 (t, *J* = 6.9 Hz, 3H). ^13^C NMR (101 MHz, DMSO-*d*_6_) δ 166.79, 165.66, 143.57, 134.72, 132.25, 131.87, 128.90, 127.78, 127.62, 127.50, 70.26, 65.70, 51.73, 42.90, 29.81, 29.50, 29.41, 28.13, 27.16, 26.25, 15.69. HRMS (ESI) for C_25_H_34_N_3_O_3_ [M-H]^-^ calcd 424.26057, found 424.26065.

***N*-(4-(2-(7-propoxyheptyl)hydrazine-1-carbonyl)benzyl)benzamide (B4)**. Following the procedure of compound **A8**, compound **B4** was synthetized from compound **7** and **4d** in 51% yield as a white solid. ^1^H NMR (400 MHz, DMSO-*d*_6_) δ 9.94 (d, *J* = 5.4 Hz, 1H), 9.07 (t, *J* = 6.0 Hz, 1H), 7.91-7.81 (m, 2H), 7.74 (d, *J* = 8.3 Hz, 2H), 7.56-7.48 (m, 1H), 7.48-7.41 (m, 2H), 7.34 (d, *J* = 8.2 Hz, 2H), 5.01 (d, *J* = 6.5 Hz, 1H), 4.48 (d, *J* = 6.0 Hz, 2H), 3.28 (t, *J* = 6.5 Hz, 2H), 3.24 (t, *J* = 6.6 Hz, 2H), 2.76-2.68 (m, 2H), 1.47-1.36 (m, 6H), 1.30-1.21 (m, 6H), 0.80 (t, *J* = 7.4 Hz, 3H). ^13^C NMR (101 MHz, DMSO-*d*_6_) δ 166.80, 165.66, 143.57, 134.72, 132.27, 131.87, 128.90, 127.79, 127.62, 127.50, 72.08, 70.43, 51.72, 42.90, 29.76, 29.36, 28.12, 27.19, 26.25, 23.01, 11.13. HRMS (ESI) for C_25_H_34_N_3_O_3_ [M-H]^-^ calcd 424.26057, found 424.26065.

***N*-(4-(2-(6-butoxyhexyl)hydrazine-1-carbonyl)benzyl)benzamide (B5)**. Following the procedure of compound **A8**, compound **B5** was synthetized from compound **7** and **4e** in 56% yield as a white solid. ^1^H NMR (400 MHz, DMSO-*d*_6_) δ 9.94 (d, *J* = 5.8 Hz, 1H), 9.07 (t, *J* = 6.0 Hz, 1H), 7.89-7.83 (m, 2H), 7.74 (d, *J* = 8.3 Hz, 2H), 7.54-7.48 (m, 1H), 7.48-7.41 (m, 2H), 7.34 (d, *J* = 8.3 Hz, 2H), 5.02 (d, *J* = 5.9 Hz, 1H), 4.48 (d, *J* = 6.0 Hz, 2H), 3.28 (t, *J* = 6.5 Hz, 4H), 2.76-2.68 (m, 2H), 1.48-1.35 (m, 6H), 1.34-1.22 (m, 6H), 0.82 (t, *J* = 7.3 Hz, 3H). ^13^C NMR (101 MHz, DMSO-*d*_6_) δ 166.80, 165.66, 143.57, 134.73, 132.27, 131.87, 128.90, 127.79, 127.62, 127.51, 70.44, 70.14, 51.70, 42.91, 31.88, 29.76, 28.15, 27.05, 26.22, 19.44, 14.32. HRMS (ESI) for C_25_H_34_N_3_O_3_ [M-H]^-^ calcd 424.26057, found 424.26065.

***N*-(4-(2-(5-(Pentyloxy)pentyl)hydrazine-1-carbonyl)benzyl)benzamide (B6)**. Following the procedure of compound **A8**, compound **B6** was synthetized from compound **7** and **4f** in 61% yield as a white solid. ^1^H NMR (400 MHz, DMSO-*d*_6_) δ 9.94 (s, 1H), 9.07 (t, *J* = 6.0 Hz, 1H), 7.91-7.82 (m, 2H), 7.74 (d, *J* = 8.3 Hz, 2H), 7.54-7.48 (m, 1H), 7.47-7.41 (m, 2H), 7.34 (d, *J* = 8.1 Hz, 2H), 5.04 (s, 1H), 4.48 (d, *J* = 6.0 Hz, 2H), 3.31-3.22 (m, 4H), 2.72 (t, *J* = 7.0 Hz, 2H), 1.51-1.36 (m, 6H), 1.36-1.27 (m, 2H), 1.25-1.16 (m, 4H), 0.85-0.76 (m, 3H). ^13^C NMR (101 MHz, DMSO-*d*_6_) δ 166.83, 165.69, 143.57, 134.73, 132.26, 131.86, 129.95, 128.89, 127.79, 127.63, 127.51, 70.48, 70.42, 51.71, 42.92, 29.69, 29.45, 28.46, 27.97, 23.94, 22.49, 14.47. HRMS (ESI) for C_25_H_34_N_3_O_3_ [M-H]^-^ calcd 424.26057, found 424.26047.

***N*-(4-(2-(6-(2-methoxyethoxy)hexyl)hydrazine-1-carbonyl)benzyl)benzamide (B7)**. Following the procedure of compound **A8**, compound **B7** was synthetized from compound **7** and **4g** in 53% yield as a white solid. ^1^H NMR (400 MHz, DMSO-*d*_6_) δ 9.96 (s, 1H), 9.09 (t, *J* = 6.0 Hz, 1H), 7.90-7.84 (m, 2H), 7.75 (d, *J* = 8.3 Hz, 2H), 7.54-7.47 (m, 1H), 7.47-7.41 (m, 2H), 7.35 (d, *J* = 8.1 Hz, 2H), 5.04 (s, 1H), 4.49 (d, *J* = 6.0 Hz, 2H), 3.46-3.36 (m, 5H), 3.31 (t, *J* = 6.6 Hz, 2H), 3.19 (s, 3H), 2.73 (t, *J* = 7.1 Hz, 2H), 1.49-1.35 (m, 4H), 1.34-1.21 (m, 4H). ^13^C NMR (101 MHz, DMSO-*d*_6_) δ 166.83, 165.69, 143.58, 134.73, 132.27, 131.86, 128.89, 127.79, 127.63, 127.53, 71.83, 70.82, 69.81, 58.59, 51.72, 42.93, 29.72, 28.14, 27.05, 26.14. HRMS (ESI) for C_24_H_32_N_3_O_4_ [M-H]^-^ calcd 426.23963, found 426.23962.

***N*-(4-(2-(5-(2-ethoxyethoxy)pentyl)hydrazine-1-carbonyl)benzyl)benzamide (B8)**. Following the procedure of compound **A8**, compound **B8** was synthetized from compound **7** and **4h** in 53% yield as a white solid. ^1^H NMR (400 MHz, DMSO-*d*_6_) δ 10.00 (d, *J* = 7.5 Hz, 1H), 9.12 (t, *J* = 6.0 Hz, 1H), 7.90 (d, *J* = 7.7 Hz, 2H), 7.78 (d, *J* = 8.0 Hz, 2H), 7.59-7.44 (m, 3H), 7.38 (d, *J* = 8.0 Hz, 2H), 4.52 (d, *J* = 6.2 Hz, 2H), 3.48-3.37 (m, 8H), 2.84-2.69 (m, 2H), 1.58-1.40 (m, 4H), 1.39-1.25 (m, 2H), 1.08 (t, *J* = 7.2 Hz, 3H). ^13^C NMR (101 MHz, DMSO-*d*_6_) δ 166.82, 165.68, 143.58, 134.72, 132.26, 131.87, 128.91, 127.80, 127.64, 127.52, 70.81, 70.06, 69.76, 66.08, 51.69, 42.92, 29.65, 27.98, 23.88, 15.66. HRMS (ESI) for C_24_H_32_N_3_O_4_ [M-H]^-^ calcd 426.23963, found 426.24023.

^1^H NMR and ^13^C NMR Spectrums for all the target compounds


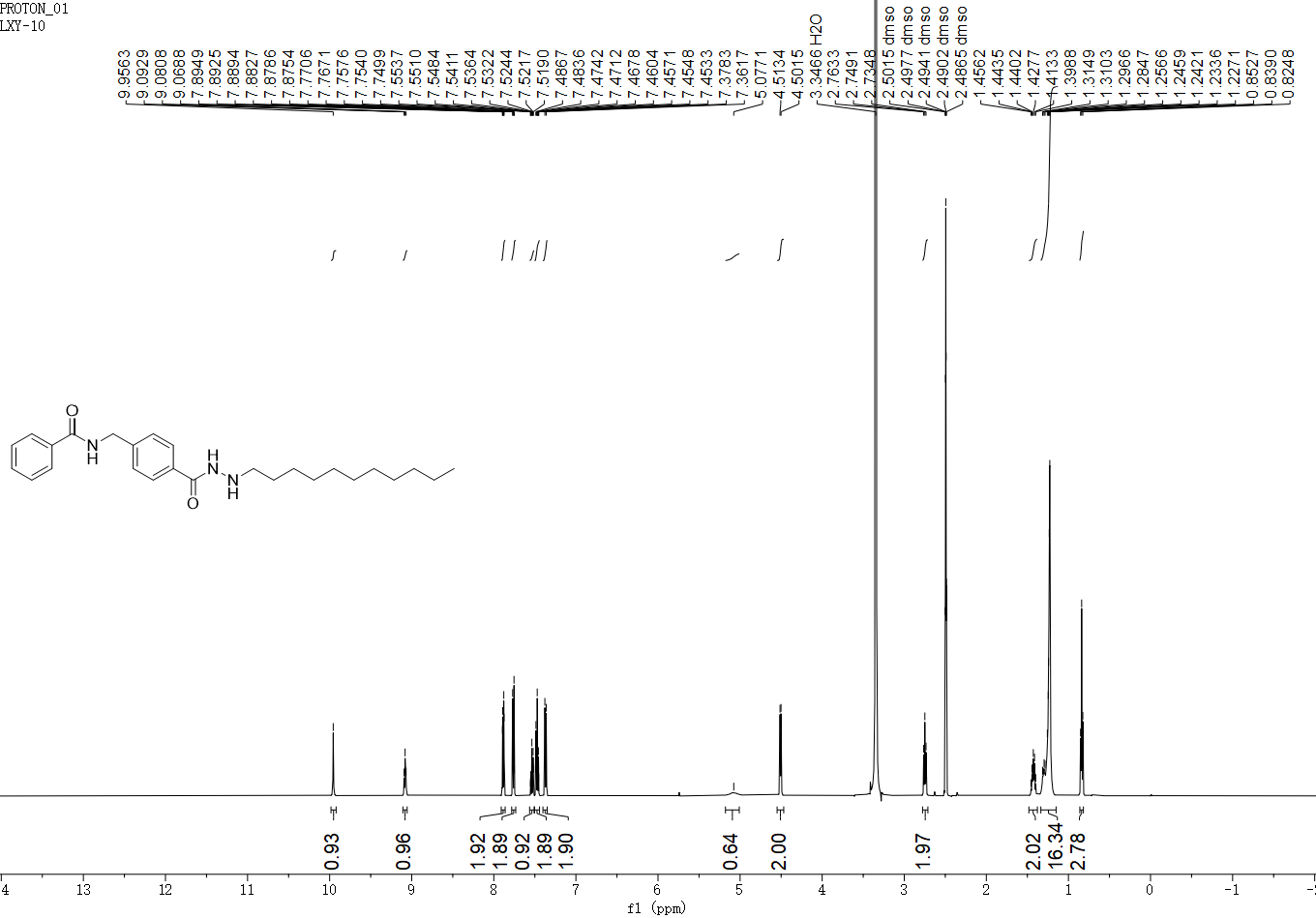


^1^H NMR of **A8** in DMSO-*d*_6_


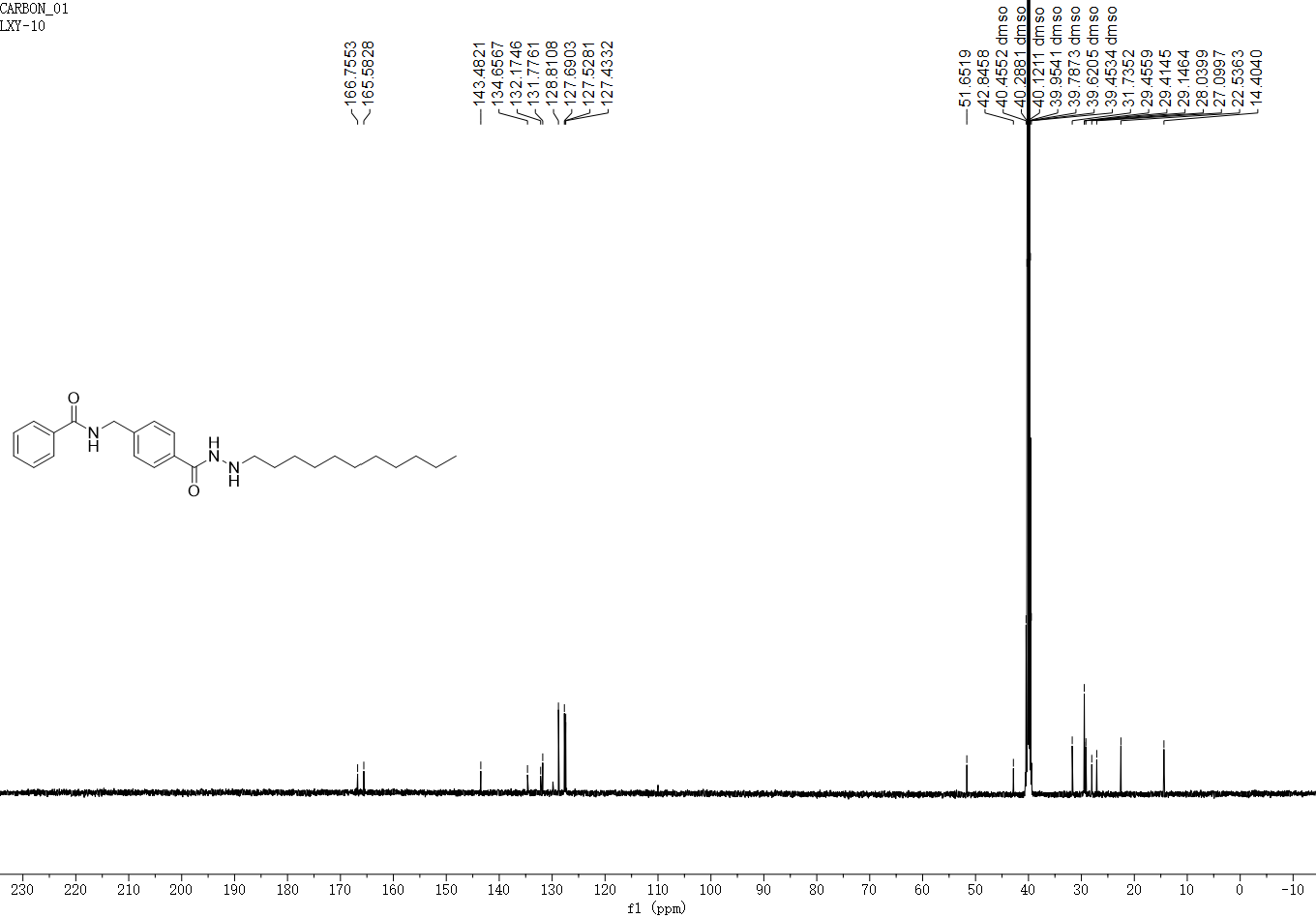


^13^C NMR of **A8** in DMSO-*d*_6_


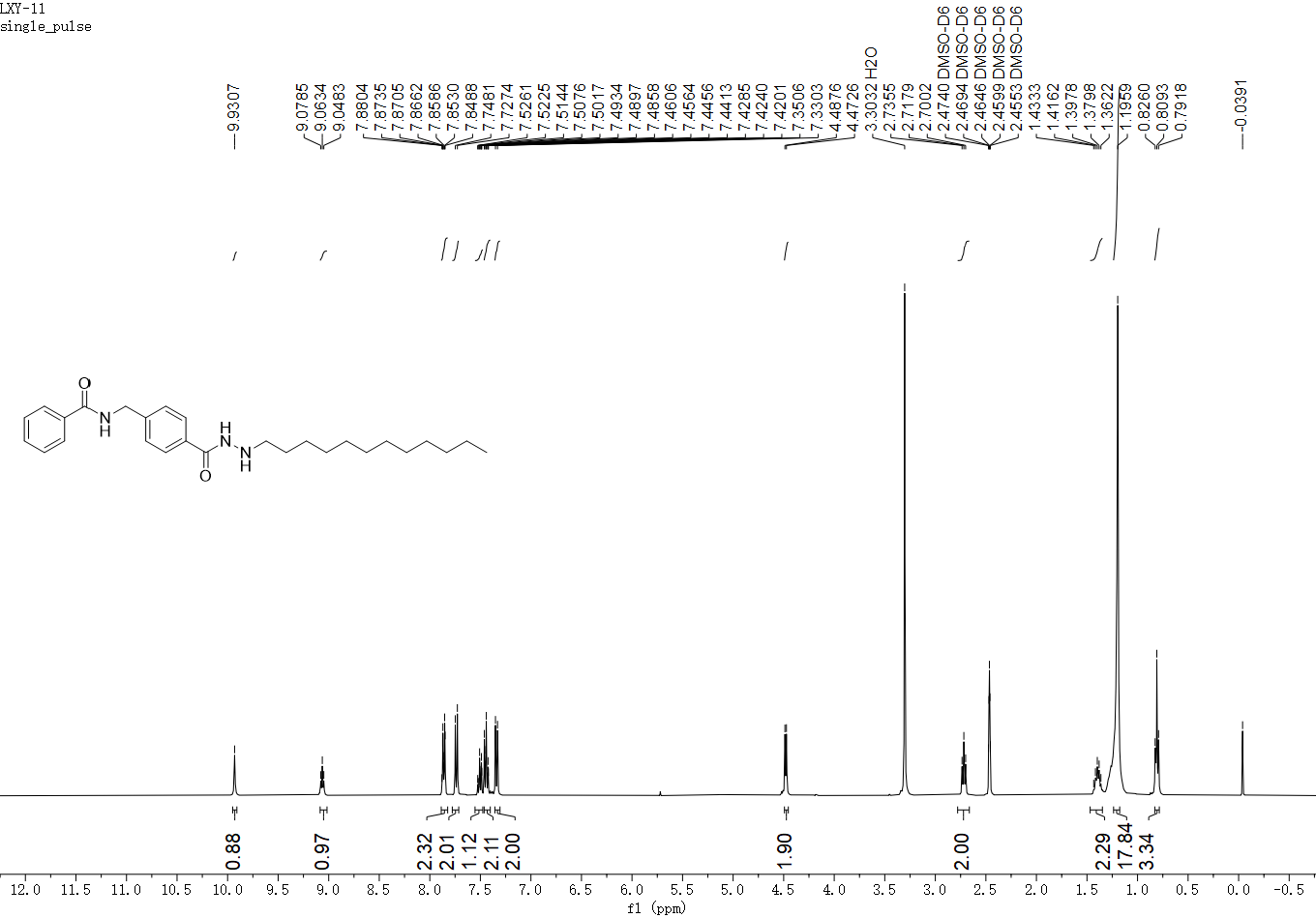
^1^H NMR of **A9** in DMSO-*d*_6_


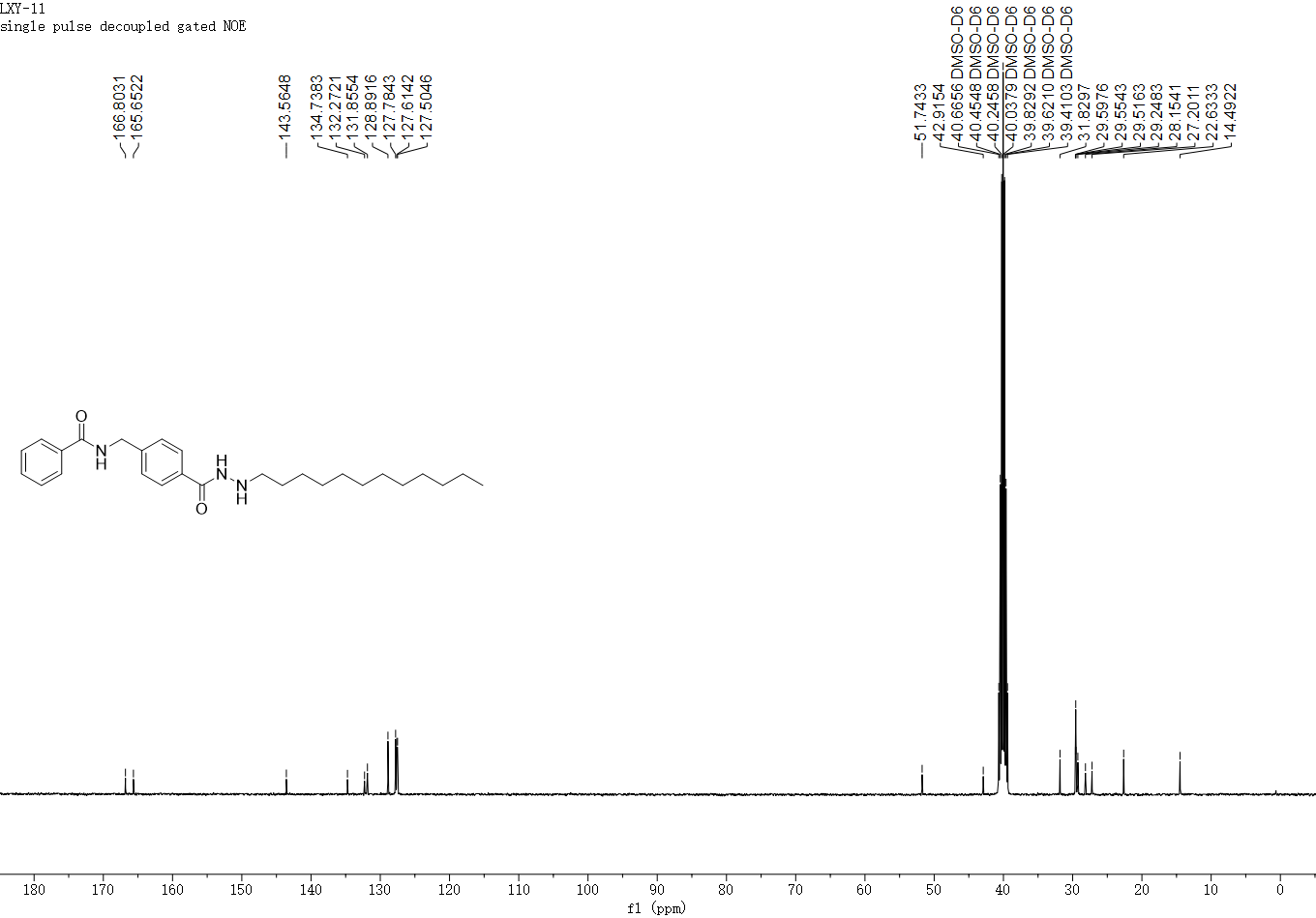
^13^C NMR of **A9** in DMSO-*d*_6_


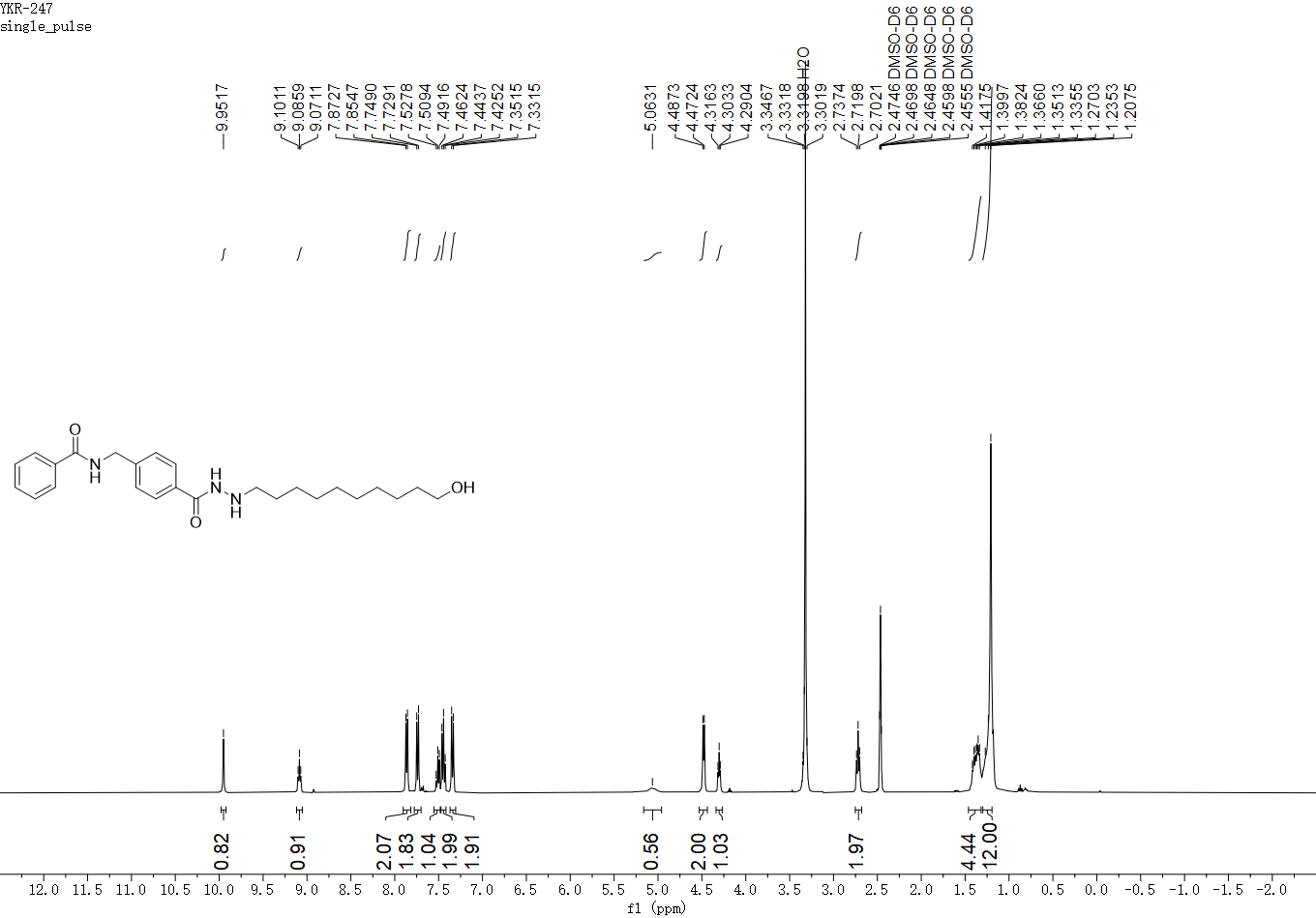


^1^H NMR of **B1** in DMSO-*d*_6_


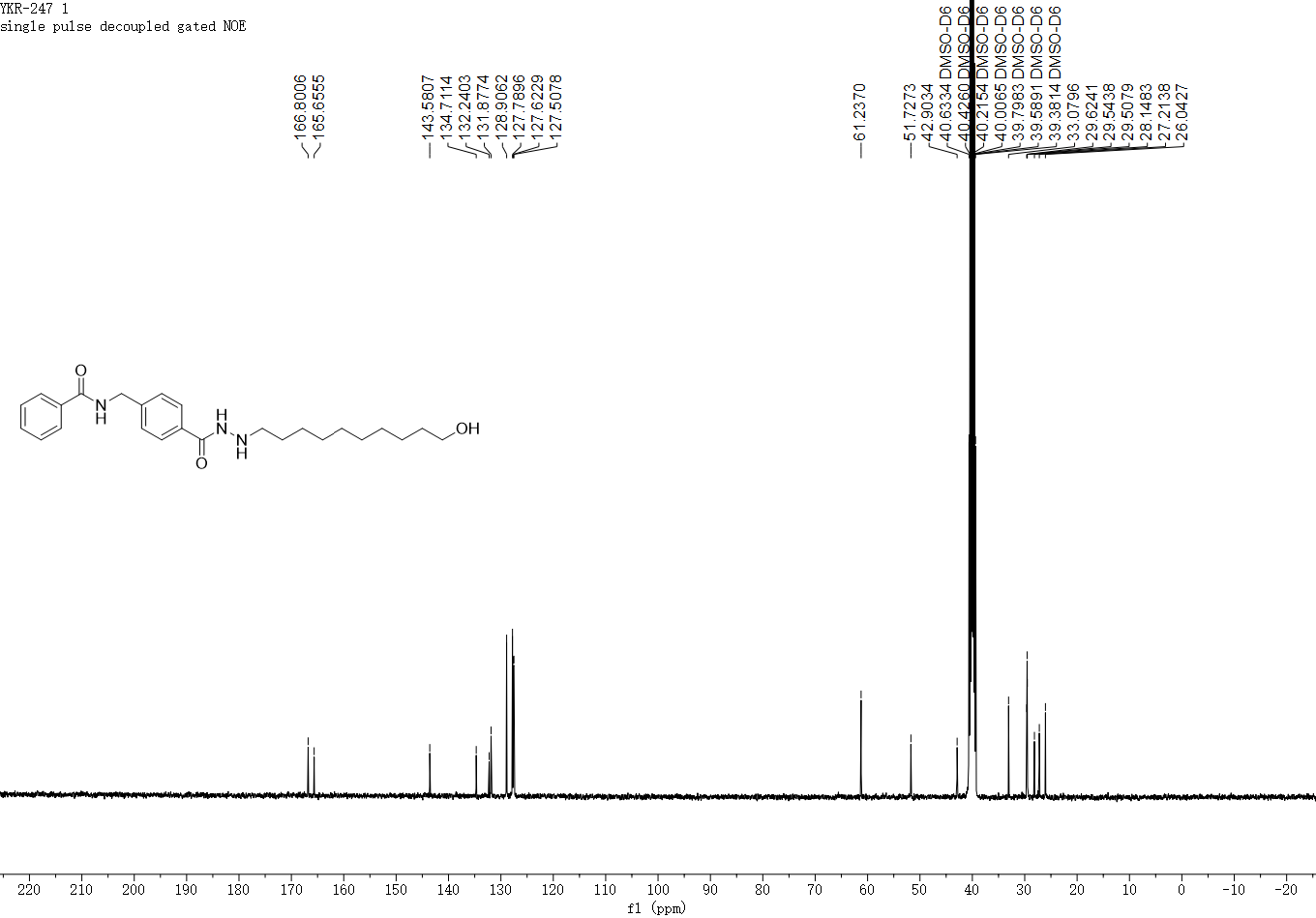


^13^C NMR of **B1** in DMSO-*d*_6_


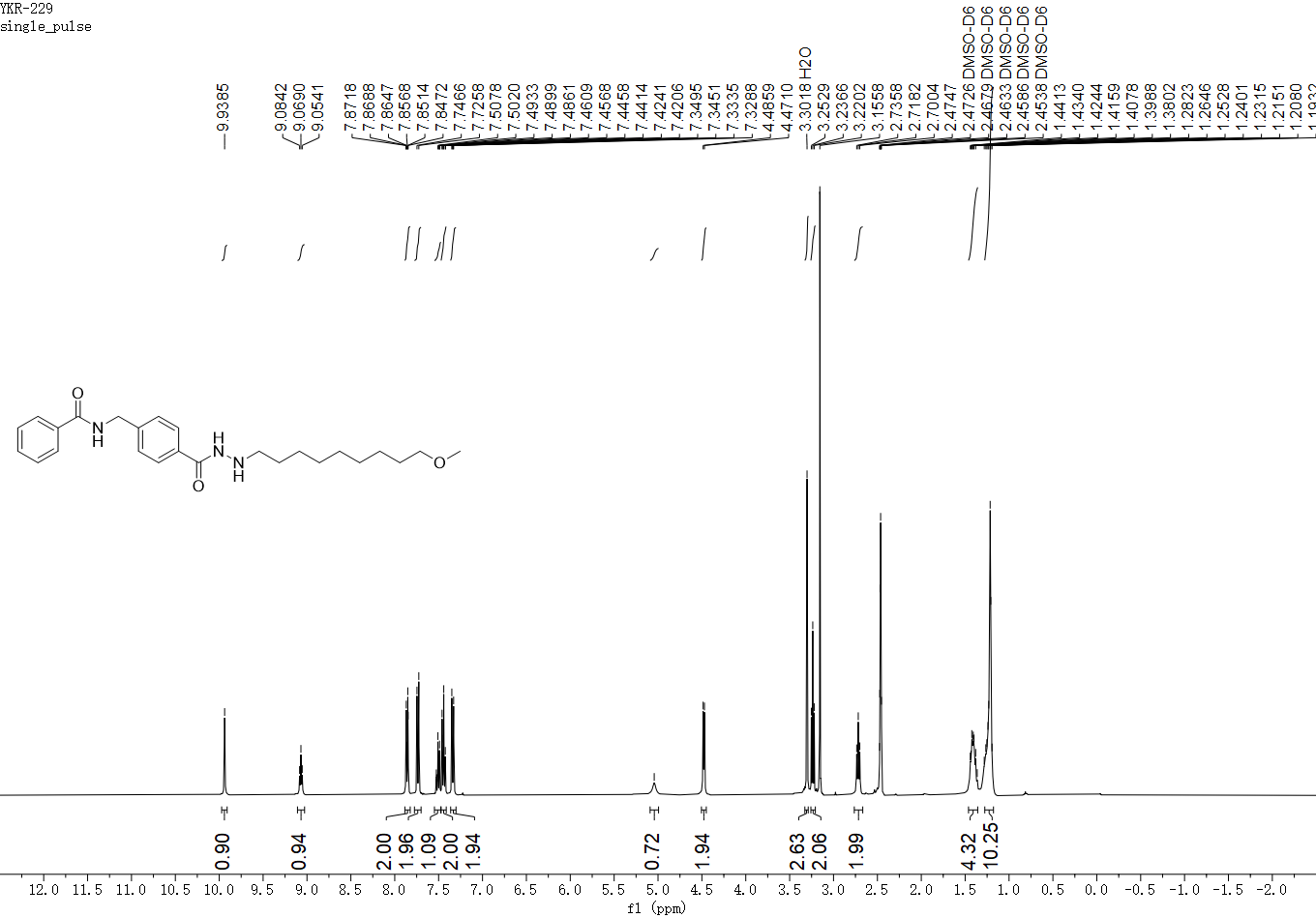


^1^H NMR of **B2** in DMSO-*d*_6_


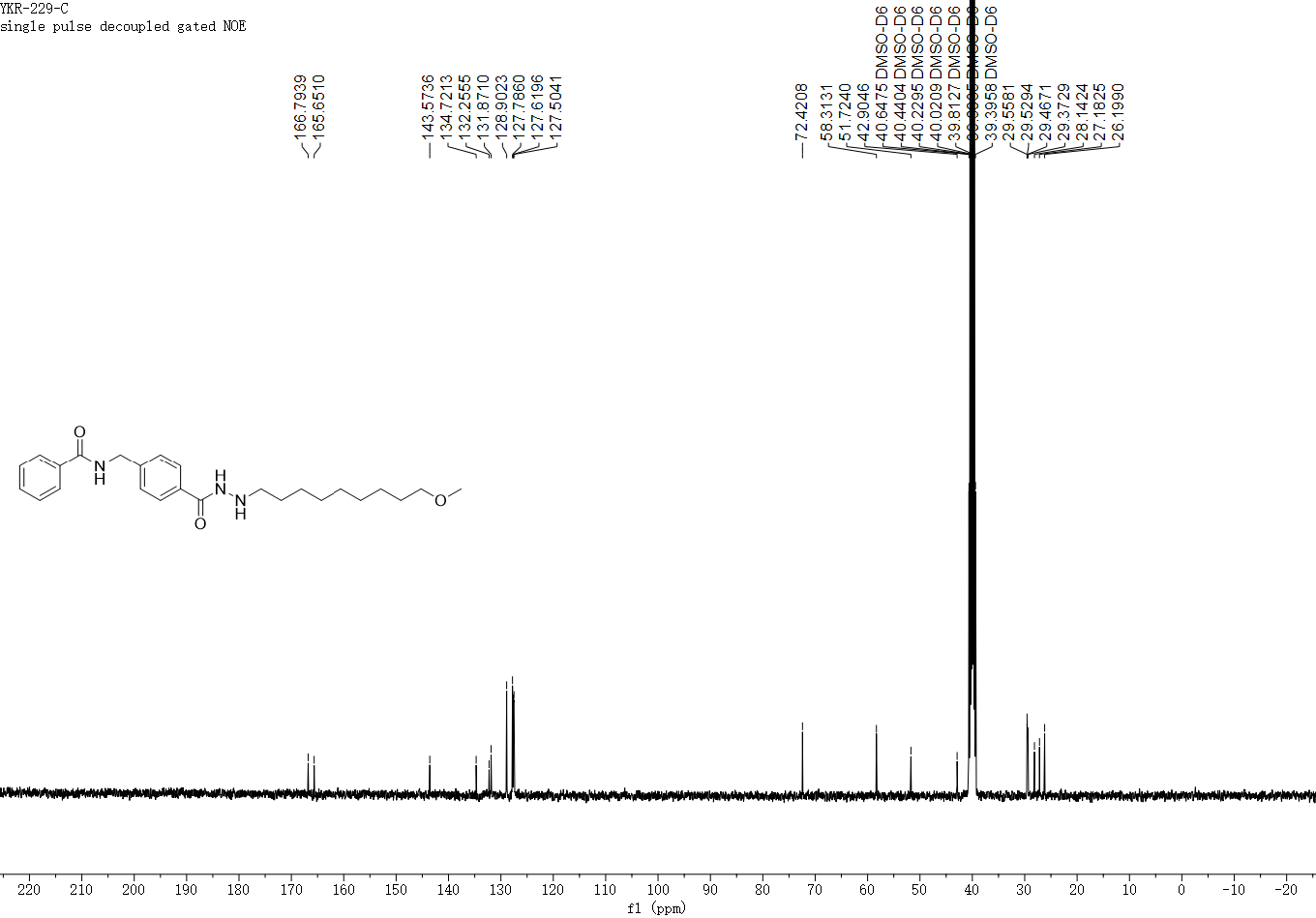


^13^C NMR of **B2** in DMSO-*d*_6_


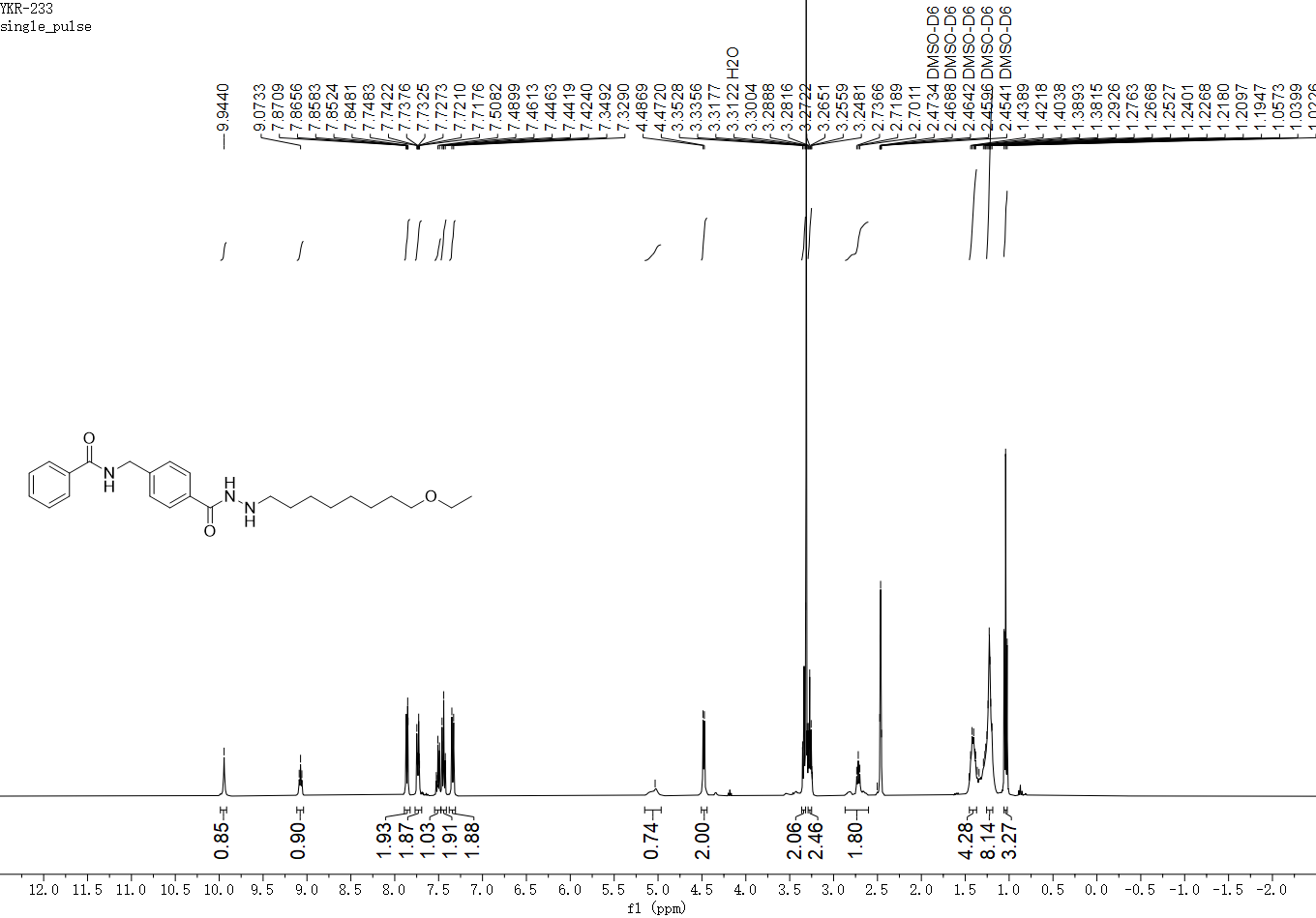


^1^H NMR of **B3** in DMSO-*d*_6_


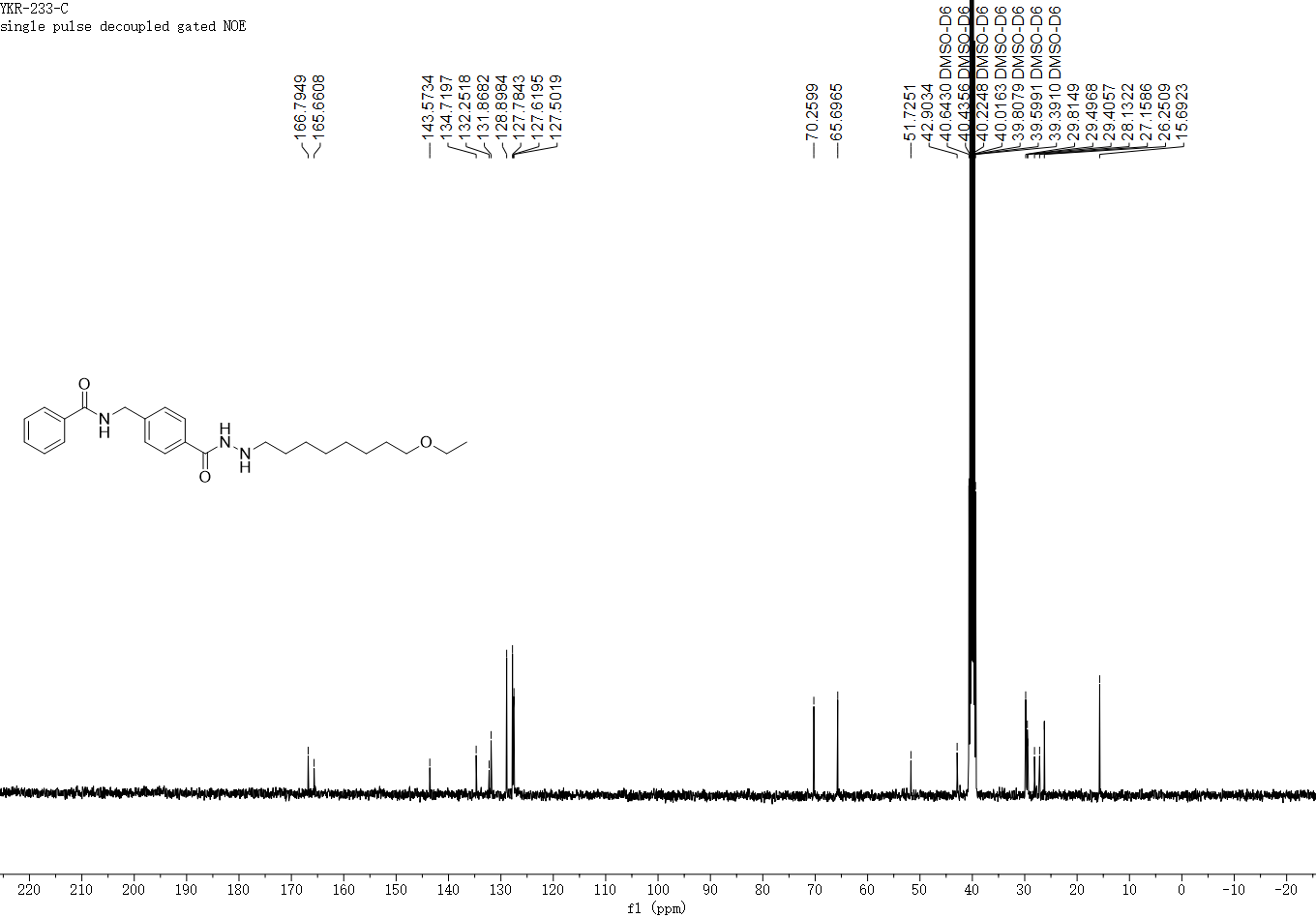


^13^C NMR of **B3** in DMSO-*d*_6_


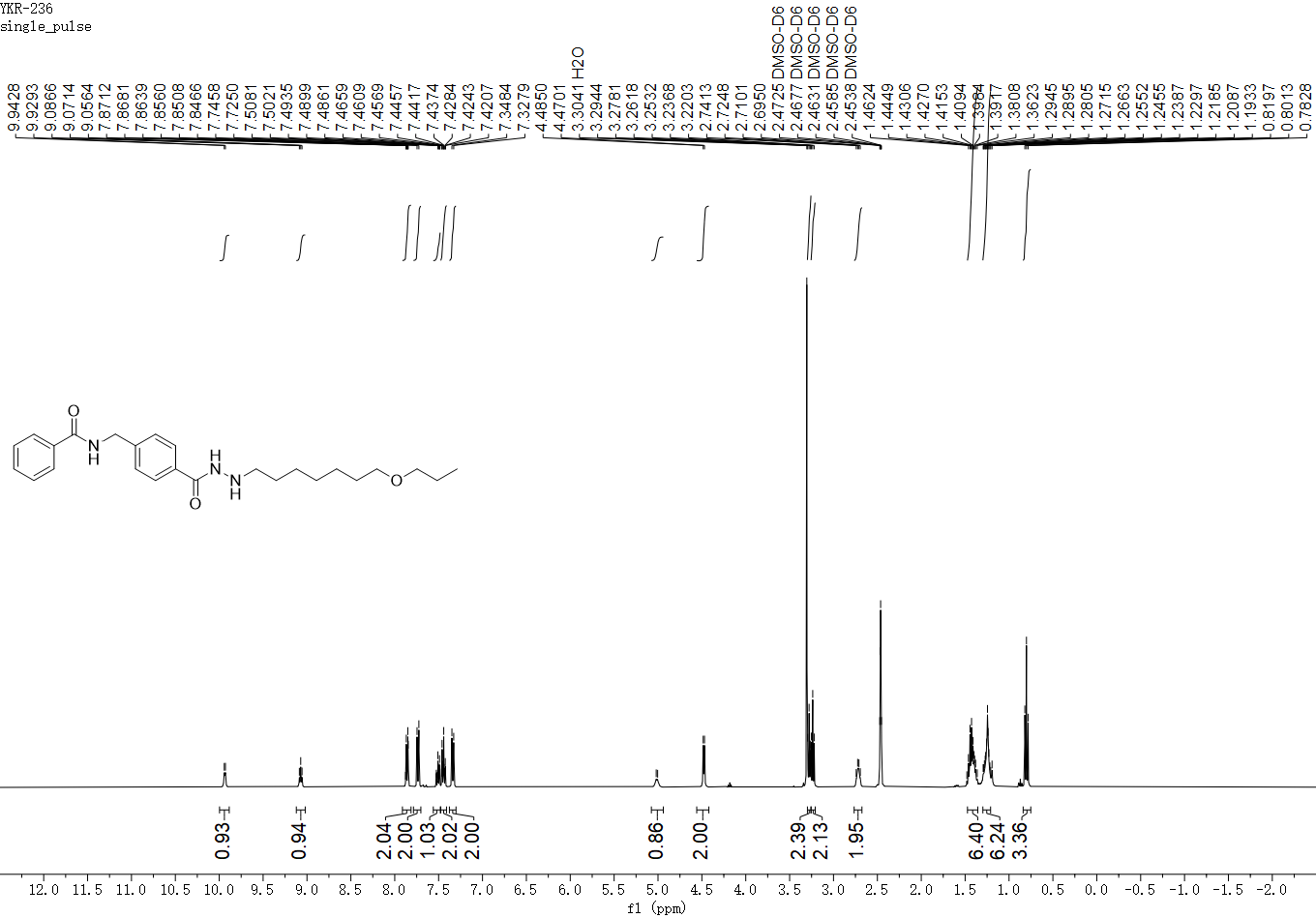


^1^H NMR of **B4** in DMSO-*d*_6_


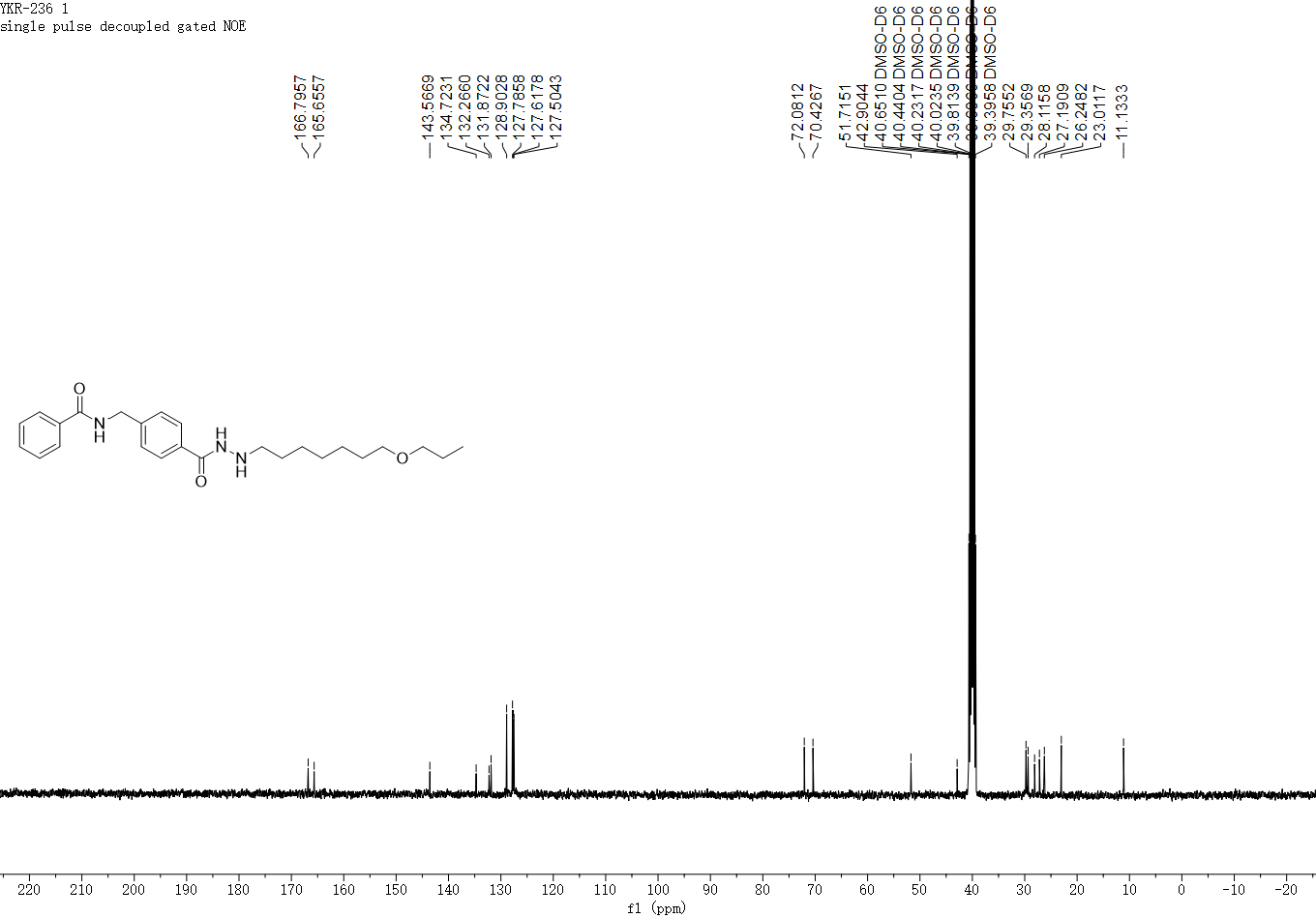


^13^C NMR of **B4** in DMSO-*d*_6_


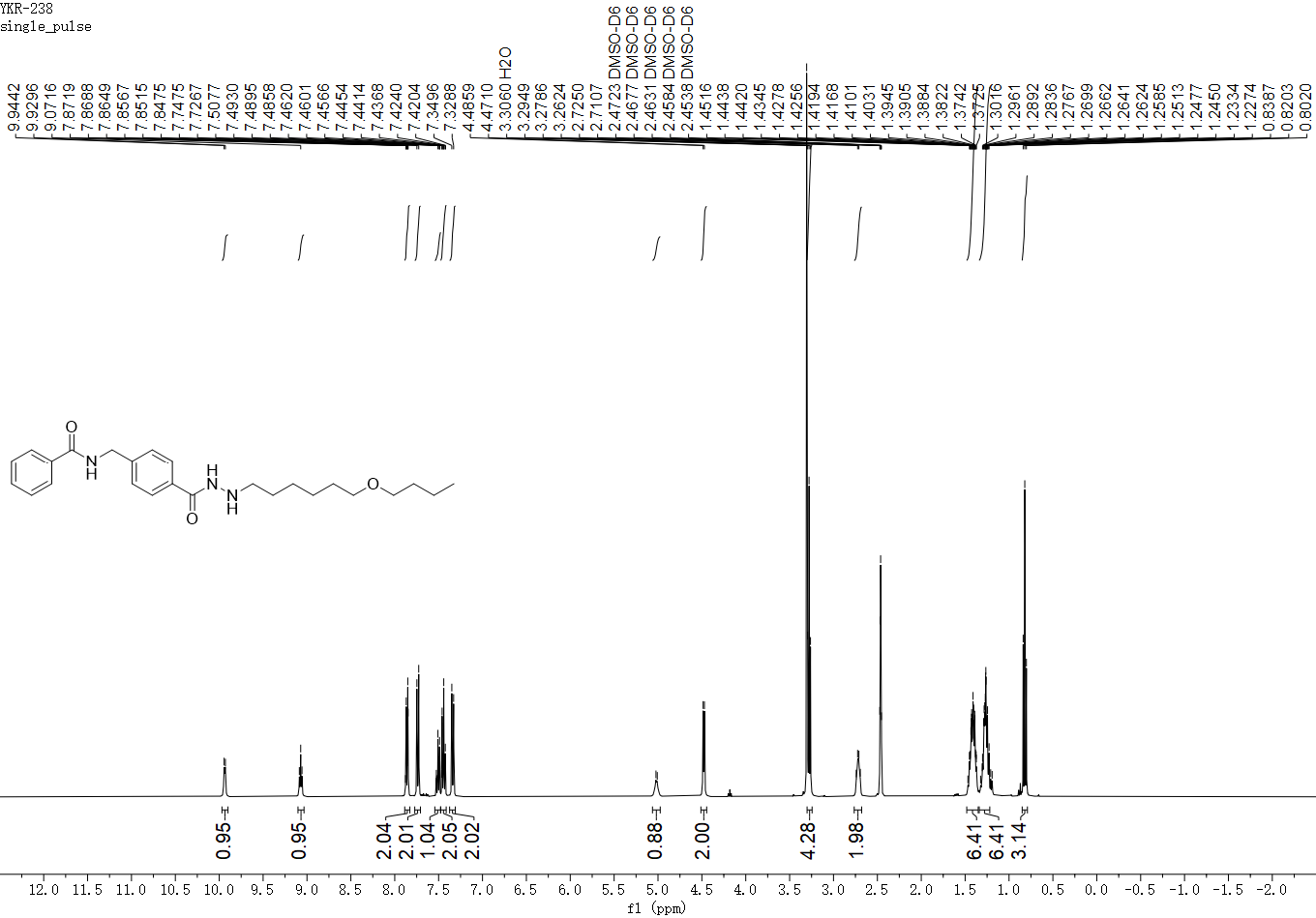


^1^H NMR of **B5** in DMSO-*d*_6_


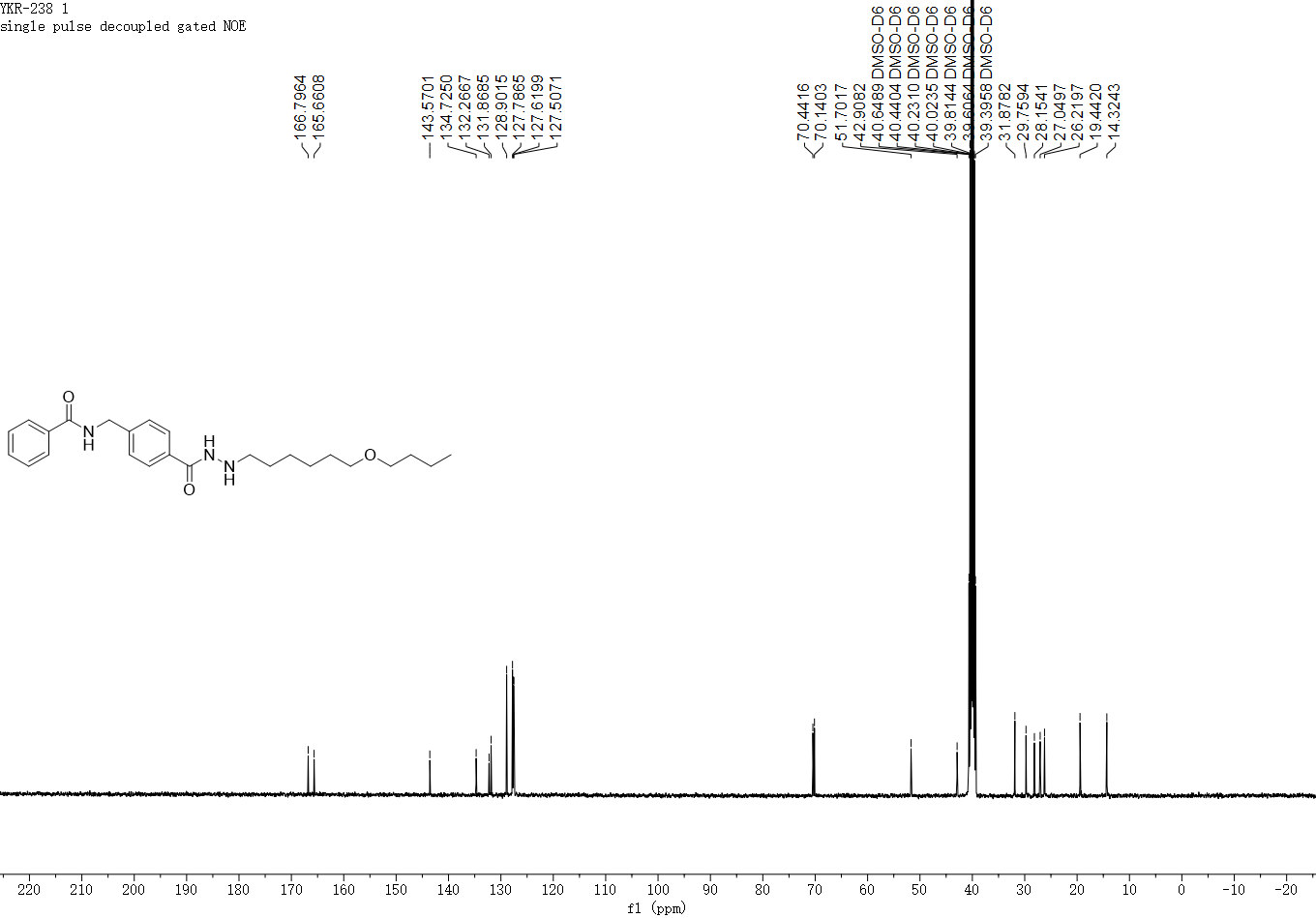


^13^C NMR of **B5** in DMSO-*d*_6_


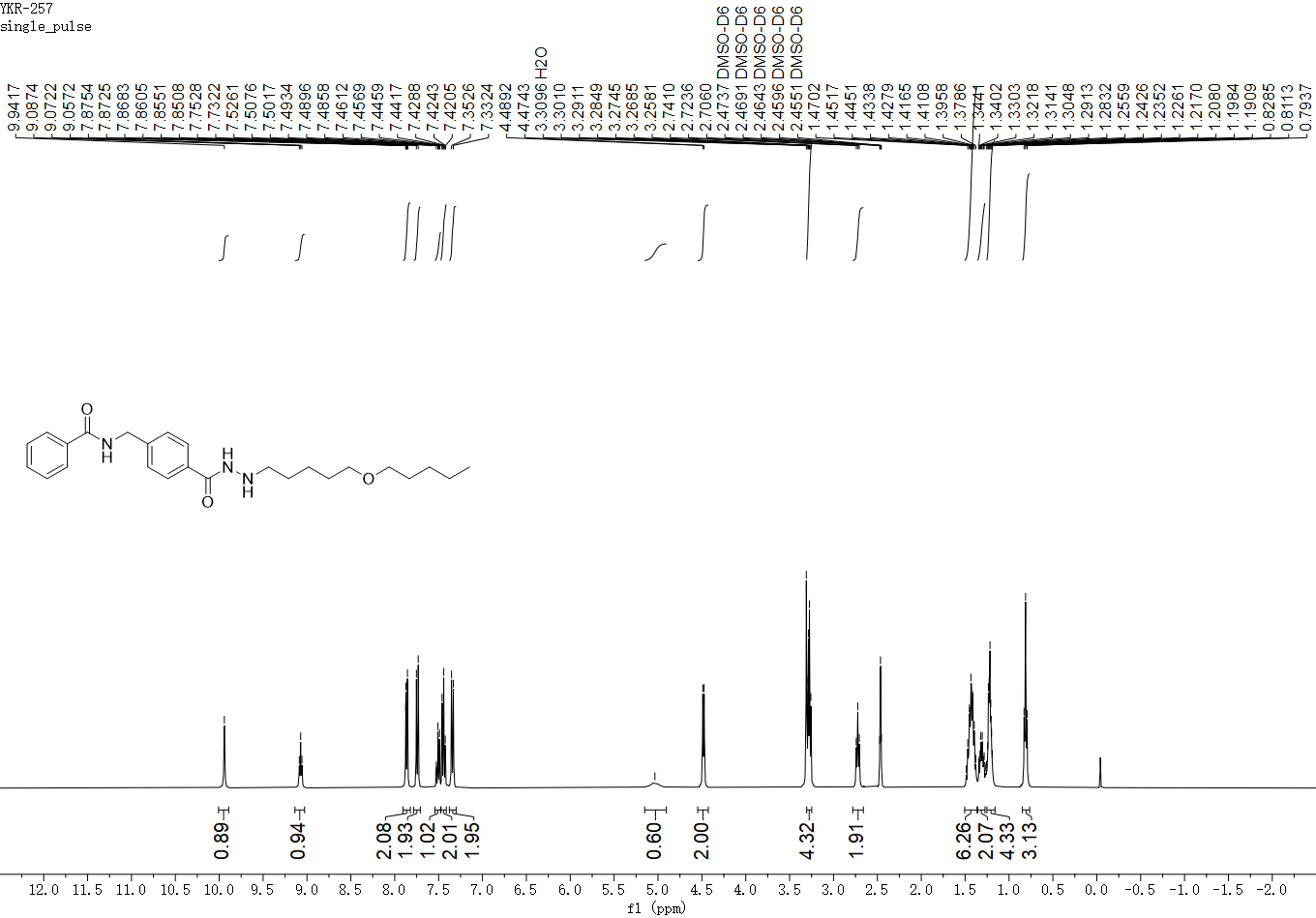


^1^H NMR of **B6** in DMSO-*d*_6_


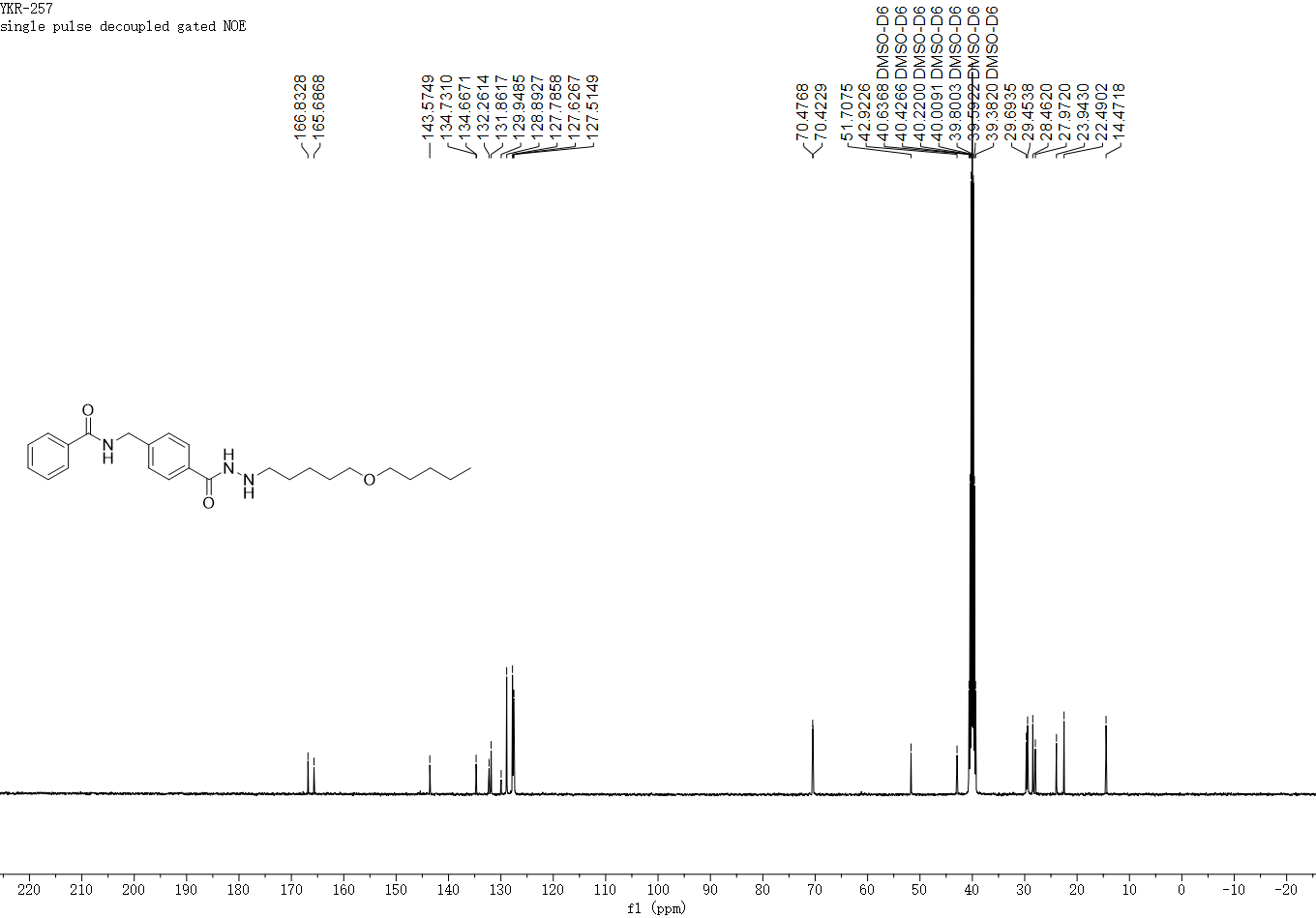


^13^C NMR of **B6** in DMSO-*d*_6_


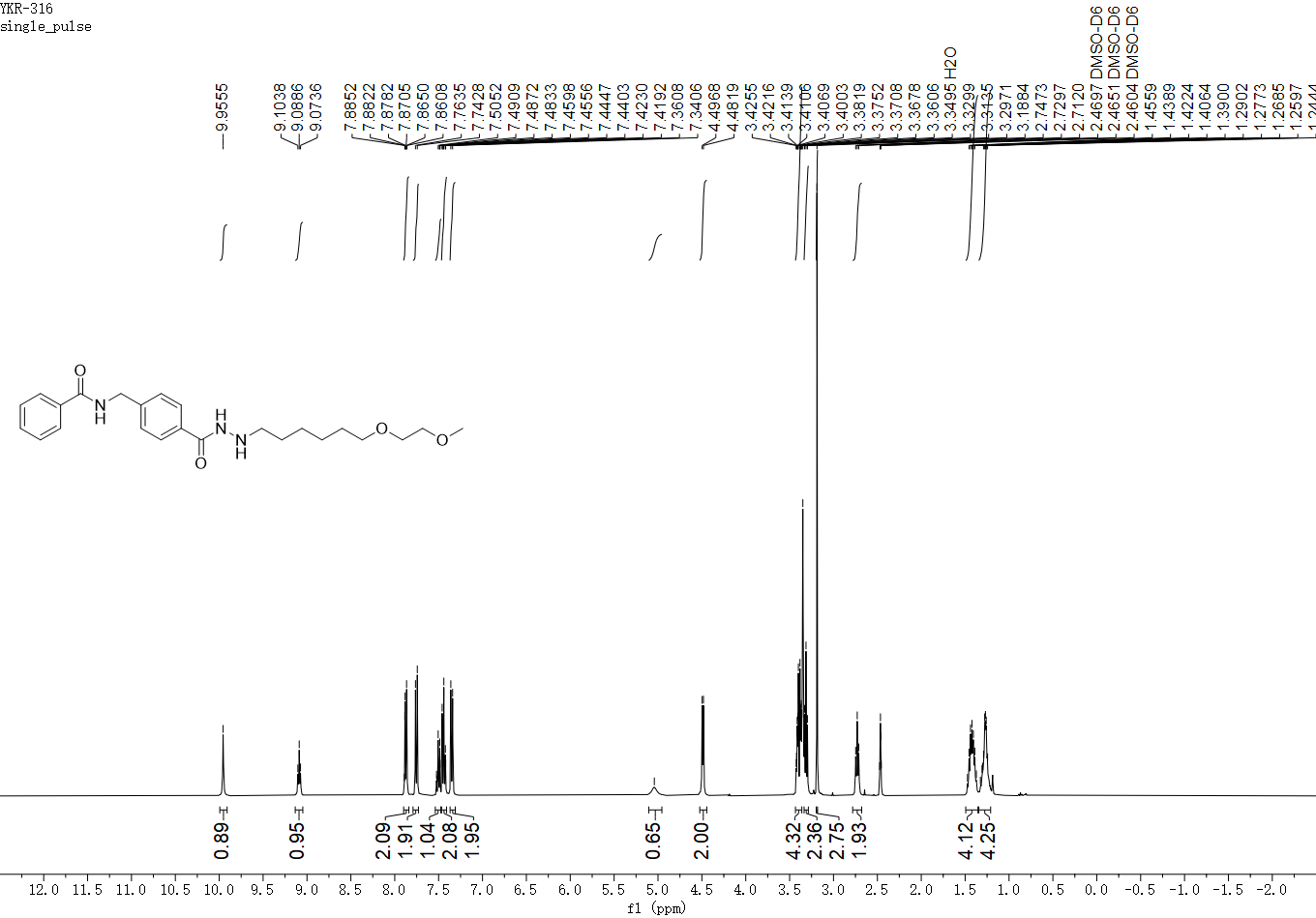


^1^H NMR of **B7** in DMSO-*d*_6_


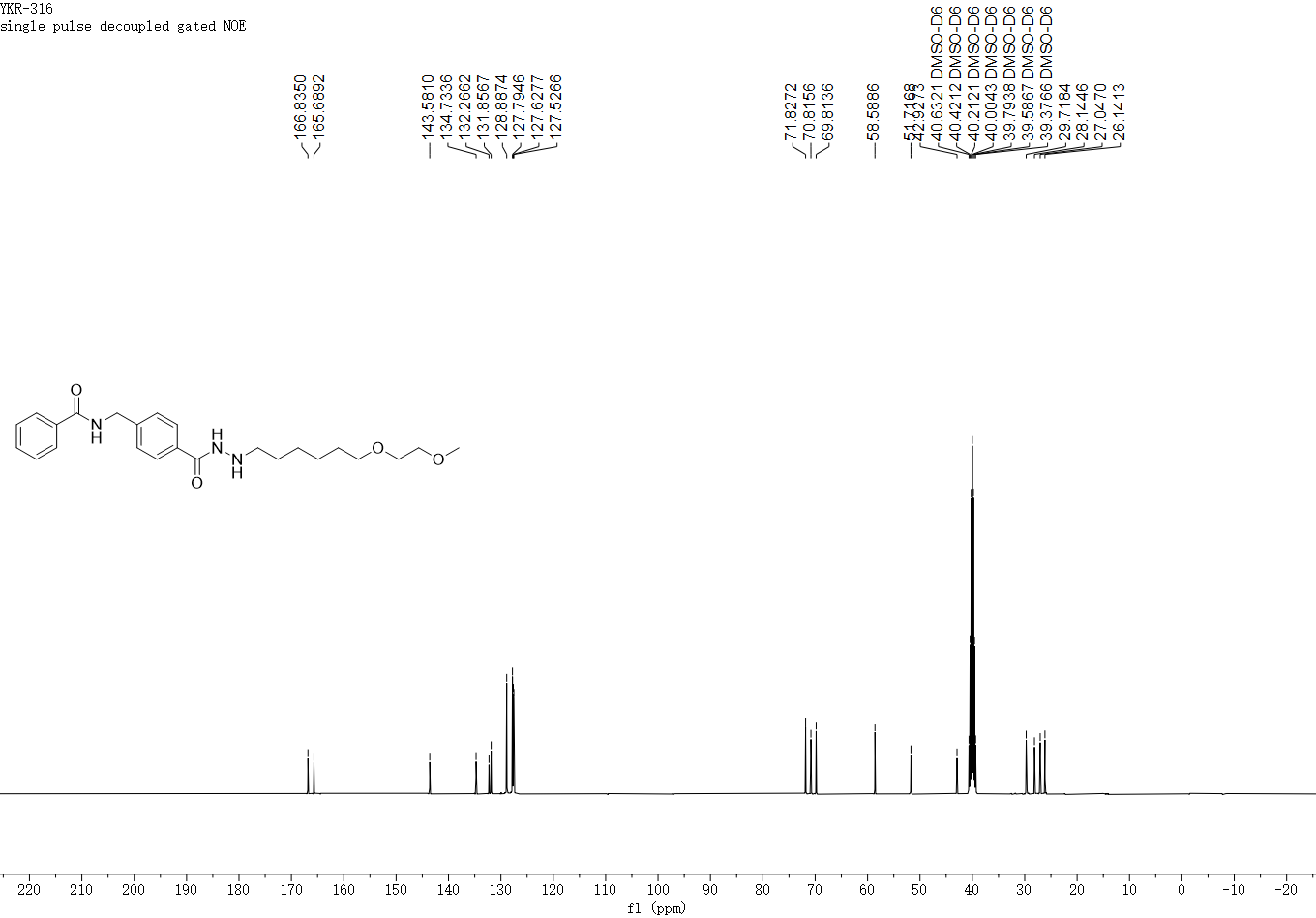


^13^C NMR of **B7** in DMSO-*d*_6_


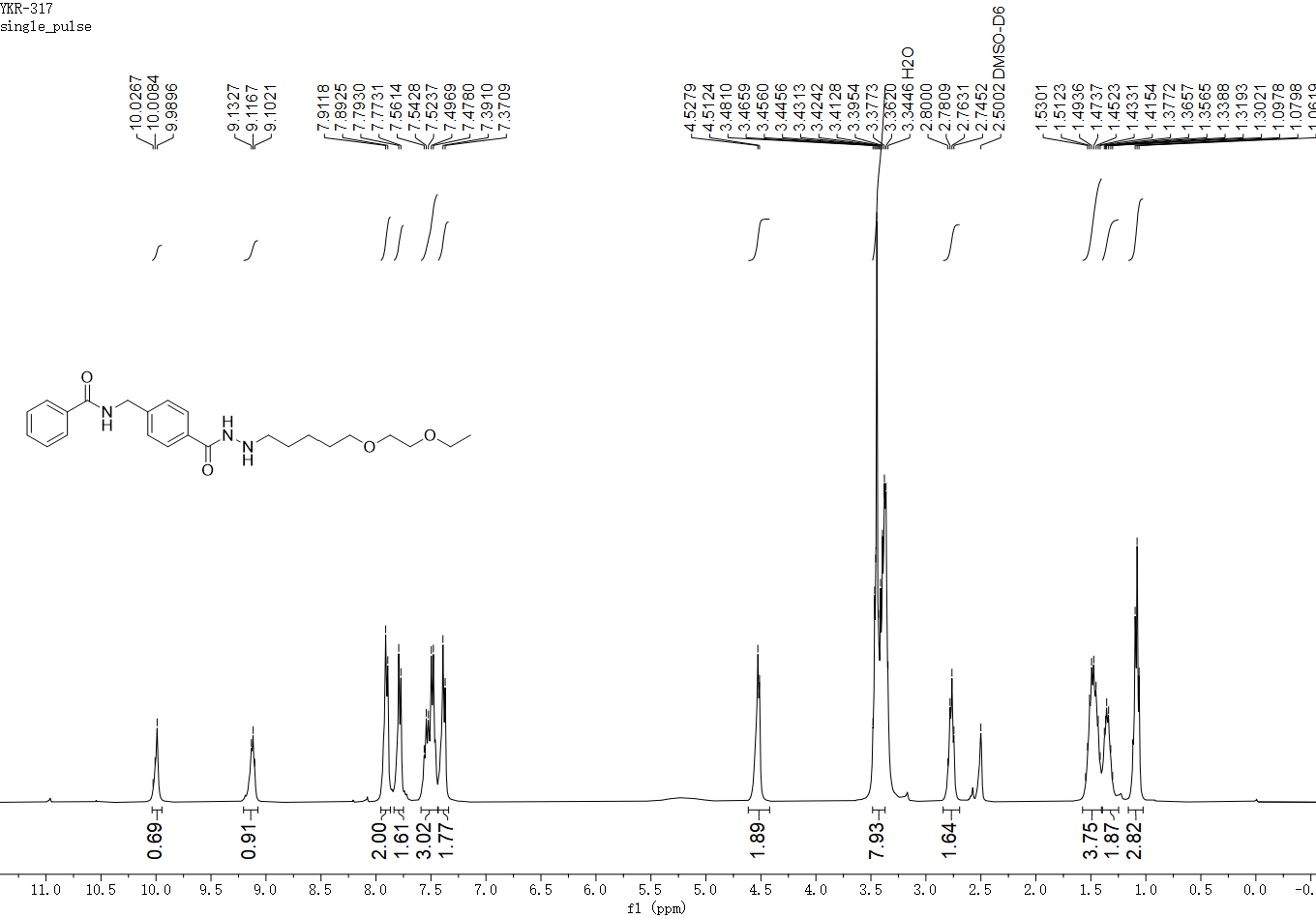


^1^H NMR of **B8** in DMSO-*d*_6_


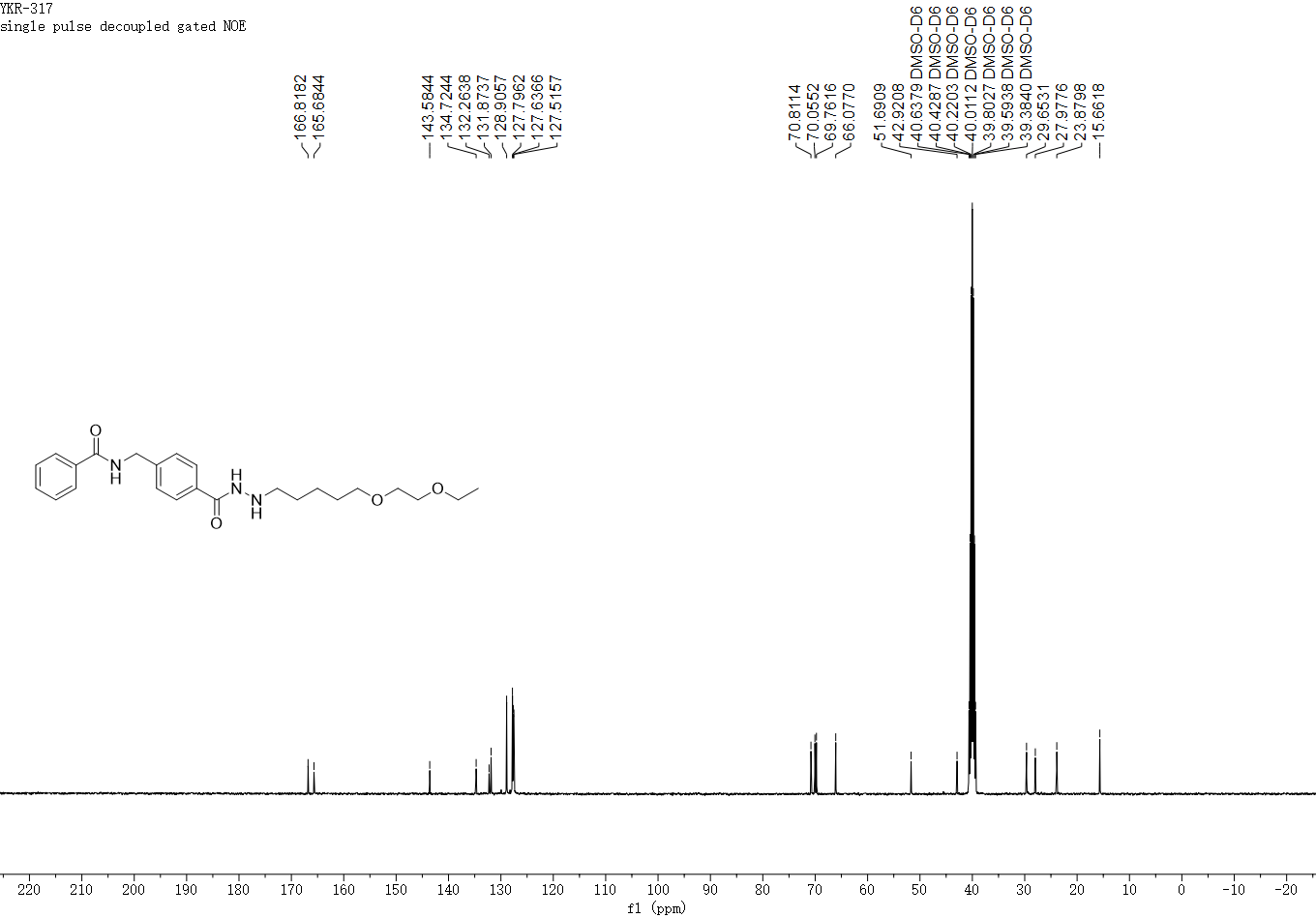


^13^C NMR of **B8** in DMSO-*d*_6_

HPLC traces and purity of the target compounds

**A8** purity: 100%


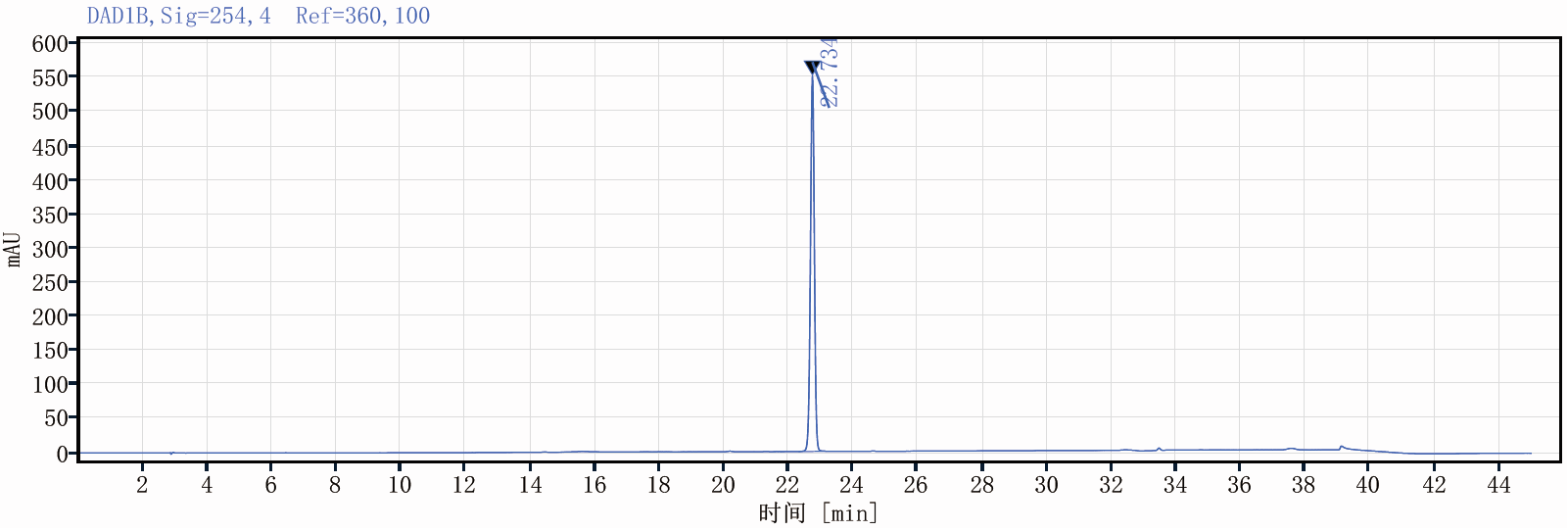


**A9** purity: 98.46%


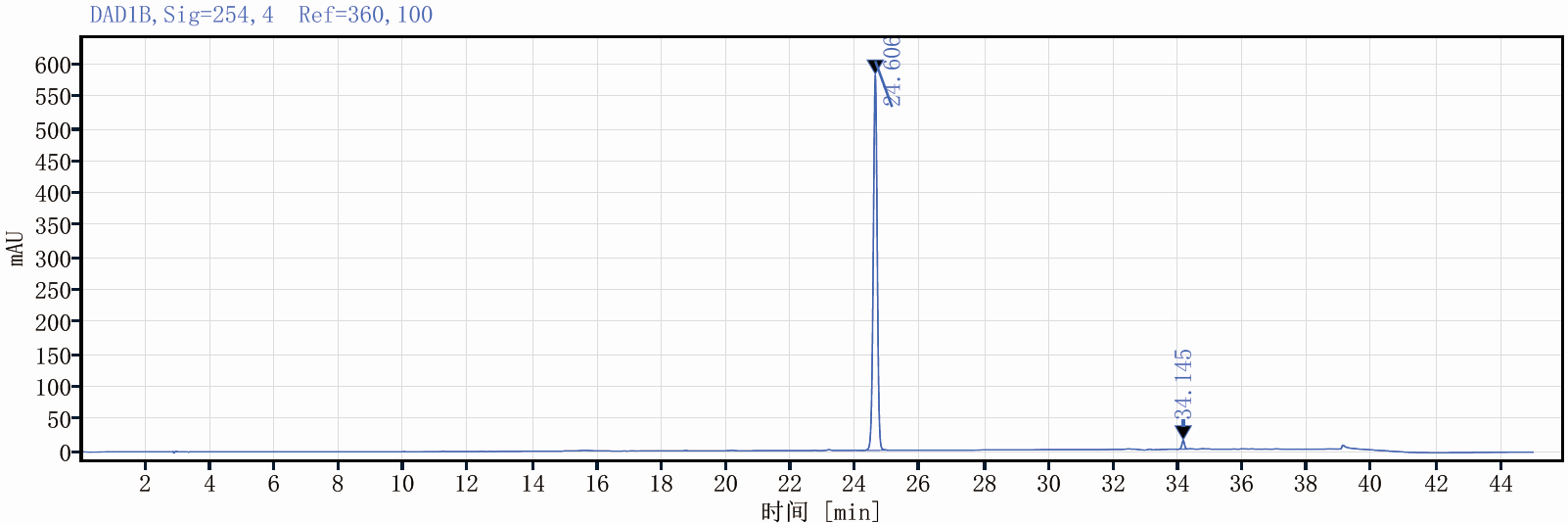


**B1** purity: 95.31%


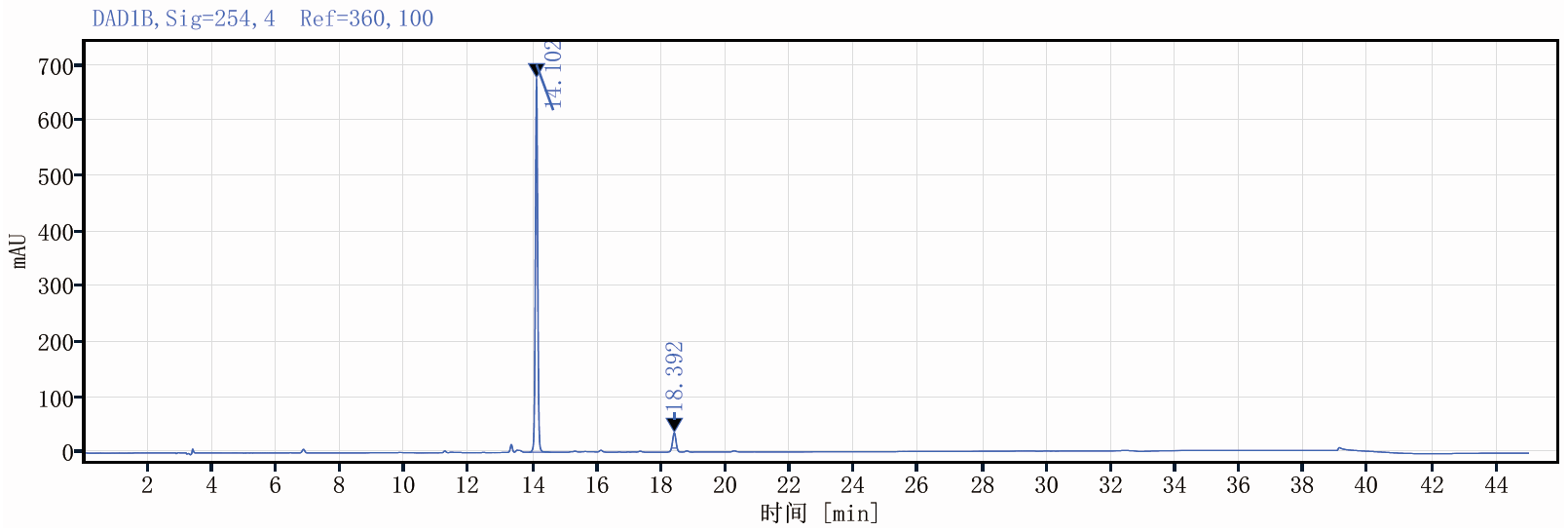


**B2** purity: 100%


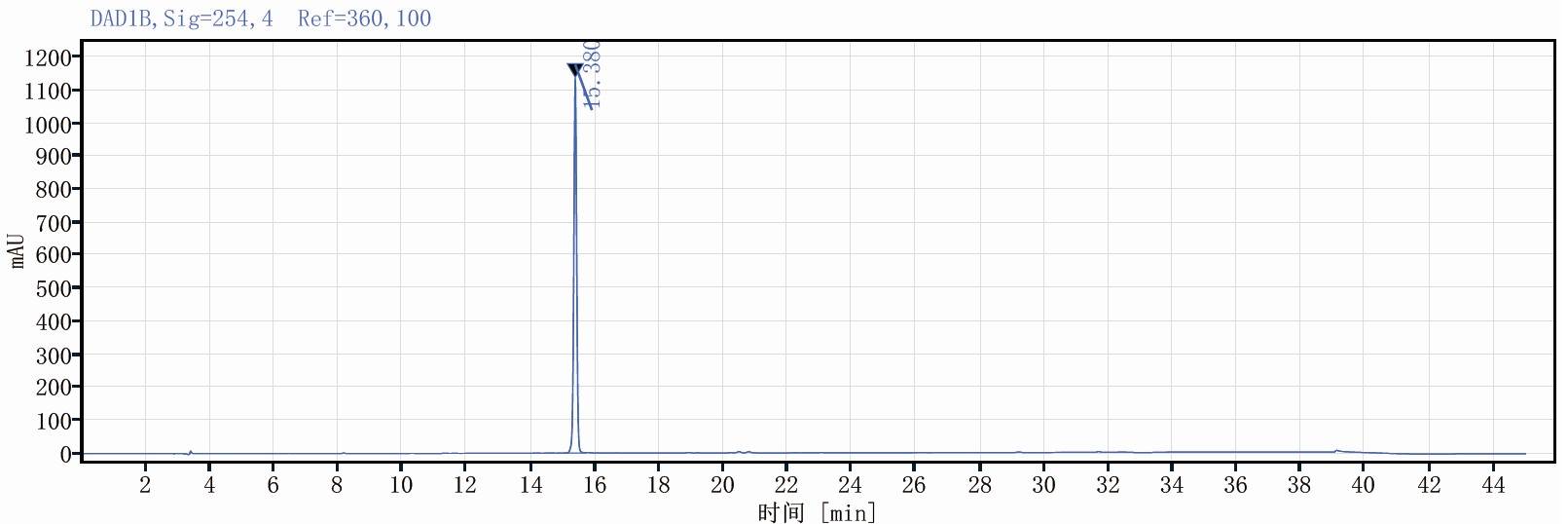


**B3** purity: 95.54%


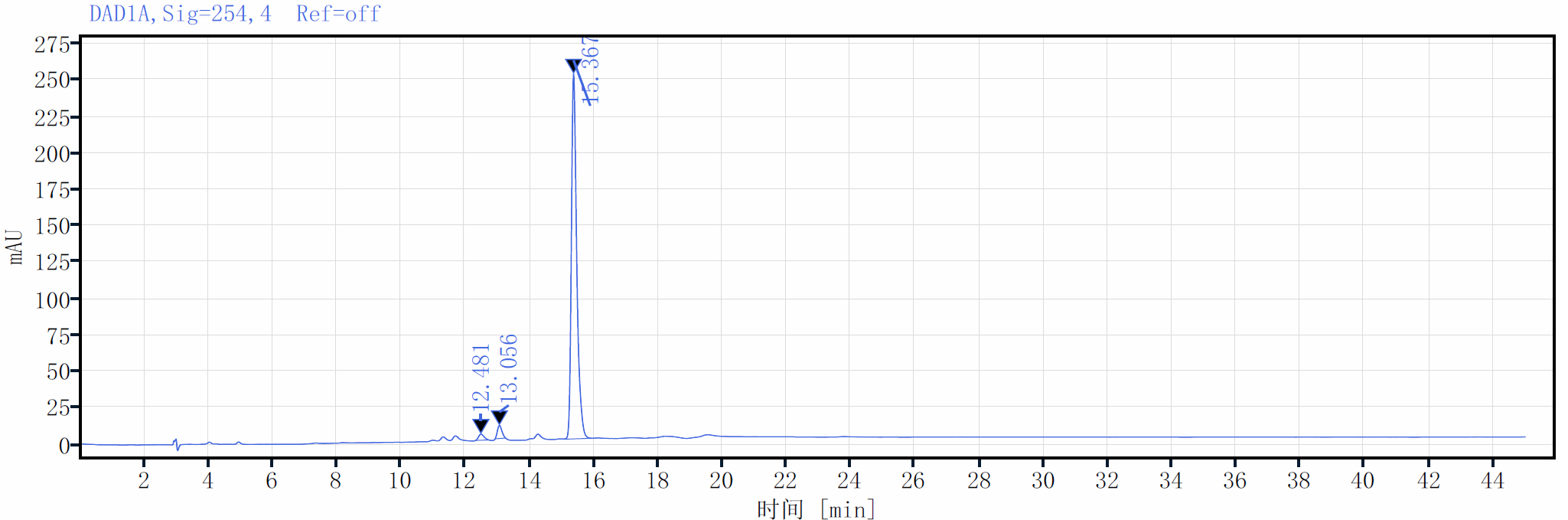


**B4** purity: 100%


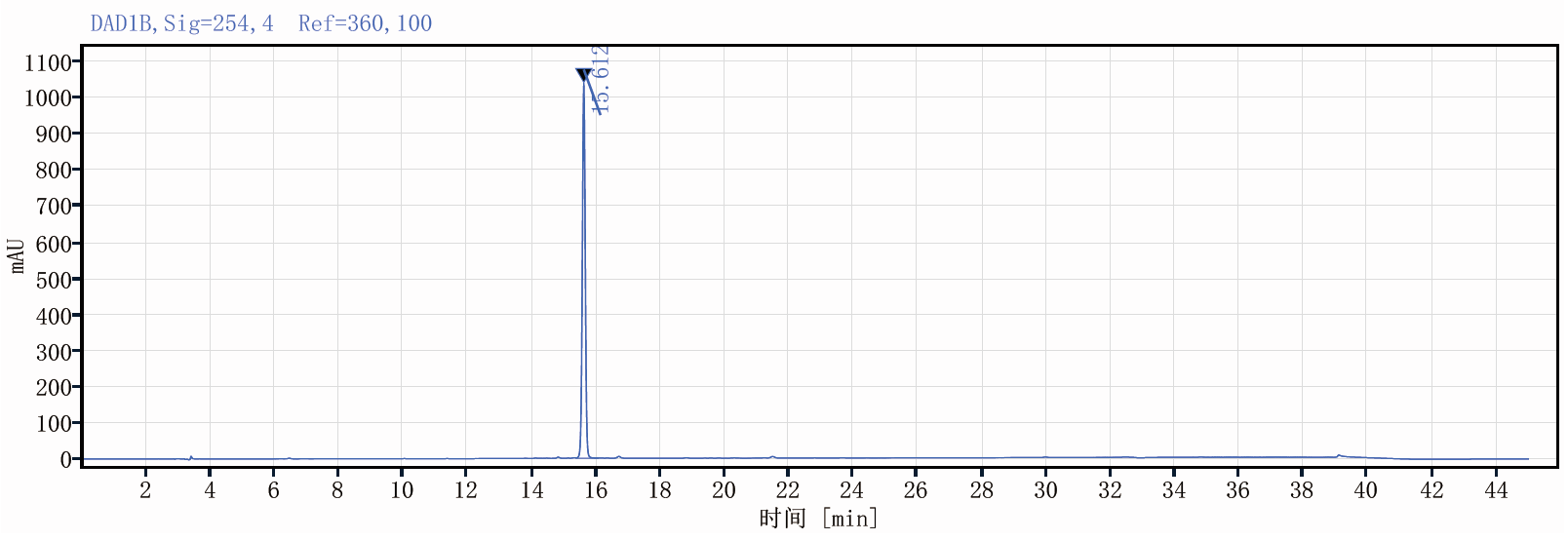


**B6** purity: 100%


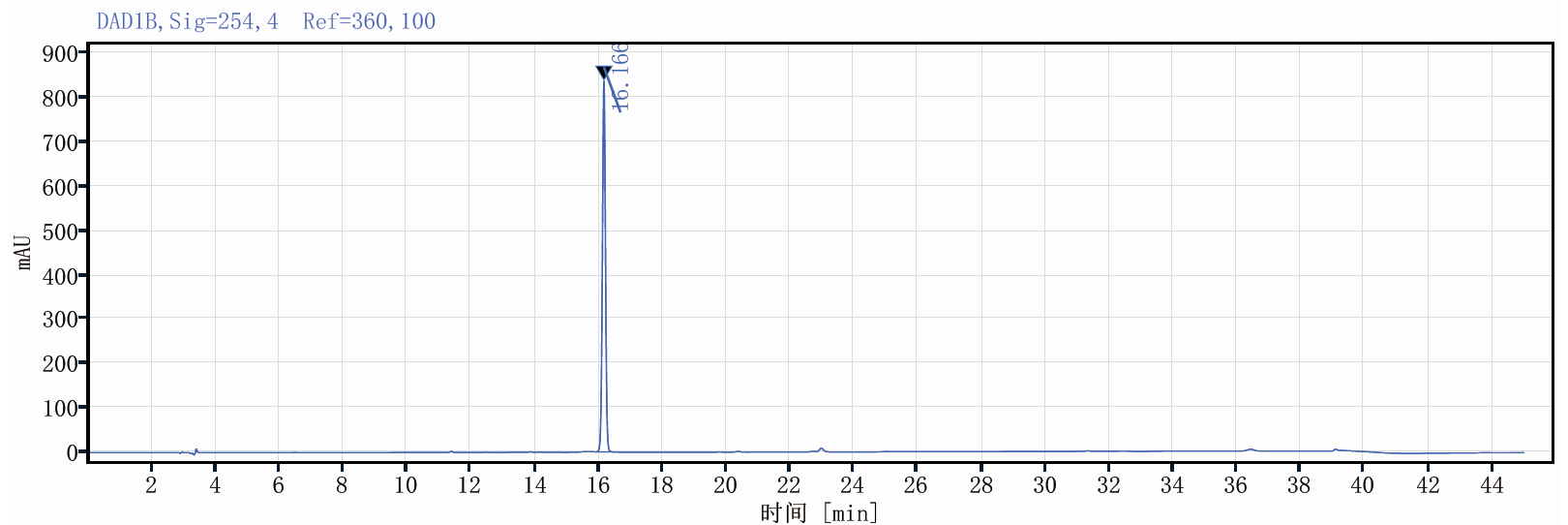


**B8** purity: 97.81%


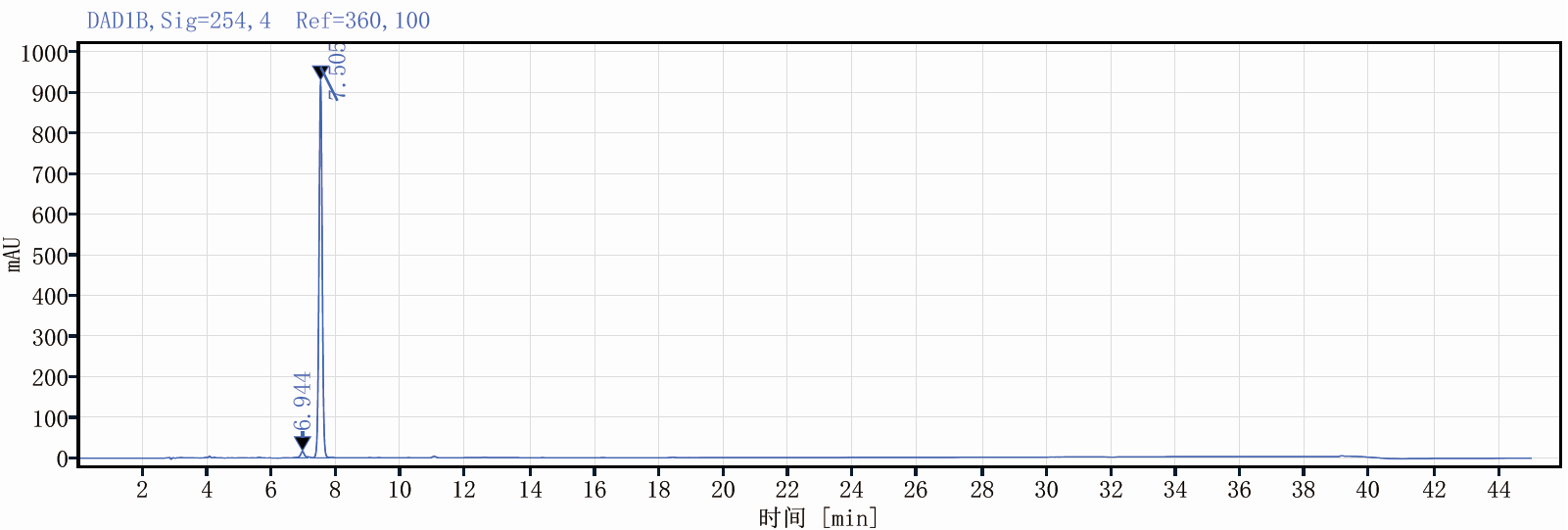


**References**

[1] a) J. J. McClure, C. Zhang, E. S. Inks, Y. K. Peterson, J. Li, C. J. Chou, *J. Med. Chem.* **2016**, *59*, 9942; b) CHOU, Chung-Jen, James, MCCLURE, Jesse, ZHANG, Cheng, INKS, Elizabeth WO2018071740.
